# Interplaying coordination and ligand effects to break or make adsorption‐energy scaling relations

**DOI:** 10.1002/EXP.20210062

**Published:** 2022-02-27

**Authors:** Alvaro Brito‐Ravicini, Federico Calle‐Vallejo

**Affiliations:** ^1^ Department of Materials Science and Chemical Physics & Institute of Theoretical and Computational Chemistry University of Barcelona Barcelona Spain

**Keywords:** adsorption‐energy scaling relations, energy‐decomposition analysis, ligand effects, near‐surface alloy, outer electrons, surface coordination

## Abstract

The linear relations between adsorption energies are one of the cornerstones of contemporary catalysis in view of the simplicity and predictive power of the computational models built upon them. Despite their extensive use, the exact nature of scaling relations is not yet fully understood, and a comprehensive theory of scaling relations is yet to be elaborated. So far, it is known that scalability is dictated by the degree of resemblance of the adsorbed species. In this work, density functional theory calculations show that CO and OH, two dissimilar species, scale or not depending on the surface facet where they adsorb at Pt alloys. This peculiar behavior arises from an interplay of ligand and geometric effects that can be used to modulate adsorption‐energy scalability. This study opens new possibilities in catalysis, as it shows that surface coordination is a versatile tool to either balance or unbalance the similarities among adsorbates at alloy surfaces.

## Graphical abstract

Currently, it is widely accepted that adsorption‐energy scaling relations exist or not depending on the similarity of the adsorbates. Here, we show that CO and OH scale linearly or not on Pt alloys depending on the coordination of the adsorption sites. Hence, ligand and geometric effects can be used to modulate scaling relations, thereby opening new possibilities in catalysis.

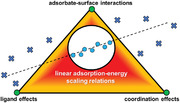

## 1 INTRODUCTION

Adsorption‐energy scaling relations are one of the pillars of contemporary catalysis.^[^ ^1^, ^2^, ^3^, ^4^ ^]^ In view of their conceptual simplicity, predictive power, and applicability over countless materials and adsorbed species, their use is widespread in computational catalysis.^[^ ^5^, ^6^, ^7^, ^8^, ^9^, ^10^ ^]^ Scaling relations between adsorbed species 1 and 2 obey the following equation:

(1) $$\Delta G_{1}=\;\gamma \Delta G_{2}+\xi$$

Although negative in specific cases,^[^ ^11^ ^]^ the slope (γ) is most often positive and given by electron‐counting rules.^[^ ^1^, ^12^ ^]^ In turn, the offset (ξ) is given by nearest‐neighbor counting rules, such that increasingly negative offsets are generally observed as the coordination number of the adsorption sites decreases (except when γ=1, in which case the offset is constant).^[^ ^13^, ^14^, ^15^ ^]^ While the initial works on scaling relations were mostly devoted to their establishment among different adsorbates on various materials, their breaking became the subject of extensive research for the past decade, motivated by the pioneering works of Koper^[^ ^16^ ^]^ and Nørskov, Rossmeisl, and coworkers.^[^ ^17^, ^18^ ^]^ Indeed, currently the breaking of scaling relations is a leitmotif in several branches of catalysis.^[^ ^19^, ^20^, ^21^, ^22^, ^23^, ^24^, ^25^ ^]^

Breaking scaling relations is, thus, widely regarded as the quintessential ingredient for efficient catalysis. However, this notion is to be taken with great caution, as previous studies have shown that breaking scaling relations is a necessary yet insufficient condition for optimal electrocatalysis,^[^ ^26^, ^27^ ^]^ it can have no impact on the catalytic activity or in fact be counterproductive,^[^ ^26^, ^28^ ^]^ and the scalability broken in vacuum can be restored in presence of a solvent.^[^ ^29^ ^]^

While numerous strategies have been proposed for breaking or circumventing scaling relations,^[^ ^19^, ^20^, ^21^, ^22^, ^23^, ^25^, ^30^ ^]^ a simple and inexpensive method to do so with experimentally verifiable results is yet to be found. In this article, we show that an interplay of ligand and coordination effects governs adsorption‐energy scalability on near‐surface alloys (NSAs) of Pt and transition metals. These alloys are known to display intriguing adsorption behaviors^[^ ^31^, ^32^ ^]^ and salient (electro)catalytic activities.^[^ ^33^, ^34^ ^]^

In terms of adsorbed species, we consider *CO and *OH, a choice justified by (i) their simultaneous involvement in numerous electrocatalytic reactions, such as methanol electrooxidation,^[^ ^35^, ^36^ ^]^ CO electrooxidation,^[^ ^37^, ^38^, ^39^ ^]^ and CO_2_ reduction to C_1_^[^ ^17^ ^]^ or C_2_^[^ ^40^ ^]^ products, just to name a few; and (ii) their unusual scalable/non‐scalable behavior. Indeed, C‐bound and O‐bound species do not regularly scale with each other,^[^ ^35^, ^41^ ^]^ which is true for *CO and *OH among different metals regardless of their surface coordination,^[^ ^17^, ^36^, ^40^, ^41^ ^]^ but does not apply for different facets of a given metal.^[^ ^39^ ^]^

Here we observe that *CO and *OH on NSAs scale or not depending on the coordination of the active sites. This peculiar phenomenon is rationalized on the basis of a simple model in which the total adsorption energy is decomposed into ligand and coordination contributions. The model indicates that surface coordination can be used to compensate for the adsorption‐induced mismatches in the electronic structure that prevent energetic scalability.

## 2 RESULTS AND DISCUSSION

Section S1 in the Supporting Information provides a full description of the density functional theory calculations shown in this work. To elucidate how coordination and ligand effects modulate scaling relations, we analyzed *CO and *OH adsorbed on Pt NSAs.

**FIGURE 1:**
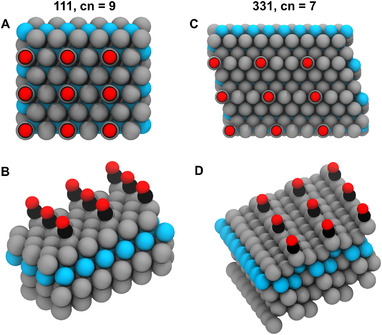
Adsorption configuration of *CO on near‐surface alloys. (A) and (B) correspond to the top and side views of a (111) near‐surface alloy, while (C) and (D) correspond to a (331) near‐surface alloy. *CO is adsorbed atop via its C atom in all cases. Grey spheres represent Pt, cyan is used for the guest atom in the subsurface layer, black for C, and red for O.

Figure 1 shows different views of *CO adsorbed on the two different surfaces considered, namely the (111) and (331) facets of Pt NSAs. The different views for *OH are available in the Supporting Information (Figure S1). We note that consistent experimental and computational evidence^[^ ^42^, ^43^, ^44^ ^]^ has shown that these two adsorbates tend bind to top sites at late‐transition metal surfaces (Pt in particular) in aqueous solution.

The geometric environments at (111) terrace sites and step‐edge sites at (331) facets differ considerably, which is captured to a first approximation by their coordination numbers, namely, 9 vs. 7. Apart from modifying the actual values of the adsorption energies (see Tables S2 and S3), we will show in the following that this coordination change has a strong impact on the energetic scalability of *CO and *OH.

**FIGURE 2:**
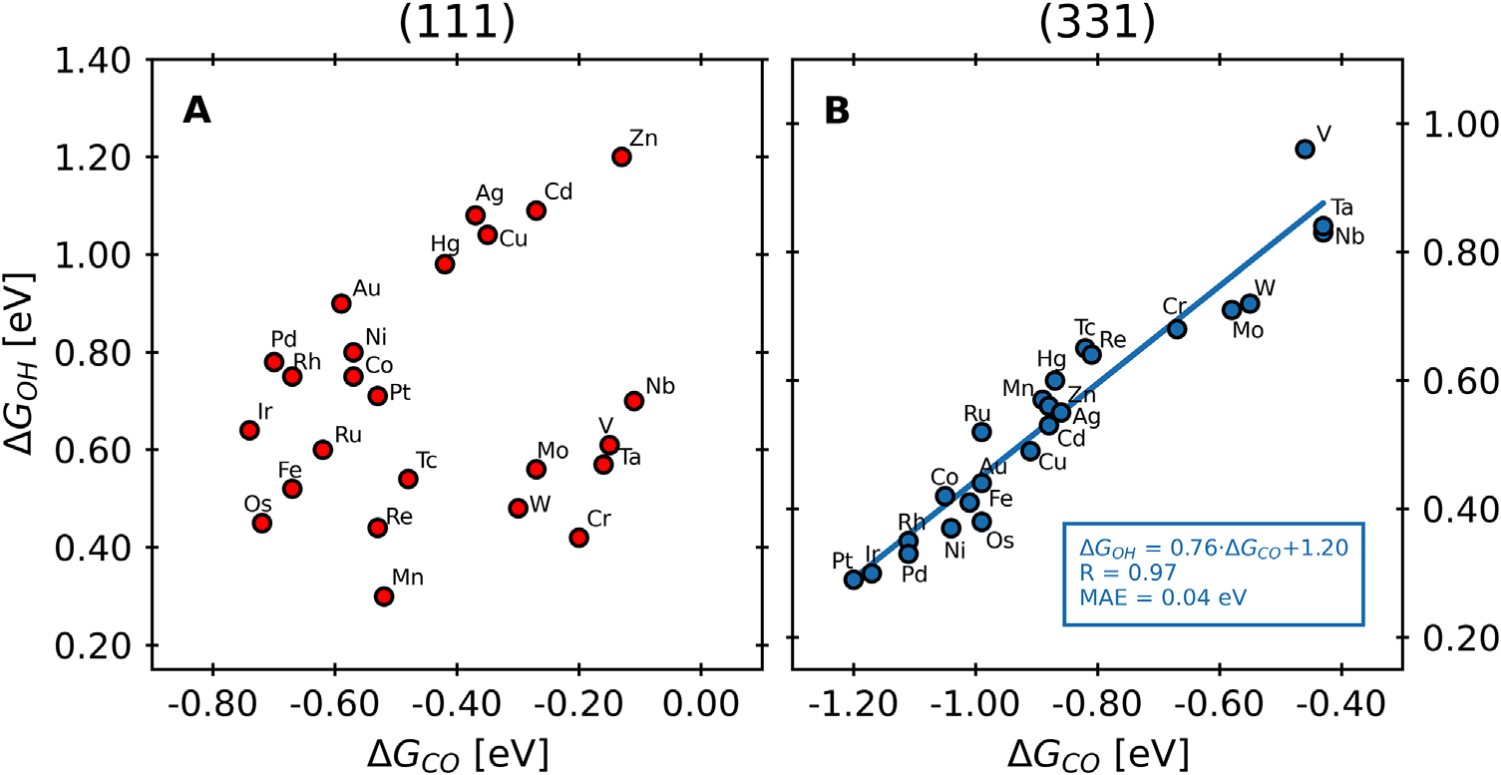
Adsorption energies of *OH as a function of those of *CO for two different surface facets of Pt NSAs. The adsorbates do not scale linearly on the (111) facet, while there is a linear relation between them on the (331) facet. The labels indicate the guest atom at the subsurface of the alloys. MAE: Mean absolute error between the calculated datapoints and the linear fit.

Indeed, when the adsorption energies of *OH ($\Delta G_{OH}$) are plotted as a function of those of *CO ($\Delta G_{CO}$), we observe two distinct behaviors depending on the coordination of the surface sites, see Figure 2. In line with the results for pure transition metals,^[^ ^17^, ^36^, ^40^ ^]^ for (111) NSAs there is no linear scaling relation between *OH and *CO. Conversely, the scaling relation for (331) NSAs is evident. Therefore, since *CO and *OH on the same series of alloys scale linearly or not depending on the coordination of the surface facets, our conclusion is that scalability is not intrinsic to a given pair of adsorbates, and surface‐related factors are also in play.

**FIGURE 3:**
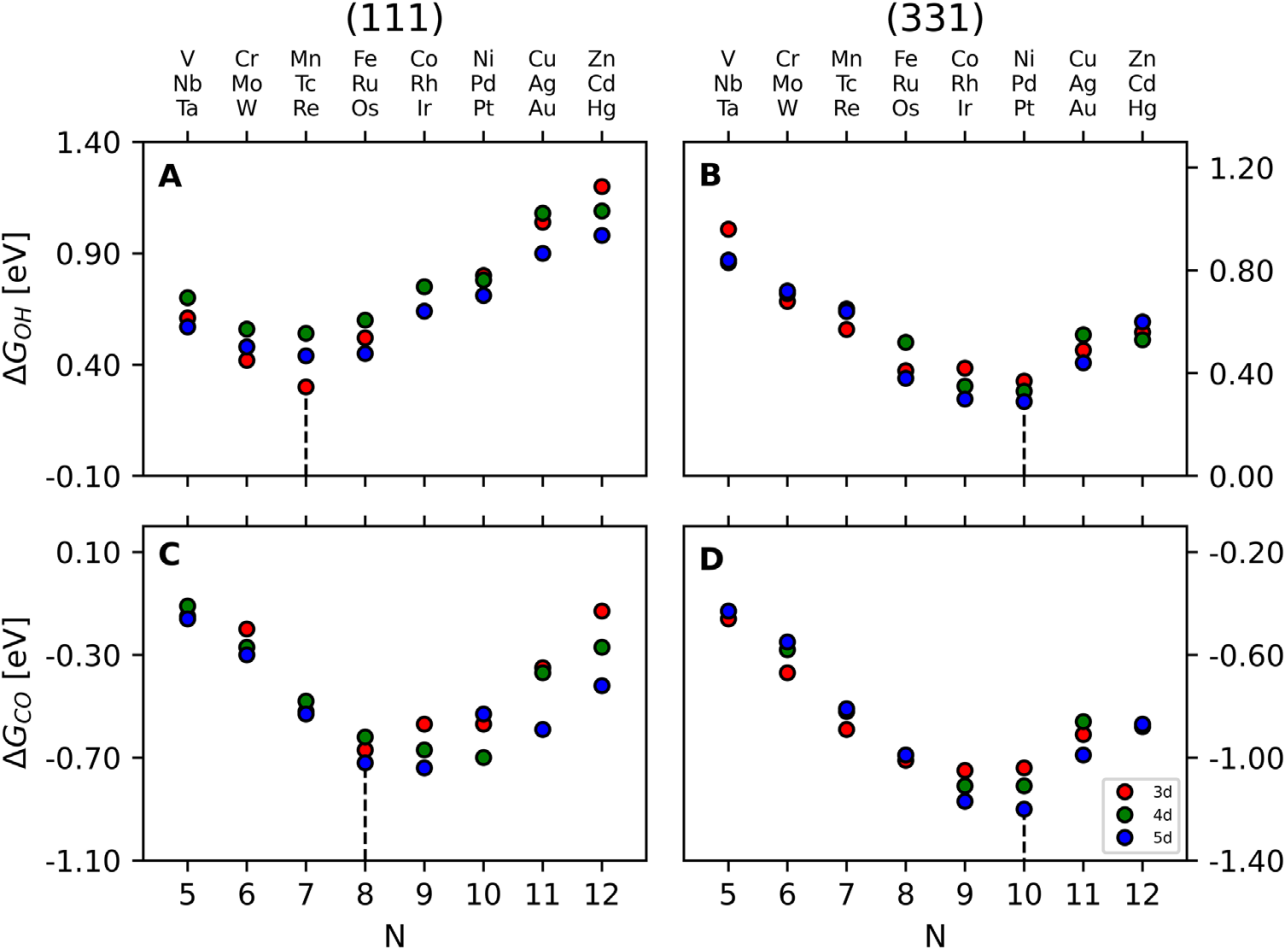
Trends in adsorption energies of *OH and *CO (top and bottom panels, respectively) for (111) and (331) near‐surface alloys (left and right panels, respectively). The datapoints have different coloring depending on the d series of the guest atom (3d metals: red, 4d metals: green, 5d metals: blue). The minima are indicated in each case by a dotted line. For *CO adsorption at (111) terraces the minima are in the range of 8–10 electrons; however, alloys with N=8 fulfill the 8‐ and 18‐electron rules. See further details in Section S3.

This conclusion can be rationalized considering that there exists a set of electronic‐structure parameters $\left\{\omega_{j}\right\}$ that is linked to the adsorption energies of *OH and *CO via functions f and g as follows:

(2) $$\Delta G_{\mathrm{OH}}^{111}=f^{111}\left(\left\{\omega_{j}\right\}\right)+\alpha_{111}$$

(3) $$\Delta G_{\mathrm{CO}}^{111}=g^{111}\left(\left\{\omega_{j}\right\}\right)+\beta_{111}$$

where $\alpha_{111}$ and $\beta_{111}$ are constants. The set $\left\{\omega_{j}\right\}$ comprises parameters such as surface outer electrons, band centers, work functions, electronic charges on the adsorbates, integrated orbital populations, etc.^[^ ^11^, ^45^ ^]^ If there is no scaling relation between *CO and *OH on the (111) facet, it is because: $f^{111}\left(\left\{\omega_{j}\right\}\right)\neq \gamma g^{111}\left(\left\{\omega_{j}\right\}\right)$. The opposite is true for the adsorption energies on the (331) facet, that is, $f^{331}\;\left(\left\{\omega_{j}\right\}\right)=\gamma g^{331}\;\left(\left\{\omega_{j}\right\}\right)$. In other words, for scalability to happen, f and g should have extrema (i.e., minima, maxima and saddle points) at the same values of $\left\{\omega_{j}\right\}$.^[^ ^12^ ^]^ In this order of ideas, Figure 3 has two noteworthy features:

1. A key parameter in set $\left\{\omega_{j}\right\}$ is the number of outer electrons (N) of the guest atoms in the NSAs. This parameter is easily assessed: for instance, the electronic distribution of Ni ends as 4s^2^ 3d^8^, so $N_{Ni}=\;2+8\;=\;10$. Based on previous studies,^[^ ^12^ ^]^ the minima of the (111) facet correspond to alloys in which all components display ideal gas configurations. In other words, as shown in Figure S2, the alloys marked in Figure 3 have metal components that fulfill the 18‐electron rule, whereas the adsorbates fulfill the octet rule. In accordance with this model, the minima should lie at N=7 for *CO, and N=8 for *OH. For (111) NSAs, this simple approach predicts successfully the most stable configuration for *OH and several other adsorbates.^[^ ^12^ ^]^ For *CO, the minima for 3d metals fits the prediction, while 4d and 5d metals have minima in the vicinity and the energetic deviations are minor. In fact, the values in Table S2 show only small differences among the expected and actual minima (0.08 eV for 4d metals and 0.02 eV for 5d metals), which might stem from surface relaxation effects.
2. The minima do not coincide for *OH and *CO adsorption energies on (111) NSAs, but match on (331) NSAs (N=10). This implies that the 8‐ and 18‐electron rules do not apply straightforwardly on the latter facet and additional effects must be present on it. Intuitively, such effects are due to the dissimilar coordination of the active sites (cn=9vs7).

We noted before that $f^{331}\;\left(\left\{\omega_{j}\right\}\right)=\gamma g^{331}\;\left(\left\{\omega_{j}\right\}\right)$ and $f^{111}\left(\left\{\omega_{j}\right\}\right)\neq \gamma g^{111}\left(\left\{\omega_{j}\right\}\right)$, because the minima in Figure 3 are the same for (331) NSAs but do not match for (111) NSAs. If $f^{111}$ and $g^{111}$ differed by a constant value, their extrema would still be the same, as the first derivative of a constant is zero. Therefore, the two functions should, in general, differ by an nth‐order polynomial or any other type of function. In this order of ideas, the least difference is given by a linear function of N:

(4) $$f^{111}\;\left(N\right)=\gamma g^{111}\;\left(N\right)+\lambda N+\kappa$$

where λ and κ are constants. If Equation (4) were valid, the effect of coordination on the NSAs (that is, the change from cn=9to7), should also be linear and the slope negative, because the minima in Figure 3 shifts from 7–8 to 10.

In the following, we will verify the hypothesis in Equation (4) by means of an energy‐decomposition analysis.^[^ ^46^, ^47^, ^48^ ^]^ We decomposed the energy into ligand and coordination effects, $\Delta G_{ligand,i}$ and $\Delta G_{coord,i}$, for *i* = *OH, *CO. In addition, we referenced all energies to those on pure Pt(111):

(5) $$\Delta G_{\mathrm{ligand},\;i}=\Delta G_{i}^{111}\;-\Delta G_{i}^{111,\;Pt}$$

(6) $$\Delta G_{\mathrm{coord},i}=\Delta G_{i}^{331}\;-\Delta G_{i}^{111\;}$$

(7) $$\Delta G_{\mathrm{total},i}=\Delta G_{\mathrm{ligand},\;i}\;+\Delta G_{\mathrm{coord},\;i}=\Delta G_{i}^{331}\;-\Delta G_{i}^{111,\;\mathrm{Pt}}$$

Ligand effects (Equation 5) account for the adsorption‐energy differences between NSAs and Pt at the (111) facet, whereas coordination effects (Equation 6) account for the adsorption energy differences for a given alloy in the two different coordination environments. Because energy‐decomposition models must be energy‐conserving, Equation (7) is the sum of Equations (5) and (6).

**FIGURE 4:**
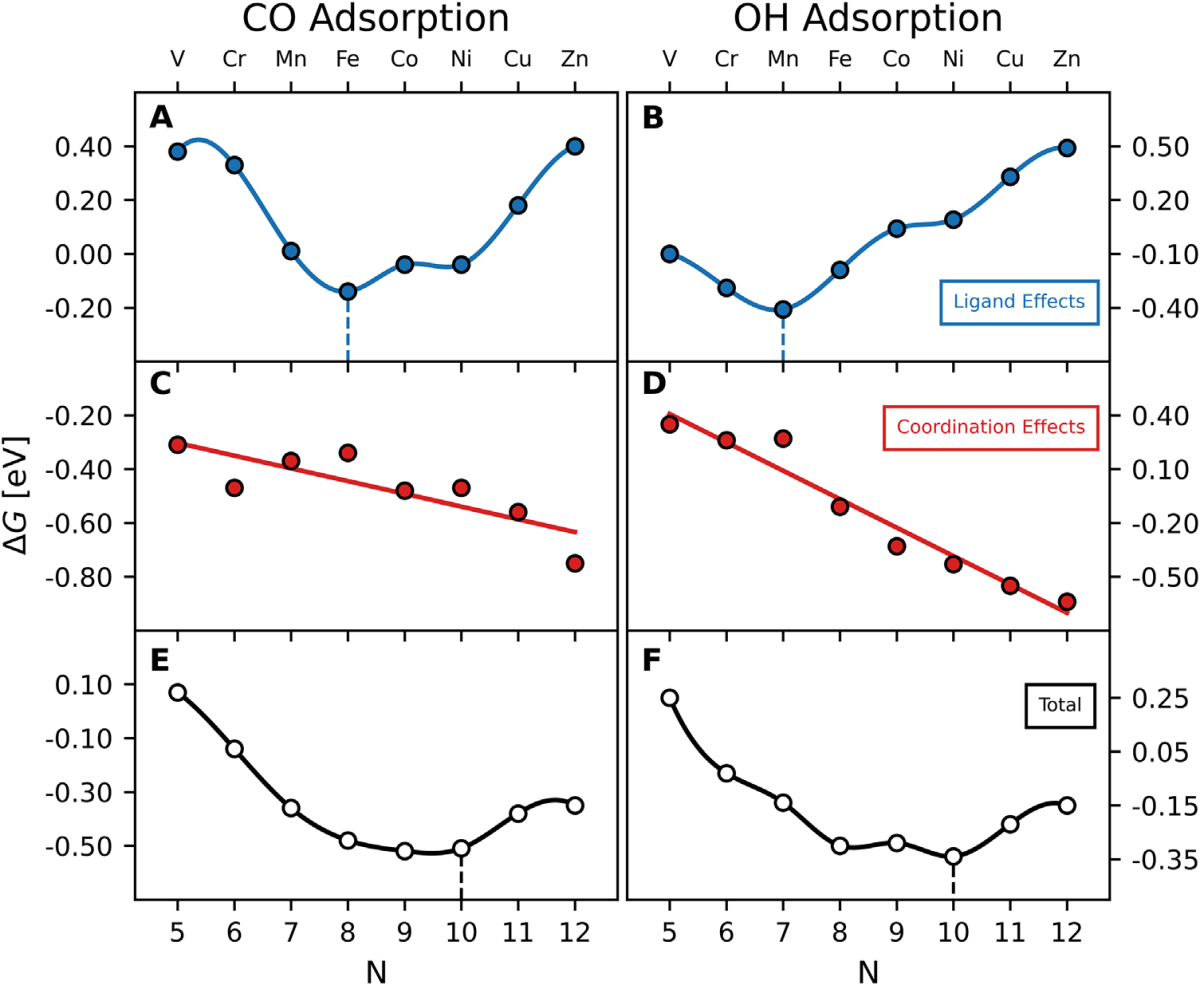
Energy‐decomposition analysis for *CO (left) and *OH (right) adsorption on the (331) facet of NSAs with 3d metals. (A,B): ligand effects for *CO and *OH calculated with respect to Pt(111) (Equation 5). (C,D): coordination effects calculated as the energetic difference between (331) and (111) facets (Equation 6). (E,F): calculated adsorption energies of the NSAs with respect to Pt(111), which result from the combination of ligand and coordination effects (Equation 7). Coordination effects (middle panels) can be fitted to a straight line as a function of the number of valence electrons of the guest atoms, while cubic splines are used to guide the eye through the trends in ligand effects (top panels) and overall adsorption energies with respect to Pt(111) (bottom panels). The equations of the linear fits and their associated mean absolute errors (MAEs) are: $\Delta G_{\mathrm{coord},\mathrm{OH}}=\;-0.16N+1.19$ (MAE = 0.06 eV), and $\Delta G_{\mathrm{coord},\mathrm{CO}}=\;-0.05N-0.06$ (MAE = 0.06 eV).

We plotted the results of Equations (5)–(7) in Figure 4 for NSAs with 3d metals. The top panels show the ligand effects (Equation 5), the trends of which are forcedly analogous to those for (111) NSAs in Figure 3. The bottom panels show the “total” adsorption energy (Equation 7), the trends of which are forcedly analogous to those for (331) NSAs in Figure 3. The middle panels reveal that coordination effects (Equation 6) are approximately a linear function of N for both *CO and *OH. Moreover, regardless of the adsorbate, coordination effects have a negative slope. This confirms the hypothesis in Equation (4), namely, that $f^{111}$ and $g^{111}$ differ at least by a linear function of N. Mathematically, this is equivalent to saying that: $\Delta G_{\mathrm{coord},i}=\theta_{i}\;N+\delta_{i}$, with $\theta_{\mathrm{OH}}>\theta_{\mathrm{CO}}$, because the minima appears earlier in the d series for *OH compared to *CO on the (111) facet and coincides in the (331) facet. Furthermore, $\theta_{i}$ and $\delta_{i}$ are such that:

(8) $$\Delta G_{\mathrm{coord}}=\;\gamma \Delta G_{\mathrm{coord},\mathrm{CO}}-\Delta G_{\mathrm{coord},\mathrm{OH}}=\;-\lambda N-\kappa$$

We note that Equation (4) provides the least difference between non‐scalable adsorbates, so that the difference might as well be quadratic, cubic, etc. Combining Equations (2), (3) and ((5), (6), (7)), we can conclude the following about the functions f and g on two different facets:

(9) $$f^{331}\left(N\right)\;=f^{111}\;\left(N\right)+\phi_{OH}+\Delta G_{\mathrm{coord},\mathrm{OH}}$$

(10) $$g^{331}\left(N\right)\;=g^{111}\;\left(N\right)+\phi_{CO}+\Delta G_{\mathrm{coord},\mathrm{CO}}$$

where $\phi_{OH}=\alpha_{111}\;-\alpha_{331}$ and $\phi_{\mathrm{CO}}=\beta_{111}\;-\beta_{331}$. We close this section stressing that $\Delta G_{coord}$ being a linear function of *N* for *OH vs. *CO (Equation 8) implies that changes in coordination affect the NSAs differently. Indeed, the negative slopes in Figure 4 indicate that guests with high d‐band fillings induce large stabilizations when the coordination of the adsorption sites changes from 9 to 7. Finally, the trends for *CO and *OH adsorption on the NSAs of Pt(100) and a comparison of the scaling relations on the (111), (100) and (331) facets can be found in Section S4.

## 3 CONCLUSIONS

In the quest for enhanced catalysts, numerous strategies have been devised in the past decade for breaking scaling relations. Here we showed that by interplaying coordination and ligand effects one can break or restore at will the *OH vs. * CO scaling relation for near‐surface alloys of Pt and transition metals. Interestingly, this phenomenon stems from specific differences in the shape of the functions that define the separate adsorption energies.

While all this hints toward the use of single‐crystalline catalysts to obtain desired effects (either scalability or non‐scalability), our analysis also indicates that polycrystalline surfaces presumably have scalable and non‐scalable regions. In such case, either fostering or preventing surface diffusion, namely, allowing the adsorbates to move or not from one region to another, might be advisable to enhance the catalytic activity. Future studies could assess this hypothesis by using suitable surface facets comprising long terraces and defects such as steps and/or kinks.

We expect our conclusions to be relevant for (i) the electro‐oxidation of CO, hydrocarbons, and alcohols, where *CO and *OH are formed and recombined to produce CO_2_, and (ii) CO_2_ and CO electroreduction, where *CO and *OH are formed along the catalytic pathways toward methane and ethylene.

## 4 METHODS

Full details of the density functional theory calculations shown in this work can be found in Section S1 in the Supporting Information.

## CONFLICT OF INTEREST

The authors declare no conflict of interest.

## Supporting information



Supporting Information
