# Supplementary material for: Interplaying coordination and ligand effects to break or make adsorption‐energy scaling relations
Source: Exploration (Beijing). 2022 Feb 27;2(2):20210062. doi: 10.1002/EXP.20210062 (PMC10191023; doi:10.1002/EXP.20210062)
Supplement: Supplementary file 1 — Supporting Information [file EXP2-2-20210062-s001.pdf]

Supporting Information

Interplaying Coordination and Ligand Effects on Near-Surface Alloys of Pt to Break or Make Adsorption-Energy Scaling Relations

Alvaro Brito-Ravicini and Federico Calle-Vallejo*

Department of Materials Science and Chemical Physics & Institute of Theoretical and Computational Chemistry, University of Barcelona, Martí i Franquès 1, 08028 Barcelona, Spain.

Email: f.calle.vallejo@ub.edu

Table of contents

S1. Computational methods and tabulated energies **2**

S2. Adsorption Configuration of *OH **5**

S3. Lewis Diagrams **6**

S4. Results for Pt(100) NSAs **6**

S5. Direct coordinates **8**

References **18**

S1. Computational methods and tabulated energies

The DFT calculations were performed with the Vienna Ab Initio Simulation Package (VASP).[1] To represent ion-electron interactions, we used the projector augmented-wave (PAW) method.[2] The Perdew-Burke-Ernzerhof (PBE) exchange-correlation functional was used to calculate the total energies.[3] The near-surface alloys (NSAs) were modeled as a 4-metal layer slab in which the second layer was filled with a guest atom (3d elements from V to Zn, 4d elements from Nb to Cd, 5d elements from Ta to Hg), while the rest of the surface was occupied by Pt (see section S4). The top two layers were allowed to relax, while the others were kept fixed at the optimized bulk positions of Pt, for which we found a converged lattice constant of 3.98 Å. The separation between periodical images in the vertical axis was larger than 14 Å, which suffices to avoid spurious electrostatic interactions between them.

The relaxations were computed using a plane-wave cutoff of 450 eV and an electronic temperature of 0.2 eV, and all energies were extrapolated to 0 K. The conjugate gradient method was employed to minimize the maximum forces on the atoms, until the maximal force was below 0.05 eV Å^{-1}. To sample the k-space we used Monkhorst-Pack grids[4] of (6×7×1) and (4×5×1) for the (2×2) (111) and (3×1) (331) slabs, respectively. The free molecules (CO, CO_2, H_2O and H_2) were modelled in cubic boxes of 15 Å × 15 Å × 15 Å with an electronic temperature of 0.001 eV and sampling only the Γ-point in k-space.

The free energies were approximated as: $G \approx E_{DFT} + ZPE - TS + E_{solv}$, where E_{DFT} is the total energy calculated with DFT; ZPE is the zero-point energy obtained from DFT-based vibrational analyses within the harmonic oscillator approximation; TS are the entropy corrections for the free molecules taken at T = 298.15 K, and those for the adsorbates were neglected, as a first

approximation; and E_{solv} are the water-adsorbate stabilization effects, the values of which for *CO and *OH were extracted from previous studies (see Table S1).[5,6] Water was simulated as a gas, but its energetics are provided in the liquid phase, which is made by means of an entropy correction.[6,7] To calculate the free energy of proton-electron pairs, the computational hydrogen electrode was used.[7] This allowed us to describe such complicated energetics in solution in terms of that of H_2 in the gas phase. All the energetic corrections and free energies of adsorption in this study are shown in Tables S1, S2 and S3.

Table S1. Corrections needed to assess the free energies of the molecules and adsorbates in this study. The ZPEs of *OH and *CO appear in Tables S2-S4.

Species	ZPE [eV]	TS [eV]	E_{solv} [eV]
CO(g)	0.13	0.61	0.00
CO_2(g)	0.31	0.66	0.00
H_2(g)	0.28	0.40	0.00
H_2O(l)	0.58	0.67	0.00
*OH	–	0.00	-0.58
*CO	–	0.00	-0.10

Table S2. Zero-point energies and free energies of adsorption of *CO and *OH on the (111) facet of Pt NSAs.

Alloy	ZPE_{CO} [eV]	ZPE_{OH} [eV]	ΔG_{CO} [eV]	ΔG_{OH} [eV]
Pt-V	0.20	0.33	-0.15	0.61
Pt-Cr	0.21	0.33	-0.20	0.42
Pt-Mn	0.21	0.33	-0.52	0.30
Pt-Fe	0.21	0.33	-0.67	0.52
Pt-Co	0.21	0.33	-0.57	0.75
Pt-Ni	0.21	0.33	-0.57	0.80
Pt-Cu	0.21	0.33	-0.35	1.04
Pt-Zn	0.20	0.32	-0.13	1.20
Pt-Nb	0.20	0.32	-0.11	0.70

Pt-Mo	0.20	0.33	-0.27	0.56
Pt-Tc	0.21	0.33	-0.48	0.54
Pt-Ru	0.21	0.34	-0.62	0.60
Pt-Rh	0.21	0.34	-0.67	0.75
Pt-Pd	0.21	0.34	-0.70	0.78
Pt-Ag	0.20	0.33	-0.37	1.08
Pt-Cd	0.20	0.32	-0.27	1.09
Pt-Ta	0.20	0.32	-0.16	0.57
Pt-W	0.20	0.33	-0.30	0.48
Pt-Re	0.21	0.34	-0.53	0.44
Pt-Os	0.21	0.34	-0.72	0.45
Pt-Ir	0.21	0.34	-0.74	0.64
Pt-Pt	0.21	0.34	-0.53	0.71
Pt-Au	0.21	0.34	-0.59	0.90
Pt-Hg	0.21	0.33	-0.42	0.98

Table S3. Zero-point energies and free energies of adsorption of *CO and *OH on the (331) facet of Pt NSAs.

Alloy	ZPE_{CO} [eV]	ZPE_{OH} [eV]	ΔG_{CO} [eV]	ΔG_{OH} [eV]
Pt-V	0.20	0.35	-0.46	0.96
Pt-Cr	0.20	0.32	-0.67	0.68
Pt-Mn	0.21	0.31	-0.89	0.57
Pt-Fe	0.21	0.34	-1.01	0.41
Pt-Co	0.21	0.33	-1.05	0.42
Pt-Ni	0.21	0.33	-1.04	0.37
Pt-Cu	0.21	0.33	-0.91	0.49
Pt-Zn	0.20	0.33	-0.88	0.56
Pt-Nb	0.20	0.32	-0.43	0.83
Pt-Mo	0.20	0.34	-0.58	0.71
Pt-Tc	0.21	0.31	-0.82	0.65
Pt-Ru	0.21	0.32	-0.99	0.52
Pt-Rh	0.21	0.34	-1.11	0.35
Pt-Pd	0.21	0.34	-1.11	0.33
Pt-Ag	0.21	0.34	-0.86	0.55
Pt-Cd	0.21	0.33	-0.88	0.53
Pt-Ta	0.20	0.32	-0.43	0.84
Pt-W	0.20	0.34	-0.55	0.72
Pt-Re	0.21	0.31	-0.81	0.64
Pt-Os	0.21	0.34	-0.99	0.38

Pt-Ir	0.21	0.33	-1.17	0.30
Pt-Pt	0.21	0.34	-1.20	0.29
Pt-Au	0.21	0.34	-0.99	0.44
Pt-Hg	0.21	0.34	-0.87	0.60

S2. Adsorption Configuration of *OH

In Figure S1 we show different views of *OH adsorbed on the two different surfaces considered.

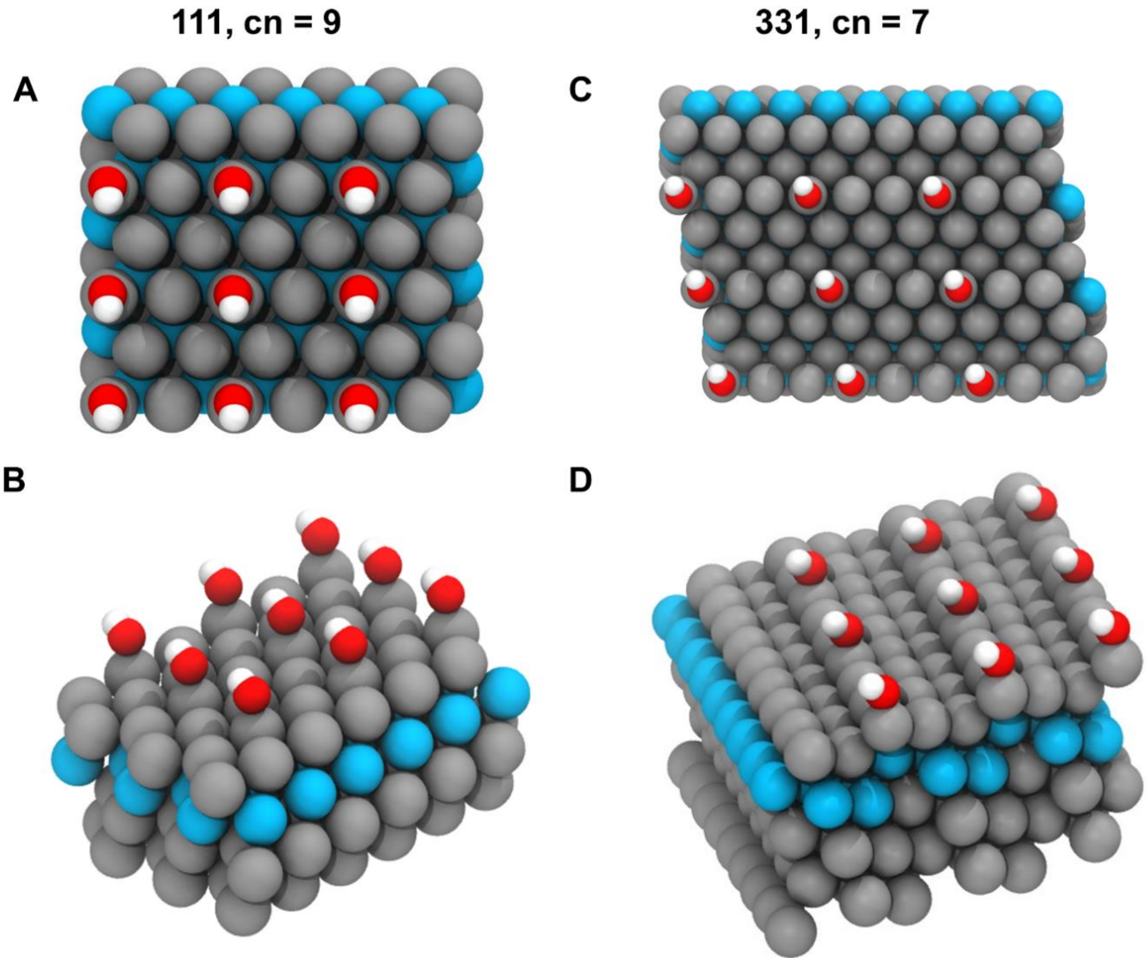

Figure S1. Adsorption configuration of *OH on near-surface alloys. (a) and (b) correspond to the top and side views of a (111) near-surface alloy, while (c) and (d) correspond to a (331) near-surface alloy. *OH is adsorbed atop in all cases. Grey spheres represent Pt, cyan is used for the guest atom in the subsurface layer, white for H and red for O.

S3. Lewis Diagrams

The 8 and 18-electron rule can be used to predict the most stable alloy configuration for a given adsorbate.[8] To identify these alloys, Lewis diagrams were constructed. The idea is to apply the 8-electron rule to the adsorbate, and the 18-electron rule to the surface. Following this procedure, Figure S2 is generated.

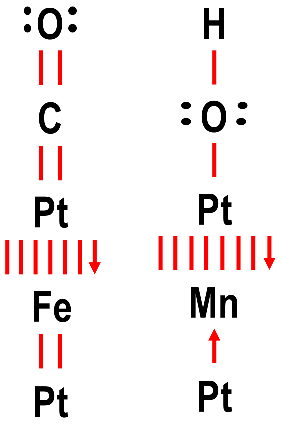

Figure S2. Most stable alloy configuration for *CO and *OH adsorption on NSAs of Pt(111). For *CO, the alloys that resemble the electronic structure of noble gases correspond to those where the guest atom has 8 valence electrons (Fe, Ru, Os). For *OH, the optimal configuration is found when the guest atom possesses 7 valence electrons (Mn, Tc, Re). The symbols indicate shared pairs of electrons (lines), lone pairs (dots) and dative bonds (arrows). The Lewis diagrams were made based on reference.[8]

S4. Results for Pt(100) NSAs

The calculations were made as described in section S1 with (2×2) 4-layer-thick slabs and a k-point sampling of (6×6×1). Again, the mismatch of the minima causes the non-scalability of *OH and *CO (Figure S3A-B). Interestingly, when the data for the three facets ((111), (100) and (331)) are plotted altogether a scaling line seems to contain most of the points (see the ellipse in Figure S3C) and there is also a region of departed data. The adsorption energies and ZPEs appear in Table S4.

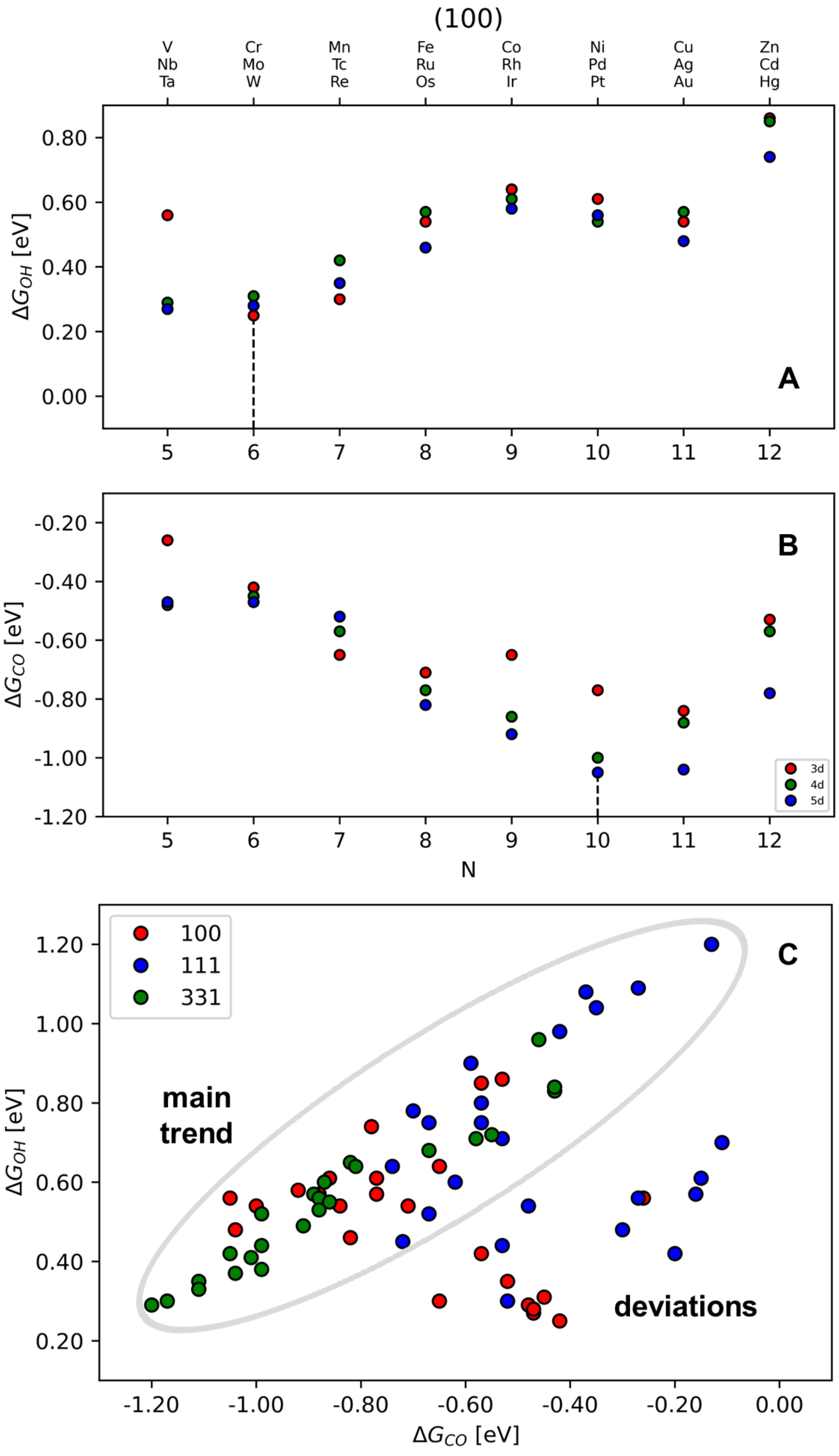

Figure S3. Trends in the adsorption energies of (A) *OH and (B) *CO on the (100) facet of NSAs of Pt and transition metals as a function of the number of outer electrons (N) of the guest atom. C) Adsorption energies of *OH plotted versus those of *CO for the (111), (100) and (331) facets of NSAs. The ellipse is drawn as a guide to the eye.

Table S4. Zero-point energies and free energies of adsorption of *CO and *OH on the (100) facet of Pt NSAs.

Alloy	ZPE_{CO} [eV]	ZPE_{OH} [eV]	ΔG_{CO} [eV]	ΔG_{OH} [eV]
Pt-V	0.19	0.33	-0.26	0.56
Pt-Cr	0.19	0.33	-0.42	0.25
Pt-Mn	0.20	0.33	-0.65	0.30
Pt-Fe	0.20	0.33	-0.71	0.54
Pt-Co	0.20	0.33	-0.65	0.64
Pt-Ni	0.20	0.33	-0.77	0.61
Pt-Cu	0.20	0.33	-0.84	0.54
Pt-Zn	0.19	0.32	-0.53	0.86
Pt-Nb	0.20	0.32	-0.48	0.29
Pt-Mo	0.19	0.33	-0.45	0.31
Pt-Tc	0.19	0.33	-0.57	0.42
Pt-Ru	0.20	0.33	-0.77	0.57
Pt-Rh	0.21	0.33	-0.86	0.61
Pt-Pd	0.21	0.33	-1.00	0.54
Pt-Ag	0.20	0.33	-0.88	0.57
Pt-Cd	0.20	0.32	-0.57	0.85
Pt-Ta	0.19	0.32	-0.47	0.27
Pt-W	0.19	0.33	-0.47	0.28
Pt-Re	0.20	0.34	-0.52	0.35
Pt-Os	0.20	0.33	-0.82	0.46
Pt-Ir	0.21	0.33	-0.92	0.58
Pt-Pt	0.21	0.33	-1.05	0.56
Pt-Au	0.21	0.34	-1.04	0.48
Pt-Hg	0.20	0.34	-0.78	0.74

S5. Direct coordinates

111 *CO

```
Pt Surface(111)-4 layers + Alloys + Adso
  1.00000000000000
    5.6235999999999997    0.0000000000000000    0.0000000000000000
    0.0000000000000000    4.8701999999999996    0.0000000000000000
    0.0000000000000000    0.0000000000000000   25.0000000000000000
  Pt   V    C    O
   12     4     1     1
Selective dynamics
Direct
  0.0000000000000000  0.0079673450471034 -0.0103086370802370   T   T   T
  0.5000000000000000  0.0048899494114219 -0.0198737402599421   T   T   T
  0.2530100200565324  0.5081753856690814 -0.0171545893307660   T   T   T
  0.7469899799434679  0.5081753856690814 -0.0171545893307660   T   T   T
  0.0000000000000000  0.3333333333333357  0.8163320000000027   F   F   F
  0.2500000000000000  0.8333333333333357  0.8163320000000027   F   F   F
  0.5000000000000000  0.3333333333333357  0.8163320000000027   F   F   F
  0.7500000000000000  0.8333333333333357  0.8163320000000027   F   F   F
  0.0000000000000000  0.0000000000000000  0.7244999999999990   F   F   F
  0.5000000000000000  0.0000000000000000  0.7244999999999990   F   F   F
  0.2500000000000000  0.5000000000000000  0.7244999999999990   F   F   F
  0.7500000000000000  0.5000000000000000  0.7244999999999990   F   F   F
  0.2469430568571671  0.1913298437006125  0.8990343351512976   T   T   T
  0.5000000000000000  0.6417527300487558  0.8985031477694645   T   T   T
  0.7530569431428354  0.1913298437006125  0.8990343351512976   T   T   T
  0.0000000000000000  0.6465239652929253  0.8983274267934108   T   T   T
  0.0000000000000000  0.0006437690246758  0.0674382765694152   T   T   T
  0.0000000000000000  0.0001480057511789  0.1137770141265101   T   T   T
Pt Surface(111)-4 layers + Alloys + Adso
  1.00000000000000
    5.6235999999999997    0.0000000000000000    0.0000000000000000
    0.0000000000000000    4.8701999999999996    0.0000000000000000
    0.0000000000000000    0.0000000000000000   25.0000000000000000
  Pt   Cr   C    O
   12     4     1     1
Selective dynamics
Direct
  0.0018456981857821  0.0002206529213010  0.9882181497726219   T   T   T
  0.5016333617870410  0.9974712146091197  0.9777980449503386   T   T   T
  0.2536343722502487  0.5002372257541973  0.9783358604263831   T   T   T
  0.7508430558032596  0.4996452680430826  0.9784930378158401   T   T   T
  0.0000000000000000  0.3333333333333357  0.8163320000000027   F   F   F
  0.2571128814282702  0.8333333333333357  0.8163320000000027   F   F   F
  0.5071128814282702  0.3333333333333357  0.8163320000000027   F   F   F
  0.7500000000000000  0.8333333333333357  0.8163320000000027   F   F   F
  0.0000000000000000  0.0000000000000000  0.7244999999999990   F   F   F
  0.5000000000000000  0.0000000000000000  0.7244999999999990   F   F   F
  0.2500000000000000  0.5000000000000000  0.7244999999999990   F   F   F
  0.7500000000000000  0.5000000000000000  0.7244999999999990   F   F   F
  0.2567944359168390  0.1699185590574938  0.8965448199280731   T   T   T
  0.5040200317768680  0.6422334413137060  0.8967509091279147   T   T   T
  0.7509101159713204  0.1656982241511600  0.8969092476478006   T   T   T
  0.0017105147884951  0.6944586266701145  0.8969388351301349   T   T   T
  0.0013962958069005  0.0048227231585038  0.0650585957857659   T   T   T
  0.9994538166474064  0.9988673568435743  0.1113903635041360   T   T   T
Pt Surface(111)-4 layers + Alloys + Adso
  1.00000000000000
    5.6235999999999997    0.0000000000000000    0.0000000000000000
    0.0000000000000000    4.8701999999999996    0.0000000000000000
    0.0000000000000000    0.0000000000000000   25.0000000000000000
  Pt   Mn   C    O
   12     4     1     1
Selective dynamics
Direct
  0.0028992116729952  0.0006854627738812  0.9879383976942030   T   T   T
  0.5029662912266533  0.0065789369469760  0.9691618832024844   T   T   T
  0.2556440674323385  0.5050220841226325  0.9766621077504198   T   T   T
  0.7498346497350392  0.5039890760311820  0.9765706809960955   T   T   T
  0.0000000000000000  0.3333333333333357  0.8163320000000027   F   F   F
  0.2500000000000000  0.8333333333333357  0.8163320000000027   F   F   F
  0.5000000000000000  0.3333333333333357  0.8163320000000027   F   F   F
  0.7500000000000000  0.8333333333333357  0.8163320000000027   F   F   F
  0.0000000000000000  0.0000000000000000  0.7244999999999990   F   F   F
  0.5000000000000000  0.0000000000000000  0.7244999999999990   F   F   F
  0.2500000000000000  0.5000000000000000  0.7244999999999990   F   F   F
  0.7500000000000000  0.5000000000000000  0.7244999999999990   F   F   F
  0.2098165351457223  0.1786058755337440  0.8969727131153434   T   T   T
  0.5020590432405102  0.6342743661307242  0.8951100891769163   T   T   T
  0.7978774753609884  0.1757412317744337  0.8973051042349101   T   T   T
  0.0020266108949021  0.6519327380261475  0.8946569327285968   T   T   T
  0.0021597268493369  0.9936806769592353  0.0638123750724893   T   T   T
  0.9995773363530386  0.0032912859445646  0.1101344444815388   T   T   T
Pt Surface(111)-4 layers + Alloys + Adso
  1.00000000000000
    5.6235999999999997    0.0000000000000000    0.0000000000000000
    0.0000000000000000    4.8701999999999996    0.0000000000000000
    0.0000000000000000    0.0000000000000000   25.0000000000000000
  Pt   Fe   C    O
   12     4     1     1
Selective dynamics
Direct
 -0.0000000000000000 -0.0065034275240828 -0.0126164728412601   T   T   T
  0.5000000000000000  0.0083494986811110 -0.0250573193645850   T   T   T
  0.2525527867539147  0.4948915785070054 -0.0300920270637671   T   T   T
  0.7474472132460854  0.4948915785070054 -0.0300920270637671   T   T   T
  0.0000000000000000  0.3333333333333357  0.8163320000000027   F   F   F
```

```
 0.2500000000000000  0.8333333333333357  0.8163320000000027   F   F   F
 0.5000000000000000  0.3333333333333357  0.8163320000000027   F   F   F
 0.7500000000000000  0.8333333333333357  0.8163320000000027   F   F   F
 0.0000000000000000  0.0000000000000000  0.7244999999999990   F   F   F
 0.5000000000000000  0.0000000000000000  0.7244999999999990   F   F   F
 0.2500000000000000  0.5000000000000000  0.7244999999999990   F   F   F
 0.7500000000000000  0.5000000000000000  0.7244999999999990   F   F   F
 0.2227220050255288  0.1256797798204609  0.8966325412580555   T   T   T
 0.5000000000000000  0.7090972252223117  0.8947297003172394   T   T   T
 0.7772779949744711  0.1256797798204609  0.8966325412580555   T   T   T
-0.0000000000000000  0.7068611388417447  0.8951009149056809   T   T   T
-0.0000000000000000 -0.0020951770430982  0.0631127114864163   T   T   T
-0.0000000000000000 -0.0001518031770146  0.1094789431581000   T   T   T
Pt Surface(111)-4 layers + Alloys + Adso
   1.00000000000000
     5.6235999999999997    0.0000000000000000    0.0000000000000000
     0.0000000000000000    4.8701999999999996    0.0000000000000000
     0.0000000000000000    0.0000000000000000   25.0000000000000000
   Pt   Co   C    O
    12     4     1     1
Selective dynamics
Direct
 0.0000000000000000 -0.0013101593781844 -0.0135721572154586   T   T   T
 0.5000000000000000  0.0039158276904662 -0.0275728796476926   T   T   T
 0.2524675373684188  0.4975409042970893 -0.0284427107043147   T   T   T
 0.7475324626315808  0.4975409042970893 -0.0284427107043147   T   T   T
 0.0000000000000000  0.3333333333333357  0.8163320000000027   F   F   F
 0.2500000000000000  0.8333333333333357  0.8163320000000027   F   F   F
 0.5000000000000000  0.3333333333333357  0.8163320000000027   F   F   F
 0.7500000000000000  0.8333333333333357  0.8163320000000027   F   F   F
 0.0000000000000000  0.0000000000000000  0.7244999999999990   F   F   F
 0.5000000000000000  0.0000000000000000  0.7244999999999990   F   F   F
 0.2500000000000000  0.5000000000000000  0.7244999999999990   F   F   F
 0.7500000000000000  0.5000000000000000  0.7244999999999990   F   F   F
 0.2272522128945156  0.1471685978434495  0.8956147538816267   T   T   T
 0.5000000000000000  0.6729049628522575  0.8934961490153590   T   T   T
 0.7727477871054842  0.1471685978434495  0.8956147538816267   T   T   T
 0.0000000000000000  0.6968512627594815  0.8950075792885543   T   T   T
 0.0000000000000000 -0.0008746449325843  0.0617313860532923   T   T   T
 0.0000000000000000 -0.0005315860961372  0.1080988912621496   T   T   T
Pt Surface(111)-4 layers + Alloys + Adso
   1.00000000000000
     5.6235999999999997    0.0000000000000000    0.0000000000000000
     0.0000000000000000    4.8701999999999996    0.0000000000000000
     0.0000000000000000    0.0000000000000000   25.0000000000000000
   Pt   Ni   C    O
    12     4     1     1
Selective dynamics
Direct
 0.0000000000000000  0.0011215113506488 -0.0137044496526976   T   T   T
 0.5000000000000000  0.0023642636386637 -0.0263023642115153   T   T   T
 0.2534250002976244  0.4991052097059889 -0.0261353352514518   T   T   T
 0.7465749997023753  0.4991052097059889 -0.0261353352514518   T   T   T
 0.0000000000000000  0.3333333333333357  0.8163320000000027   F   F   F
 0.2500000000000000  0.8333333333333357  0.8163320000000027   F   F   F
 0.5000000000000000  0.3333333333333357  0.8163320000000027   F   F   F
 0.7500000000000000  0.8333333333333357  0.8163320000000027   F   F   F
 0.0000000000000000  0.0000000000000000  0.7244999999999990   F   F   F
 0.5000000000000000  0.0000000000000000  0.7244999999999990   F   F   F
 0.2500000000000000  0.5000000000000000  0.7244999999999990   F   F   F
 0.7500000000000000  0.5000000000000000  0.7244999999999990   F   F   F
 0.2318195491499598  0.1554267150435796  0.8963935762956585   T   T   T
 0.5000000000000000  0.6669736602461989  0.8946911597072328   T   T   T
 0.7681804508500404  0.1554267150435796  0.8963935762956585   T   T   T
 0.0000000000000000  0.6878491959807130  0.8963030110970515   T   T   T
 0.0000000000000000 -0.0003197126045032  0.0611709020405239   T   T   T
 0.0000000000000000 -0.0004771411929064  0.1075091715487408   T   T   T
Pt Surface(111)-4 layers + Alloys + Adso
   1.00000000000000
     5.6235999999999997    0.0000000000000000    0.0000000000000000
     0.0000000000000000    4.8701999999999996    0.0000000000000000
     0.0000000000000000    0.0000000000000000   25.0000000000000000
   Pt   Cu   C    O
    12     4     1     1
Selective dynamics
Direct
 0.0000000000000000  0.0016697686856290 -0.0090396367837313   T   T   T
 0.5000000000000000  0.0021296161552183 -0.0188274828258667   T   T   T
 0.2557604040207626  0.5014777286542259 -0.0177631212768099   T   T   T
 0.7442395959792375  0.5014777286542259 -0.0177631212768099   T   T   T
 0.0000000000000000  0.3333333333333357  0.8163320000000027   F   F   F
 0.2500000000000000  0.8333333333333357  0.8163320000000027   F   F   F
 0.5000000000000000  0.3333333333333357  0.8163320000000027   F   F   F
 0.7500000000000000  0.8333333333333357  0.8163320000000027   F   F   F
 0.0000000000000000  0.0000000000000000  0.7244999999999990   F   F   F
 0.5000000000000000  0.0000000000000000  0.7244999999999990   F   F   F
 0.2500000000000000  0.5000000000000000  0.7244999999999990   F   F   F
 0.7500000000000000  0.5000000000000000  0.7244999999999990   F   F   F
 0.2385054603944168  0.1613515330399528  0.9003544944187274   T   T   T
 0.5000000000000000  0.6660651074518471  0.8989309888057277   T   T   T
 0.7614945396055832  0.1613515330399528  0.9003544944187274   T   T   T
 0.0000000000000000  0.6790606231882924  0.9008898028662321   T   T   T
 0.0000000000000000  0.0001621314009545  0.0658000922899581   T   T   T
 0.0000000000000000 -0.0003712999260370  0.1121382255640343   T   T   T
Pt Surface(111)-4 layers + Alloys + Adso
   1.00000000000000
     5.6235999999999997    0.0000000000000000    0.0000000000000000
     0.0000000000000000    4.8701999999999996    0.0000000000000000
     0.0000000000000000    0.0000000000000000   25.0000000000000000
   Pt   Zn   C    O
    12     4     1     1
Selective dynamics
Direct
-0.0000000000000000  0.0006781234097417 -0.0089561547190651   T   T   T
 0.5000000000000000  0.0008851171898012 -0.0190747972256312   T   T   T
 0.2539443194547960  0.5003822343236836 -0.0184481239875220   T   T   T
 0.7460556805452042  0.5003822343236836 -0.0184481239875220   T   T   T
 0.0000000000000000  0.3333333333333357  0.8163320000000027   F   F   F
 0.2500000000000000  0.8333333333333357  0.8163320000000027   F   F   F
 0.5000000000000000  0.3333333333333357  0.8163320000000027   F   F   F
 0.7500000000000000  0.8333333333333357  0.8163320000000027   F   F   F
 0.0000000000000000  0.0000000000000000  0.7244999999999990   F   F   F
 0.5000000000000000  0.0000000000000000  0.7244999999999990   F   F   F
 0.2500000000000000  0.5000000000000000  0.7244999999999990   F   F   F
 0.7500000000000000  0.5000000000000000  0.7244999999999990   F   F   F
 0.2416374691201899  0.1627734955514346  0.8999765616025770   T   T   T
 0.5000000000000000  0.6647259156804014  0.8988814875024930   T   T   T
 0.7583625308798099  0.1627734955514346  0.8999765616025770   T   T   T
-0.0000000000000000  0.6783667245358024  0.9004173235439467   T   T   T
-0.0000000000000000  0.0007147700779760  0.0674709102873195   T   T   T
-0.0000000000000000 -0.0002139271696430  0.1137763749966576   T   T   T
Pt Surface(111)-4 layers + Alloys + Adso
   1.00000000000000
     5.6235999999999997    0.0000000000000000    0.0000000000000000
     0.0000000000000000    4.8701999999999996    0.0000000000000000
     0.0000000000000000    0.0000000000000000   25.0000000000000000
   Pt   Nb   C    O
    12     4     1     1
Selective dynamics
Direct
-0.0000000000000000  0.0000189143797284  0.0047198372147236   T   T   T
 0.5000000000000000 -0.0002406825408373 -0.0033234201099606   T   T   T
 0.2531380440329022  0.5001619019161070 -0.0034232122101022   T   T   T
 0.7468619559670977  0.5001619019161070 -0.0034232122101022   T   T   T
 0.0000000000000000  0.3333333333333357  0.8163320000000027   F   F   F
 0.2500000000000000  0.8333333333333357  0.8163320000000027   F   F   F
 0.5000000000000000  0.3333333333333357  0.8163320000000027   F   F   F
 0.7500000000000000  0.8333333333333357  0.8163320000000027   F   F   F
 0.0000000000000000  0.0000000000000000  0.7244999999999990   F   F   F
 0.5000000000000000  0.0000000000000000  0.7244999999999990   F   F   F
 0.2500000000000000  0.5000000000000000  0.7244999999999990   F   F   F
 0.7500000000000000  0.5000000000000000  0.7244999999999990   F   F   F
 0.2485423388625600  0.1667933406513624  0.9065719150950882   T   T   T
 0.5000000000000000  0.6653990909771413  0.9063057409677774   T   T   T
 0.7514576611374402  0.1667933406513624  0.9065719150950882   T   T   T
-0.0000000000000000  0.6688775938489619  0.9059171276355129   T   T   T
-0.0000000000000000 -0.0000720815102772  0.0823579114080818   T   T   T
-0.0000000000000000  0.0003891945529947  0.1286816956877788   T   T   T
Pt Surface(111)-4 layers + Alloys + Adso
   1.00000000000000
     5.6235999999999997    0.0000000000000000    0.0000000000000000
     0.0000000000000000    4.8701999999999996    0.0000000000000000
     0.0000000000000000    0.0000000000000000   25.0000000000000000
   Pt   Mo   C    O
    12     4     1     1
Selective dynamics
Direct
 0.0000000000000000  0.0019195674648458  0.0011581896694238   T   T   T
 0.5000000000000000 -0.0005019749752998 -0.0087834166545062   T   T   T
 0.2528895012645853  0.5009666022879714 -0.0083310401304134   T   T   T
 0.7471104987354145  0.5009666022879714 -0.0083310401304134   T   T   T
 0.0000000000000000  0.3333333333333357  0.8163320000000027   F   F   F
 0.2500000000000000  0.8333333333333357  0.8163320000000027   F   F   F
 0.5000000000000000  0.3333333333333357  0.8163320000000027   F   F   F
 0.7500000000000000  0.8333333333333357  0.8163320000000027   F   F   F
 0.0000000000000000  0.0000000000000000  0.7244999999999990   F   F   F
 0.5000000000000000  0.0000000000000000  0.7244999999999990   F   F   F
 0.2500000000000000  0.5000000000000000  0.7244999999999990   F   F   F
 0.7500000000000000  0.5000000000000000  0.7244999999999990   F   F   F
 0.2442931861890452  0.1646315588824269  0.9033541786694473   T   T   T
 0.5000000000000000  0.6618177057286342  0.9033430351278178   T   T   T
 0.7557068138109549  0.1646315588824269  0.9033541786694473   T   T   T
 0.0000000000000000  0.6733562782113134  0.9031969454843897   T   T   T
 0.0000000000000000  0.0004788957753518  0.0778714533864301   T   T   T
 0.0000000000000000 -0.0002504078890586  0.1241956938464667   T   T   T
Pt Surface(111)-4 layers + Alloys + Adso
   1.00000000000000
     5.6235999999999997    0.0000000000000000    0.0000000000000000
     0.0000000000000000    4.8701999999999996    0.0000000000000000
     0.0000000000000000    0.0000000000000000   25.0000000000000000
   Pt   Tc   C    O
    12     4     1     1
Selective dynamics
Direct
-0.0000000000000000  0.0001803589100521 -0.0012534340664758   T   T   T
 0.5000000000000000  0.0014949418227522 -0.0112607323963671   T   T   T
 0.2529260618905745  0.4997476500306421 -0.0112200750685909   T   T   T
 0.7470739381094258  0.4997476500306421 -0.0112200750685909   T   T   T
 0.0000000000000000  0.3333333333333357  0.8163320000000027   F   F   F
 0.2500000000000000  0.8333333333333357  0.8163320000000027   F   F   F
 0.5000000000000000  0.3333333333333357  0.8163320000000027   F   F   F
 0.7500000000000000  0.8333333333333357  0.8163320000000027   F   F   F
 0.0000000000000000  0.0000000000000000  0.7244999999999990   F   F   F
 0.5000000000000000  0.0000000000000000  0.7244999999999990   F   F   F
 0.2500000000000000  0.5000000000000000  0.7244999999999990   F   F   F
 0.7500000000000000  0.5000000000000000  0.7244999999999990   F   F   F
 0.2411280855535547  0.1565341189139386  0.9017872583131826   T   T   T
 0.5000000000000000  0.6724745967381769  0.9012080126223154   T   T   T
 0.7588719144464449  0.1565341189139386  0.9017872583131826   T   T   T
-0.0000000000000000  0.6794216060024153  0.9014524351279497   T   T   T
-0.0000000000000000 -0.0004527708925633  0.0748217942730488   T   T   T
-0.0000000000000000 -0.0002003261347626  0.1211455503352753   T   T   T
Pt Surface(111)-4 layers + Alloys + Adso
   1.00000000000000
     5.6235999999999997    0.0000000000000000    0.0000000000000000
     0.0000000000000000    4.8701999999999996    0.0000000000000000
     0.0000000000000000    0.0000000000000000   25.0000000000000000
   Pt   Ru   C    O
    12     4     1     1
Selective dynamics
Direct
-0.0000000000000000  0.0008555752998841 -0.0008407619445324   T   T   T
 0.5000000000000000  0.0030973968430449 -0.0122020094285820   T   T   T
 0.2522650692211345  0.4999120864666433 -0.0121284639760776   T   T   T
 0.7477349307788649  0.4999120864666433 -0.0121284639760776   T   T   T
 0.0000000000000000  0.3333333333333357  0.8163320000000027   F   F   F
 0.2500000000000000  0.8333333333333357  0.8163320000000027   F   F   F
 0.5000000000000000  0.3333333333333357  0.8163320000000027   F   F   F
 0.7500000000000000  0.8333333333333357  0.8163320000000027   F   F   F
 0.0000000000000000  0.0000000000000000  0.7244999999999990   F   F   F
 0.5000000000000000  0.0000000000000000  0.7244999999999990   F   F   F
 0.2500000000000000  0.5000000000000000  0.7244999999999990   F   F   F
 0.7500000000000000  0.5000000000000000  0.7244999999999990   F   F   F
 0.2399089903513723  0.1578892225373341  0.9021812648469383   T   T   T
 0.5000000000000000  0.6713147332218670  0.9009842089265639   T   T   T
 0.7600910096486290  0.1578892225373341  0.9021812648469383   T   T   T
-0.0000000000000000  0.6738290300498688  0.9008787851099780   T   T   T
-0.0000000000000000 -0.0019493044896954  0.0746026841355934   T   T   T
-0.0000000000000000 -0.0008271388701111  0.1209539633299766   T   T   T
Pt Surface(111)-4 layers + Alloys + Adso
   1.00000000000000
     5.6235999999999997    0.0000000000000000    0.0000000000000000
     0.0000000000000000    4.8701999999999996    0.0000000000000000
     0.0000000000000000    0.0000000000000000   25.0000000000000000
   Pt   Rh   C    O
    12     4     1     1
Selective dynamics
Direct
 0.0000000000000000  0.0009460232532627  0.0008693574892602   T   T   T
 0.5000000000000000  0.0014698202201068 -0.0097105352620162   T   T   T
 0.2531575389447512  0.4991214327423425 -0.0096497954488661   T   T   T
 0.7468424610552488  0.4991214327423425 -0.0096497954488661   T   T   T
 0.0000000000000000  0.3333333333333357  0.8163320000000027   F   F   F
 0.2500000000000000  0.8333333333333357  0.8163320000000027   F   F   F
 0.5000000000000000  0.3333333333333357  0.8163320000000027   F   F   F
 0.7500000000000000  0.8333333333333357  0.8163320000000027   F   F   F
 0.0000000000000000  0.0000000000000000  0.7244999999999990   F   F   F
 0.5000000000000000  0.0000000000000000  0.7244999999999990   F   F   F
 0.2500000000000000  0.5000000000000000  0.7244999999999990   F   F   F
 0.7500000000000000  0.5000000000000000  0.7244999999999990   F   F   F
 0.2432601692241197  0.1627661832553299  0.9040058859650233   T   T   T
 0.5000000000000000  0.6630751768689606  0.9025284309989898   T   T   T
 0.7567398307758805  0.1627661832553299  0.9040058859650233   T   T   T
 0.0000000000000000  0.6774487405014870  0.9039498557498623   T   T   T
 0.0000000000000000 -0.0000170695062457  0.0755551005356674   T   T   T
 0.0000000000000000 -0.0003204859983562  0.1218912493275174   T   T   T
Pt NSAs with 3d elements
   1.00000000000000
     5.6235200000000001    0.0000000000000000    0.0000000000000000
     2.8117600000000000    4.8701100000000004    0.0000000000000000
     0.0000000000000000    0.0000000000000000   25.0000000000000000
   Pt   Pd   C    O
    12     4     1     1
Selective dynamics
Direct
-0.0000000000000000 -0.0000000000000000  0.0047464728261077   T   T   T
 0.4997186705148275  0.0005626589703434 -0.0030564586941842   T   T   T
 1.0005621456349181  0.4997196971856861 -0.0030564586941842   T   T   T
 0.4997181571793983  0.4997196971856861 -0.0030564586941842   T   T   T
 0.8333333333333357  0.3333333333333357  0.8163363999999973   F   F   F
 0.3333333333333357  0.3333333333333357  0.8163363999999973   F   F   F
 0.8333328199979064  0.8333343600041871  0.8163363999999973   F   F   F
 0.3333328199979064  0.8333343600041871  0.8163363999999973   F   F   F
 0.0000000000000000  0.0000000000000000  0.7245047999999983   F   F   F
 0.5000000000000000  0.0000000000000000  0.7245047999999983   F   F   F
 0.9999994866645707  0.5000010266708586  0.7245047999999983   F   F   F
 0.4999994866645707  0.5000010266708586  0.7245047999999983   F   F   F
 0.1645198251883946  0.1645213651946754  0.9074225307715016   T   T   T
 0.6709588096169339  0.1645213651946754  0.9074225307715016   T   T   T
 0.1645203385238168  0.6709593229523561  0.9074225307715016   T   T   T
 0.6666666666666643  0.6666666666666643  0.9062279368520352   T   T   T
-0.0000000000000000 -0.0000000000000000  0.0787886514678245   T   T   T
-0.0000000000000000 -0.0000000000000000  0.1251094281171380   T   T   T
Pt Surface(111)-4 layers + Alloys + Adso
   1.00000000000000
     5.6235999999999997    0.0000000000000000    0.0000000000000000
     0.0000000000000000    4.8701999999999996    0.0000000000000000
     0.0000000000000000    0.0000000000000000   25.0000000000000000
   Pt   Ag   C    O
    12     4     1     1
Selective dynamics
Direct
-0.0000000000000000  0.0001980014957209  0.0137478365612711   T   T   T
 0.5000000000000000 -0.0001224357107446  0.0062081203930409   T   T   T
 0.2568647591155799  0.5008258139891870  0.0071239875998120   T   T   T
 0.7431352408844198  0.5008258139891870  0.0071239875998120   T   T   T
 0.0000000000000000  0.3333333333333357  0.8163320000000027   F   F   F
 0.2500000000000000  0.8333333333333357  0.8163320000000027   F   F   F
 0.5000000000000000  0.3333333333333357  0.8163320000000027   F   F   F
 0.7500000000000000  0.8333333333333357  0.8163320000000027   F   F   F
 0.0000000000000000  0.0000000000000000  0.7244999999999990   F   F   F
 0.5000000000000000  0.0000000000000000  0.7244999999999990   F   F   F
 0.2500000000000000  0.5000000000000000  0.7244999999999990   F   F   F
 0.7500000000000000  0.5000000000000000  0.7244999999999990   F   F   F
 0.2473676959967564  0.1661455822094277  0.9125364716049236   T   T   T
 0.5000000000000000  0.6656644419407343  0.9112953971573173   T   T   T
 0.7526323040032435  0.1661455822094277  0.9125364716049236   T   T   T
-0.0000000000000000  0.6691549317559883  0.9132196779295014   T   T   T
-0.0000000000000000  0.0004261244363364  0.0881295091204733   T   T   T
-0.0000000000000000  0.0001017449590840  0.1344253288420174   T   T   T
Pt Surface(111)-4 layers + Alloys + Adso
   1.00000000000000
     5.6235999999999997    0.0000000000000000    0.0000000000000000
     0.0000000000000000    4.8701999999999996    0.0000000000000000
     0.0000000000000000    0.0000000000000000   25.0000000000000000
   Pt   Cd   C    O
    12     4     1     1
Selective dynamics
Direct
 0.0000000000000000  0.0013845788130361  0.0132467771980985   T   T   T
 0.5000000000000000 -0.0007970917315703  0.0044216918400543   T   T   T
 0.2550791263558368  0.5004528973501039  0.0052780776418557   T   T   T
 0.7449208736441630  0.5004528973501039  0.0052780776418557   T   T   T
 0.0000000000000000  0.3333333333333357  0.8163320000000027   F   F   F
 0.2500000000000000  0.8333333333333357  0.8163320000000027   F   F   F
 0.5000000000000000  0.3333333333333357  0.8163320000000027   F   F   F
 0.7500000000000000  0.8333333333333357  0.8163320000000027   F   F   F
 0.0000000000000000  0.0000000000000000  0.7244999999999990   F   F   F
 0.5000000000000000  0.0000000000000000  0.7244999999999990   F   F   F
 0.2500000000000000  0.5000000000000000  0.7244999999999990   F   F   F
 0.7500000000000000  0.5000000000000000  0.7244999999999990   F   F   F
 0.2470512446826751  0.1650955693339122  0.9112339647784707   T   T   T
 0.5000000000000000  0.6659447177164333  0.9104396190783391   T   T   T
 0.7529487553173252  0.1650955693339122  0.9112339647784707   T   T   T
 0.0000000000000000  0.6699912565402477  0.9123003143399424   T   T   T
 0.0000000000000000  0.0017020621542840  0.0887501193691434   T   T   T
 0.0000000000000000 -0.0002194040686745  0.1350504575769855   T   T   T
Pt Surface(111)-4 layers + Alloys + Adso
   1.00000000000000
     5.6235999999999997    0.0000000000000000    0.0000000000000000
     0.0000000000000000    4.8701999999999996    0.0000000000000000
     0.0000000000000000    0.0000000000000000   25.0000000000000000
   Pt   Ta   C    O
    12     4     1     1
Selective dynamics
Direct
-0.0000000000000000  0.0004547233046917  0.0038851881760683   T   T   T
 0.5000000000000000 -0.0004051056458891 -0.0047757300251051   T   T   T
 0.2529765012657658  0.5004042956940193 -0.0047214228185229   T   T   T
 0.7470234987342341  0.5004042956940193 -0.0047214228185229   T   T   T
 0.0000000000000000  0.3333333333333357  0.8163320000000027   F   F   F
 0.2500000000000000  0.8333333333333357  0.8163320000000027   F   F   F
 0.5000000000000000  0.3333333333333357  0.8163320000000027   F   F   F
 0.7500000000000000  0.8333333333333357  0.8163320000000027   F   F   F
 0.0000000000000000  0.0000000000000000  0.7244999999999990   F   F   F
 0.5000000000000000  0.0000000000000000  0.7244999999999990   F   F   F
 0.2500000000000000  0.5000000000000000  0.7244999999999990   F   F   F
 0.7500000000000000  0.5000000000000000  0.7244999999999990   F   F   F
 0.2472915330858646  0.1669163940760379  0.9059580770873402   T   T   T
 0.5000000000000000  0.6663671627387093  0.9058480457989719   T   T   T
 0.7527084669141356  0.1669163940760379  0.9059580770873402   T   T   T
-0.0000000000000000  0.6677410415606733  0.9056821849585607   T   T   T
-0.0000000000000000  0.0000587801418791  0.0813942937697913   T   T   T
-0.0000000000000000  0.0001945285129308  0.1277298865171739   T   T   T
Pt Surface(111)-4 layers + Alloys + Adso
   1.00000000000000
     5.6235999999999997    0.0000000000000000    0.0000000000000000
     0.0000000000000000    4.8701999999999996    0.0000000000000000
     0.0000000000000000    0.0000000000000000   25.0000000000000000
   Pt   W    C    O
    12     4     1     1
Selective dynamics
Direct
-0.0000000000000000  0.0022403751022055  0.0004832695799671   T   T   T
 0.5000000000000000 -0.0004258378943136 -0.0084828688457344   T   T   T
 0.2527018154092778  0.5013711816550066 -0.0081309319582005   T   T   T
 0.7472981845907219  0.5013711816550066 -0.0081309319582005   T   T   T
 0.0000000000000000  0.3333333333333357  0.8163320000000027   F   F   F
 0.2500000000000000  0.8333333333333357  0.8163320000000027   F   F   F
 0.5000000000000000  0.3333333333333357  0.8163320000000027   F   F   F
 0.7500000000000000  0.8333333333333357  0.8163320000000027   F   F   F
 0.0000000000000000  0.0000000000000000  0.7244999999999990   F   F   F
 0.5000000000000000  0.0000000000000000  0.7244999999999990   F   F   F
 0.2500000000000000  0.5000000000000000  0.7244999999999990   F   F   F
 0.7500000000000000  0.5000000000000000  0.7244999999999990   F   F   F
 0.2474516148465340  0.1655108855847999  0.9036326529145394   T   T   T
 0.5000000000000000  0.6616191602393765  0.9038438538128453   T   T   T
 0.7525483851534658  0.1655108855847999  0.9036326529145394   T   T   T
-0.0000000000000000  0.6724177997569269  0.9034367113770326   T   T   T
-0.0000000000000000  0.0006228531814853  0.0772179411888598   T   T   T
```

```
 -0.0000000000000000 -0.0004608195777837  0.1235561530003334   T   T   T
Pt Surface(111)-4 layers + Alloys + Adso
   1.00000000000000
     5.6235999999999997    0.0000000000000000    0.0000000000000000
     0.0000000000000000    4.8701999999999996    0.0000000000000000
     0.0000000000000000    0.0000000000000000   25.0000000000000000
   Pt   Re   C    O
    12     4     1     1
Selective dynamics
Direct
 -0.0000000000000000  0.0016804443247737 -0.0009106361226424   T   T   T
  0.5000000000000000  0.0008067533928055 -0.0104922247319787   T   T   T
  0.2524521782898676  0.5009165058370286 -0.0103154147311085   T   T   T
  0.7475478217101327  0.5009165058370286 -0.0103154147311085   T   T   T
  0.0000000000000000  0.3333333333333357  0.8163320000000027   F   F   F
  0.2500000000000000  0.8333333333333357  0.8163320000000027   F   F   F
  0.5000000000000000  0.3333333333333357  0.8163320000000027   F   F   F
  0.7500000000000000  0.8333333333333357  0.8163320000000027   F   F   F
  0.0000000000000000  0.0000000000000000  0.7244999999999990   F   F   F
  0.5000000000000000  0.0000000000000000  0.7244999999999990   F   F   F
  0.2500000000000000  0.5000000000000000  0.7244999999999990   F   F   F
  0.7500000000000000  0.5000000000000000  0.7244999999999990   F   F   F
  0.2437510626933891  0.1613511357409566  0.9020646310683389   T   T   T
  0.5000000000000000  0.6665507630452984  0.9018156297080971   T   T   T
  0.7562489373066109  0.1613511357409566  0.9020646310683389   T   T   T
 -0.0000000000000000  0.6768362987614563  0.9020409465929579   T   T   T
 -0.0000000000000000  0.0006254366838026  0.0750855017513149   T   T   T
 -0.0000000000000000 -0.0004599518565863  0.1214008549993865   T   T   T
Pt Surface(111)-4 layers + Alloys + Adso
   1.00000000000000
     5.6235999999999997    0.0000000000000000    0.0000000000000000
     0.0000000000000000    4.8701999999999996    0.0000000000000000
     0.0000000000000000    0.0000000000000000   25.0000000000000000
   Pt   Os   C    O
    12     4     1     1
Selective dynamics
Direct
  0.0000000000000000  0.0016680599700920 -0.0009741144067184   T   T   T
  0.5000000000000000  0.0056689064403786 -0.0118172569003889   T   T   T
  0.2521997568454228  0.5010726963946134 -0.0117891623656252   T   T   T
  0.7478002431545770  0.5010726963946134 -0.0117891623656252   T   T   T
  0.0000000000000000  0.3333333333333357  0.8163320000000027   F   F   F
  0.2500000000000000  0.8333333333333357  0.8163320000000027   F   F   F
  0.5000000000000000  0.3333333333333357  0.8163320000000027   F   F   F
  0.7500000000000000  0.8333333333333357  0.8163320000000027   F   F   F
  0.0000000000000000  0.0000000000000000  0.7244999999999990   F   F   F
  0.5000000000000000  0.0000000000000000  0.7244999999999990   F   F   F
  0.2500000000000000  0.5000000000000000  0.7244999999999990   F   F   F
  0.7500000000000000  0.5000000000000000  0.7244999999999990   F   F   F
  0.2377651328219045  0.1564937670209277  0.9023401012933904   T   T   T
  0.5000000000000000  0.6729749603327453  0.9014616468154253   T   T   T
  0.7622348671780954  0.1564937670209277  0.9023401012933904   T   T   T
  0.0000000000000000  0.6737175759444606  0.9008849973519140   T   T   T
  0.0000000000000000 -0.0026445547321808  0.0743954853640665   T   T   T
  0.0000000000000000 -0.0013992605198782  0.1207494616836774   T   T   T
Pt Surface(111)-4 layers + Alloys + Adso
   1.00000000000000
     5.6235999999999997    0.0000000000000000    0.0000000000000000
     0.0000000000000000    4.8701999999999996    0.0000000000000000
     0.0000000000000000    0.0000000000000000   25.0000000000000000
   Pt   Ir   C    O
    12     4     1     1
Selective dynamics
Direct
  0.0000000000000000  0.0011813038288872  0.0017438131498452   T   T   T
  0.5000000000000000  0.0025766858460634 -0.0090585986924180   T   T   T
  0.2526248462305550  0.4995507145519375 -0.0093238333568313   T   T   T
  0.7473751537694451  0.4995507145519375 -0.0093238333568313   T   T   T
  0.0000000000000000  0.3333333333333357  0.8163320000000027   F   F   F
  0.2500000000000000  0.8333333333333357  0.8163320000000027   F   F   F
  0.5000000000000000  0.3333333333333357  0.8163320000000027   F   F   F
  0.7500000000000000  0.8333333333333357  0.8163320000000027   F   F   F
  0.0000000000000000  0.0000000000000000  0.7244999999999990   F   F   F
  0.5000000000000000  0.0000000000000000  0.7244999999999990   F   F   F
  0.2500000000000000  0.5000000000000000  0.7244999999999990   F   F   F
  0.7500000000000000  0.5000000000000000  0.7244999999999990   F   F   F
  0.2438341742632540  0.1621671825821238  0.9041601335814757   T   T   T
  0.5000000000000000  0.6657110129952879  0.9028463391448464   T   T   T
  0.7561658257367460  0.1621671825821238  0.9041601335814757   T   T   T
  0.0000000000000000  0.6745532527271422  0.9038604075465260   T   T   T
  0.0000000000000000 -0.0005916388176866  0.0763612263268903   T   T   T
  0.0000000000000000 -0.0006379316463239  0.1227200404355743   T   T   T
Pt Surface(111)-4 layers + Alloys + Adso
   1.00000000000000
     5.6235999999999997    0.0000000000000000    0.0000000000000000
     0.0000000000000000    4.8701999999999996    0.0000000000000000
     0.0000000000000000    0.0000000000000000   25.0000000000000000
   Pt   C    O
    16     1     1
Selective dynamics
Direct
  0.0000000000000000  0.0019557771269832  0.0063075507920591   T   T   T
  0.5000000000000000  0.0001109585499781 -0.0025959313012718   T   T   T
  0.2559575982232182  0.5006399009003043 -0.0021448313827504   T   T   T
  0.7440424017767817  0.5006399009003043 -0.0021448313827504   T   T   T
  0.0000000000000000  0.3333333333333357  0.8163320000000027   F   F   F
  0.2500000000000000  0.8333333333333357  0.8163320000000027   F   F   F
  0.5000000000000000  0.3333333333333357  0.8163320000000027   F   F   F
  0.7500000000000000  0.8333333333333357  0.8163320000000027   F   F   F
  0.0000000000000000  0.0000000000000000  0.7244999999999990   F   F   F
  0.5000000000000000  0.0000000000000000  0.7244999999999990   F   F   F
  0.2500000000000000  0.5000000000000000  0.7244999999999990   F   F   F
  0.7500000000000000  0.5000000000000000  0.7244999999999990   F   F   F
  0.2458149755970688  0.1650537802263554  0.9077546143592006   T   T   T
  0.5000000000000000  0.6633447561273740  0.9059267128207243   T   T   T
  0.7541850244029311  0.1650537802263554  0.9077546143592006   T   T   T
  0.0000000000000000  0.6734560380891204  0.9084721856230636   T   T   T
  0.0000000000000000  0.0010446670533628  0.0802694317239871   T   T   T
  0.0000000000000000 -0.0003726070759310  0.1266189668844333   T   T   T
Pt Surface(111)-4 layers + Alloys + Adso
   1.00000000000000
     5.6235999999999997    0.0000000000000000    0.0000000000000000
     0.0000000000000000    4.8701999999999996    0.0000000000000000
     0.0000000000000000    0.0000000000000000   25.0000000000000000
   Pt   Au   C    O
    12     4     1     1
Selective dynamics
Direct
 -0.0000000000000000  0.0019081101718963  0.0142447506385927   T   T   T
  0.5000000000000000  0.0007117578331176  0.0074215353895381   T   T   T
  0.2576505968487798  0.5023801686493613  0.0087850327800922   T   T   T
  0.7423494031512202  0.5023801686493613  0.0087850327800922   T   T   T
  0.0000000000000000  0.3333333333333357  0.8163320000000027   F   F   F
  0.2500000000000000  0.8333333333333357  0.8163320000000027   F   F   F
  0.5000000000000000  0.3333333333333357  0.8163320000000027   F   F   F
  0.7500000000000000  0.8333333333333357  0.8163320000000027   F   F   F
  0.0000000000000000  0.0000000000000000  0.7244999999999990   F   F   F
  0.5000000000000000  0.0000000000000000  0.7244999999999990   F   F   F
  0.2500000000000000  0.5000000000000000  0.7244999999999990   F   F   F
  0.7500000000000000  0.5000000000000000  0.7244999999999990   F   F   F
  0.2475078235879981  0.1658901324693082  0.9129309753881112   T   T   T
  0.5000000000000000  0.6654189467302205  0.9112791430033036   T   T   T
  0.7524921764120018  0.1658901324693082  0.9129309753881112   T   T   T
 -0.0000000000000000  0.6690949309939891  0.9142917190652698   T   T   T
 -0.0000000000000000  0.0010764609998948  0.0883131922057424   T   T   T
 -0.0000000000000000  0.0005391482545719  0.1346205008360083   T   T   T
Pt Surface(111)-4 layers + Alloys + Adso
   1.00000000000000
     5.6235999999999997    0.0000000000000000    0.0000000000000000
     0.0000000000000000    4.8701999999999996    0.0000000000000000
     0.0000000000000000    0.0000000000000000   25.0000000000000000
   Pt   Hg   C    O
    12     4     1     1
Selective dynamics
Direct
 -0.0000000000000000  0.0023130814090542  0.0225743276173772   T   T   T
  0.5000000000000000  0.0011240390793566  0.0128042952073887   T   T   T
  0.2565426754299795  0.5019558378263732  0.0142826959582872   T   T   T
  0.7434573245700203  0.5019558378263732  0.0142826959582872   T   T   T
  0.0000000000000000  0.3333333333333357  0.8163320000000027   F   F   F
  0.2500000000000000  0.8333333333333357  0.8163320000000027   F   F   F
  0.5000000000000000  0.3333333333333357  0.8163320000000027   F   F   F
  0.7500000000000000  0.8333333333333357  0.8163320000000027   F   F   F
  0.0000000000000000  0.0000000000000000  0.7244999999999990   F   F   F
  0.5000000000000000  0.0000000000000000  0.7244999999999990   F   F   F
  0.2500000000000000  0.5000000000000000  0.7244999999999990   F   F   F
  0.7500000000000000  0.5000000000000000  0.7244999999999990   F   F   F
  0.2479655404208474  0.1653122182988342  0.9152767798300609   T   T   T
  0.5000000000000000  0.6644106734810187  0.9134916436968969   T   T   T
  0.7520344595791528  0.1653122182988342  0.9152767798300609   T   T   T
 -0.0000000000000000  0.6688581595855702  0.9178945104101739   T   T   T
 -0.0000000000000000  0.0017736131969657  0.0971579559707609   T   T   T
 -0.0000000000000000  0.0000904414985011  0.1434587483288869   T   T   T
```

111 *OH

```
Pt Surface(111)-4 layers + Alloys + Adso
   1.00000000000000
     5.6235999999999997    0.0000000000000000    0.0000000000000000
     0.0000000000000000    4.8701999999999996    0.0000000000000000
     0.0000000000000000    0.0000000000000000   25.0000000000000000
   Pt   V    O    H
    12     4     1     1
Selective dynamics
Direct
  0.0055921250875362  0.0009705346037334 -0.0153202822757731   T   T   T
  0.5037271068662794  0.0019336438366819 -0.0169391280901016   T   T   T
  0.2548109479414518  0.4996522438809729 -0.0194549987149064   T   T   T
  0.7513078590332198  0.5003196797795240 -0.0194931631624185   T   T   T
  0.0000000000000000  0.3333333333333357  0.8163320000000027   F   F   F
  0.2500000000000000  0.8333333333333357  0.8163320000000027   F   F   F
  0.5000000000000000  0.3333333333333357  0.8163320000000027   F   F   F
  0.7500000000000000  0.8333333333333357  0.8163320000000027   F   F   F
  0.0000000000000000  0.0000000000000000  0.7244999999999990   F   F   F
  0.5000000000000000  0.0000000000000000  0.7244999999999990   F   F   F
  0.2500000000000000  0.5000000000000000  0.7244999999999990   F   F   F
  0.7500000000000000  0.5000000000000000  0.7244999999999990   F   F   F
  0.2435373701519747  0.1387630978241611  0.8991649496464661   T   T   T
  0.5010354656092245  0.6890455788802586  0.8989659339846521   T   T   T
  0.7607013052278857  0.1388393950162583  0.8991006572277557   T   T   T
  0.0002549021172476  0.7033932523296929  0.8996549875693421   T   T   T
 -0.0105897617273911  0.0068324061194577  0.0673362256298591   T   T   T
  0.1363685364507010  0.9380390382182734  0.0833398827734926   T   T   T
Pt Surface(111)-4 layers + Alloys + Adso
   1.00000000000000
     5.6235999999999997    0.0000000000000000    0.0000000000000000
     0.0000000000000000    4.8701999999999996    0.0000000000000000
     0.0000000000000000    0.0000000000000000   25.0000000000000000
   Pt   Cr   O    H
    12     4     1     1
Selective dynamics
Direct
  0.0034583645543925 -0.0167155811063129 -0.0155011761097758   T   T   T
  0.4995296116042732 -0.0047226041219627 -0.0238895518878668   T   T   T
  0.2587230386165190  0.4902974608022989 -0.0253106442529689   T   T   T
  0.7498167651329830  0.4864393431679666 -0.0219151839978802   T   T   T
  0.0000000000000000  0.3333333333333357  0.8163320000000027   F   F   F
  0.2500000000000000  0.8333333333333357  0.8163320000000027   F   F   F
  0.5000000000000000  0.3333333333333357  0.8163320000000027   F   F   F
  0.7500000000000000  0.8333333333333357  0.8163320000000027   F   F   F
  0.0000000000000000  0.0000000000000000  0.7244999999999990   F   F   F
  0.5000000000000000  0.0000000000000000  0.7244999999999990   F   F   F
  0.2500000000000000  0.5000000000000000  0.7244999999999990   F   F   F
  0.7500000000000000  0.5000000000000000  0.7244999999999990   F   F   F
  0.2024327700926290  0.1322012497563888  0.8981942111035138   T   T   T
  0.5363503756082526  0.6730229768571958  0.8965557210923386   T   T   T
  0.7900420113396300  0.1345588045163130  0.8980963761426561   T   T   T
 -0.0163555195170506  0.6984214383609187  0.8979624405204601   T   T   T
 -0.0066512836673431  0.0021874461085553  0.0658640048915566   T   T   T
  0.1407804419098059  0.9361651823280177  0.0821313999158033   T   T   T
Pt NSAs with 3d elements
   1.00000000000000
     5.6235200000000001    0.0000000000000000    0.0000000000000000
     2.8117600000000000    4.8701100000000004    0.0000000000000000
     0.0000000000000000    0.0000000000000000   25.0000000000000000
   Pt   Mn   O    H
    12     4     1     1
Selective dynamics
Direct
  0.0005611478206895  0.0009922878635070 -0.0178437248893771   T   T   T
  0.4994899348866224 -0.0005482892177017 -0.0315098232630452   T   T   T
  1.0124320572445376  0.4943237189977044 -0.0253800055885669   T   T   T
  0.4963767794501638  0.5020841136584735 -0.0299303735410480   T   T   T
  0.8333333333333357  0.3333333333333357  0.8163363999999973   F   F   F
  0.3333333333333357  0.3333333333333357  0.8163363999999973   F   F   F
  0.8333328199979064  0.8333343600041871  0.8163363999999973   F   F   F
  0.3333328199979064  0.8333343600041871  0.8163363999999973   F   F   F
  0.0000000000000000  0.0000000000000000  0.7245047999999983   F   F   F
  0.5000000000000000  0.0000000000000000  0.7245047999999983   F   F   F
  0.9999994866645707  0.5000010266708586  0.7245047999999983   F   F   F
  0.4999994866645707  0.5000010266708586  0.7245047999999983   F   F   F
  0.1234540219182715  0.1596699378328772  0.8967175789227670   T   T   T
  0.7214044359965499  0.1465641533317759  0.8957413360622435   T   T   T
  0.1220546716986133  0.7221678457486833  0.8969447667623953   T   T   T
  0.7160922559675973  0.6346543675766171  0.8939384553228779   T   T   T
 -0.0156579378556752  0.0070776542266058  0.0624739198548540   T   T   T
  0.1700038292200633  0.9359239000292704  0.0765701880400808   T   T   T
Pt Surface(111)-4 layers + Alloys + Adso
   1.00000000000000
     5.6235999999999997    0.0000000000000000    0.0000000000000000
     0.0000000000000000    4.8701999999999996    0.0000000000000000
     0.0000000000000000    0.0000000000000000   25.0000000000000000
   Pt   Fe   O    H
    12     4     1     1
Selective dynamics
Direct
  0.0053434212077812 -0.0151332248445125 -0.0183851893001994   T   T   T
  0.5024672581482744  0.0053812956162966 -0.0248715353203339   T   T   T
  0.2568269769934460  0.4917538018099351 -0.0299828622197829   T   T   T
  0.7484632047175833  0.4910267338087381 -0.0297496549637701   T   T   T
  0.0000000000000000  0.3333333333333357  0.8163320000000027   F   F   F
  0.2500000000000000  0.8333333333333357  0.8163320000000027   F   F   F
  0.5000000000000000  0.3333333333333357  0.8163320000000027   F   F   F
  0.7500000000000000  0.8333333333333357  0.8163320000000027   F   F   F
  0.0000000000000000  0.0000000000000000  0.7244999999999990   F   F   F
  0.5000000000000000  0.0000000000000000  0.7244999999999990   F   F   F
  0.2500000000000000  0.5000000000000000  0.7244999999999990   F   F   F
  0.7500000000000000  0.5000000000000000  0.7244999999999990   F   F   F
  0.2216528538599166  0.1266933450847347  0.8967105256269471   T   T   T
  0.5024479254644661  0.7053079654879054  0.8941249698427635   T   T   T
  0.7759630320591656  0.1286384845078276  0.8962024161319780   T   T   T
 -0.0002333101509894  0.7083226702931728  0.8954016659079709   T   T   T
 -0.0105977587588754  0.0131360216248486  0.0618689597166561   T   T   T
  0.1350131360139807  0.9342059836783821  0.0767788065256089   T   T   T
Pt Surface(111)-4 layers + Alloys + Adso
   1.00000000000000
     5.6235999999999997    0.0000000000000000    0.0000000000000000
     0.0000000000000000    4.8701999999999996    0.0000000000000000
     0.0000000000000000    0.0000000000000000   25.0000000000000000
   Pt   Co   O    H
    12     4     1     1
Selective dynamics
Direct
  0.0063387950643490 -0.0076481570531709 -0.0198331066193730   T   T   T
  0.5037438309618841  0.0015125496524716 -0.0270440538267005   T   T   T
  0.2556654627292057  0.4968446475518096 -0.0274448175526327   T   T   T
  0.7503504257594646  0.4955622921254535 -0.0272328749387455   T   T   T
  0.0000000000000000  0.3333333333333357  0.8163320000000027   F   F   F
  0.2500000000000000  0.8333333333333357  0.8163320000000027   F   F   F
  0.5000000000000000  0.3333333333333357  0.8163320000000027   F   F   F
  0.7500000000000000  0.8333333333333357  0.8163320000000027   F   F   F
  0.0000000000000000  0.0000000000000000  0.7244999999999990   F   F   F
  0.5000000000000000  0.0000000000000000  0.7244999999999990   F   F   F
  0.2500000000000000  0.5000000000000000  0.7244999999999990   F   F   F
  0.7500000000000000  0.5000000000000000  0.7244999999999990   F   F   F
  0.2341537226835153  0.1544297455634549  0.8955188178340197   T   T   T
  0.5020590227770267  0.6696343019354544  0.8933977274033083   T   T   T
  0.7666365398657862  0.1551944927750100  0.8949641761350521   T   T   T
 -0.0008229963662650  0.6878665638399278  0.8952571939215135   T   T   T
 -0.0119005857555714  0.0104156340115469  0.0608137611798259   T   T   T
  0.1352108289409829  0.9350779696197641  0.0757298074912349   T   T   T
Pt Surface(111)-4 layers + Alloys + Adso
   1.00000000000000
     5.6235999999999997    0.0000000000000000    0.0000000000000000
     0.0000000000000000    4.8701999999999996    0.0000000000000000
     0.0000000000000000    0.0000000000000000   25.0000000000000000
   Pt   Ni   O    H
    12     4     1     1
Selective dynamics
Direct
  0.0066991577086069 -0.0060989725761442 -0.0184966812273151   T   T   T
  0.5046005167863267  0.0000233498856836 -0.0265959298251331   T   T   T
  0.2557667828581229  0.4965185460467313 -0.0264282655670149   T   T   T
  0.7518348594418287  0.4961626367089743 -0.0264942700455754   T   T   T
  0.0000000000000000  0.3333333333333357  0.8163320000000027   F   F   F
  0.2500000000000000  0.8333333333333357  0.8163320000000027   F   F   F
  0.5000000000000000  0.3333333333333357  0.8163320000000027   F   F   F
  0.7500000000000000  0.8333333333333357  0.8163320000000027   F   F   F
  0.0000000000000000  0.0000000000000000  0.7244999999999990   F   F   F
  0.5000000000000000  0.0000000000000000  0.7244999999999990   F   F   F
  0.2500000000000000  0.5000000000000000  0.7244999999999990   F   F   F
  0.7500000000000000  0.5000000000000000  0.7244999999999990   F   F   F
  0.2392141890663692  0.1589105654219824  0.8958824075418734   T   T   T
  0.5016400413103369  0.6656250717237201  0.8942960872380994   T   T   T
  0.7649867467021206  0.1580320266793474  0.8955382552567096   T   T   T
  0.0005605969470939  0.6798987565959185  0.8959114311268567   T   T   T
 -0.0100789695457554  0.0077911949273310  0.0617965405087455   T   T   T
  0.1382761006522037  0.9320436137170381  0.0759963141731876   T   T   T
Pt Surface(111)-4 layers + Alloys + Adso
   1.00000000000000
     5.6235999999999997    0.0000000000000000    0.0000000000000000
     0.0000000000000000    4.8701999999999996    0.0000000000000000
     0.0000000000000000    0.0000000000000000   25.0000000000000000
   Pt   Cu   O    H
    12     4     1     1
Selective dynamics
Direct
  0.0037344027802341 -0.0031716103459507 -0.0119370921871943   T   T   T
  0.5030404189760175  0.0008352840028381 -0.0185615249706999   T   T   T
  0.2555779273927229  0.4990822273278165 -0.0181216242151464   T   T   T
  0.7501844309068907  0.4987387396072011 -0.0184798789919727   T   T   T
  0.0000000000000000  0.3333333333333357  0.8163320000000027   F   F   F
  0.2500000000000000  0.8333333333333357  0.8163320000000027   F   F   F
  0.5000000000000000  0.3333333333333357  0.8163320000000027   F   F   F
  0.7500000000000000  0.8333333333333357  0.8163320000000027   F   F   F
  0.0000000000000000  0.0000000000000000  0.7244999999999990   F   F   F
  0.5000000000000000  0.0000000000000000  0.7244999999999990   F   F   F
  0.2500000000000000  0.5000000000000000  0.7244999999999990   F   F   F
  0.7500000000000000  0.5000000000000000  0.7244999999999990   F   F   F
  0.2416171480093252  0.1615997344707540  0.9002374968862176   T   T   T
  0.5005947892648130  0.6643644940010806  0.8991273924649449   T   T   T
  0.7603343599384490  0.1613822214516197  0.9000554214371721   T   T   T
  0.0009041447161954  0.6777894413118827  0.9004556786735362   T   T   T
 -0.0041745040608021  0.0088441837773293  0.0682422984486343   T   T   T
  0.1451872060965457  0.9291816664926282  0.0810880940965641   T   T   T
Pt Surface(111)-4 layers + Alloys + Adso
   1.00000000000000
     5.6235999999999997    0.0000000000000000    0.0000000000000000
     0.0000000000000000    4.8701999999999996    0.0000000000000000
     0.0000000000000000    0.0000000000000000   25.0000000000000000
   Pt   Zn   O    H
    12     4     1     1
Selective dynamics
Direct
  0.0052090401506386 -0.0018422824490419 -0.0141914562763652   T   T   T
  0.5043927388117385 -0.0005664353716193 -0.0181357302536562   T   T   T
  0.2564289780744170  0.4986347140287804 -0.0175907468135846   T   T   T
  0.7515843396603298  0.4986117560960225 -0.0176921848756602   T   T   T
  0.0000000000000000  0.3333333333333357  0.8163320000000027   F   F   F
  0.2500000000000000  0.8333333333333357  0.8163320000000027   F   F   F
  0.5000000000000000  0.3333333333333357  0.8163320000000027   F   F   F
  0.7500000000000000  0.8333333333333357  0.8163320000000027   F   F   F
  0.0000000000000000  0.0000000000000000  0.7244999999999990   F   F   F
  0.5000000000000000  0.0000000000000000  0.7244999999999990   F   F   F
  0.2500000000000000  0.5000000000000000  0.7244999999999990   F   F   F
  0.7500000000000000  0.5000000000000000  0.7244999999999990   F   F   F
  0.2448344643711179  0.1625756234894826  0.9001702802651025   T   T   T
  0.5014888962466596  0.6627894973268345  0.8997158868918953   T   T   T
  0.7588248655879604  0.1636641954815513  0.9001240809095555   T   T   T
  0.0011402871134671  0.6763604363719010  0.9004685816148985   T   T   T
 -0.0077427055485519  0.0084520750910505  0.0680799852064749   T   T   T
  0.1405013413521666  0.9317299078377616  0.0820615510689071   T   T   T
Pt Surface(111)-4 layers + Alloys + Adso
   1.00000000000000
     5.6235999999999997    0.0000000000000000    0.0000000000000000
     0.0000000000000000    4.8701999999999996    0.0000000000000000
     0.0000000000000000    0.0000000000000000   25.0000000000000000
   Pt   Nb   O    H
    12     4     1     1
Selective dynamics
Direct
```

```
 0.0064503484097920 -0.0042907909475854 -0.0025119267535976   T   T   T
 0.5051050757815524 -0.0020512146632742 -0.0030422627637370   T   T   T
 0.2563376095615744  0.4965917926240770 -0.0029632459346493   T   T   T
 0.7532716577988751  0.4974768868135545 -0.0030805971836184   T   T   T
 0.0000000000000000  0.3333333333333357  0.8163320000000027   F   F   F
 0.2500000000000000  0.8333333333333357  0.8163320000000027   F   F   F
 0.5000000000000000  0.3333333333333357  0.8163320000000027   F   F   F
 0.7500000000000000  0.8333333333333357  0.8163320000000027   F   F   F
 0.0000000000000000  0.0000000000000000  0.7244999999999990   F   F   F
 0.5000000000000000  0.0000000000000000  0.7244999999999990   F   F   F
 0.2500000000000000  0.5000000000000000  0.7244999999999990   F   F   F
 0.7500000000000000  0.5000000000000000  0.7244999999999990   F   F   F
 0.2501103992049943  0.1645033055754901  0.9069572981285530   T   T   T
 0.5022483609482556  0.6628776553255493  0.9068171100624307   T   T   T
 0.7548647665111887  0.1650726746201964  0.9067994971940309   T   T   T
 0.0022185228565914  0.6693111379042572  0.9064030649630562   T   T   T
-0.0119602207767297  0.0091309512184743  0.0803946321229814   T   T   T
 0.1332199874513520  0.9357883376825402  0.0964082999263170   T   T   T
Pt Surface(111)-4 layers + Alloys + Adso
   1.00000000000000
     5.6235999999999997    0.0000000000000000    0.0000000000000000
     0.0000000000000000    4.8701999999999996    0.0000000000000000
     0.0000000000000000    0.0000000000000000   25.0000000000000000
   Pt   Mo   O    H
   12     4     1     1
Selective dynamics
Direct
 0.0017848168491382  0.0013307395390992 -0.0070020840037278   T   T   T
 0.5007290099584307  0.0026890983082374 -0.0080707238049490   T   T   T
 0.2524573085392699  0.5014794332132811 -0.0081164446622319   T   T   T
 0.7471153229400088  0.5022075490980328 -0.0082924936302555   T   T   T
 0.0000000000000000  0.3333333333333357  0.8163320000000027   F   F   F
 0.2500000000000000  0.8333333333333357  0.8163320000000027   F   F   F
 0.5000000000000000  0.3333333333333357  0.8163320000000027   F   F   F
 0.7500000000000000  0.8333333333333357  0.8163320000000027   F   F   F
 0.0000000000000000  0.0000000000000000  0.7244999999999990   F   F   F
 0.5000000000000000  0.0000000000000000  0.7244999999999990   F   F   F
 0.2500000000000000  0.5000000000000000  0.7244999999999990   F   F   F
 0.7500000000000000  0.5000000000000000  0.7244999999999990   F   F   F
 0.2447930925306323  0.1640045902962875  0.9040835798846224   T   T   T
 0.4990848832650374  0.6632575536866874  0.9033602426250499   T   T   T
 0.7543452400396611  0.1652226938828373  0.9037878338750248   T   T   T
-0.0010374257598926  0.6778856966529696  0.9037563018518722   T   T   T
-0.0200529451611338  0.0395187798852574  0.0748191629641860   T   T   T
 0.1079465103903909  0.9363699148882527  0.0920575670853879   T   T   T
Pt Surface(111)-4 layers + Alloys + Adso
   1.00000000000000
     5.6235999999999997    0.0000000000000000    0.0000000000000000
     0.0000000000000000    4.8701999999999996    0.0000000000000000
     0.0000000000000000    0.0000000000000000   25.0000000000000000
   Pt   Tc   O    H
   12     4     1     1
Selective dynamics
Direct
 0.0058574205584657 -0.0069174433093849 -0.0082865170383823   T   T   T
 0.5037184099122185  0.0005092133186862 -0.0108383372384930   T   T   T
 0.2567827950017495  0.4961265190053261 -0.0111453982579078   T   T   T
 0.7488799586573744  0.4964417005282839 -0.0111433061402640   T   T   T
 0.0000000000000000  0.3333333333333357  0.8163320000000027   F   F   F
 0.2500000000000000  0.8333333333333357  0.8163320000000027   F   F   F
 0.5000000000000000  0.3333333333333357  0.8163320000000027   F   F   F
 0.7500000000000000  0.8333333333333357  0.8163320000000027   F   F   F
 0.0000000000000000  0.0000000000000000  0.7244999999999990   F   F   F
 0.5000000000000000  0.0000000000000000  0.7244999999999990   F   F   F
 0.2500000000000000  0.5000000000000000  0.7244999999999990   F   F   F
 0.7500000000000000  0.5000000000000000  0.7244999999999990   F   F   F
 0.2402596876911719  0.1546124273947070  0.9020934133940385   T   T   T
 0.4995655277977117  0.6744244822433699  0.9012789128554349   T   T   T
 0.7658285266646875  0.1550561839782998  0.9019494724821665   T   T   T
-0.0012445223488775  0.6802109957395525  0.9019037037293830   T   T   T
-0.0068302863958638  0.0113993091237276  0.0728718009743192   T   T   T
 0.1401588277770442  0.9335714553961632  0.0872601577286163   T   T   T
Pt Surface(111)-4 layers + Alloys + Adso
   1.00000000000000
     5.6235999999999997    0.0000000000000000    0.0000000000000000
     0.0000000000000000    4.8701999999999996    0.0000000000000000
     0.0000000000000000    0.0000000000000000   25.0000000000000000
   Pt   Ru   O    H
   12     4     1     1
Selective dynamics
Direct
 0.0058366081896274 -0.0068847624256205 -0.0088147331300767   T   T   T
 0.5036363136163233  0.0015674838654320 -0.0119010008863083   T   T   T
 0.2568169818296108  0.4969467639000711 -0.0120104095838817   T   T   T
 0.7495471610383896  0.4967960585420972 -0.0120176329549860   T   T   T
 0.0000000000000000  0.3333333333333357  0.8163320000000027   F   F   F
 0.2500000000000000  0.8333333333333357  0.8163320000000027   F   F   F
 0.5000000000000000  0.3333333333333357  0.8163320000000027   F   F   F
 0.7500000000000000  0.8333333333333357  0.8163320000000027   F   F   F
 0.0000000000000000  0.0000000000000000  0.7244999999999990   F   F   F
 0.5000000000000000  0.0000000000000000  0.7244999999999990   F   F   F
 0.2500000000000000  0.5000000000000000  0.7244999999999990   F   F   F
 0.7500000000000000  0.5000000000000000  0.7244999999999990   F   F   F
 0.2440206640024560  0.1604803948148588  0.9018955895291980   T   T   T
 0.5010927377905751  0.6692285654036735  0.9006219157879519   T   T   T
 0.7574318740536148  0.1612703774633072  0.9014978125023243   T   T   T
 0.0006745482928326  0.6742497687495566  0.9015091355078637   T   T   T
-0.0092761032130176  0.0089873404198167  0.0717651846355380   T   T   T
 0.1406558377791530  0.9346008069953265  0.0853791050456479   T   T   T
Pt Surface(111)-4 layers + Alloys + Adso
   1.00000000000000
     5.6235999999999997    0.0000000000000000    0.0000000000000000
     0.0000000000000000    4.8701999999999996    0.0000000000000000
     0.0000000000000000    0.0000000000000000   25.0000000000000000
   Pt   Rh   O    H
   12     4     1     1
Selective dynamics
Direct
 0.0065867007126565 -0.0075297822379529 -0.0043374540129153   T   T   T
 0.5044548757889656 -0.0010993181075189 -0.0096656639340034   T   T   T
 0.2563518100957451  0.4957564479201303 -0.0095478567285312   T   T   T
 0.7505359550927017  0.4951013077027585 -0.0095862380171099   T   T   T
 0.0000000000000000  0.3333333333333357  0.8163320000000027   F   F   F
 0.2500000000000000  0.8333333333333357  0.8163320000000027   F   F   F
 0.5000000000000000  0.3333333333333357  0.8163320000000027   F   F   F
 0.7500000000000000  0.8333333333333357  0.8163320000000027   F   F   F
 0.0000000000000000  0.0000000000000000  0.7244999999999990   F   F   F
 0.5000000000000000  0.0000000000000000  0.7244999999999990   F   F   F
 0.2500000000000000  0.5000000000000000  0.7244999999999990   F   F   F
 0.7500000000000000  0.5000000000000000  0.7244999999999990   F   F   F
 0.2462722148064358  0.1638717832151634  0.9035558728673689   T   T   T
 0.5013017422055770  0.6652152181276610  0.9021966271933451   T   T   T
 0.7551648429741721  0.1642682552587200  0.9031810718131298   T   T   T
 0.0006833181084955  0.6730255520651174  0.9038639016808084   T   T   T
-0.0067005913086243  0.0051192719274767  0.0757763550994197   T   T   T
 0.1454468339853426  0.9338716411193190  0.0888441971830390   T   T   T
Pt NSAs with 3d elements
   1.00000000000000
     5.6235200000000001    0.0000000000000000    0.0000000000000000
     2.8117600000000000    4.8701100000000004    0.0000000000000000
     0.0000000000000000    0.0000000000000000   25.0000000000000000
   Pt   Pd   O    H
   12     4     1     1
Selective dynamics
Direct
 0.0015932856035812 -0.0019091517236821  0.0028809569830055   T   T   T
 0.5002795351646485  0.0001043173597597 -0.0043606903606170   T   T   T
 1.0001871093197234  0.4991245683362157 -0.0040731496303378   T   T   T
 0.5006852910433409  0.4984606497758440 -0.0043334766081218   T   T   T
 0.8333333333333357  0.3333333333333357  0.8163363999999973   F   F   F
 0.3333333333333357  0.3333333333333357  0.8163363999999973   F   F   F
 0.8333328199979064  0.8333343600041871  0.8163363999999973   F   F   F
 0.3333328199979064  0.8333343600041871  0.8163363999999973   F   F   F
 0.0000000000000000  0.0000000000000000  0.7245047999999983   F   F   F
 0.5000000000000000  0.0000000000000000  0.7245047999999983   F   F   F
 0.9999994866645707  0.5000010266708586  0.7245047999999983   F   F   F
 0.4999994866645707  0.5000010266708586  0.7245047999999983   F   F   F
 0.1643769360693831  0.1656293304995891  0.9066972282340902   T   T   T
 0.6709930034663760  0.1647431464582235  0.9066399423788495   T   T   T
 0.1642114076755312  0.6705997849316991  0.9068804902850819   T   T   T
 0.6661070497033917  0.6670770147300842  0.9052099243330508   T   T   T
-0.0002740176029227  0.0003994193162084  0.0825380601944592   T   T   T
 0.1909247585501677  0.9306072226918815  0.0933020747498275   T   T   T
Pt Surface(111)-4 layers + Alloys + Adso
   1.00000000000000
     5.6235999999999997    0.0000000000000000    0.0000000000000000
     0.0000000000000000    4.8701999999999996    0.0000000000000000
     0.0000000000000000    0.0000000000000000   25.0000000000000000
   Pt   Ag   O    H
   12     4     1     1
Selective dynamics
Direct
-0.0016442175749266 -0.0019306319943574  0.0142004419769019   T   T   T
 0.4988736050350358  0.0009943951450132  0.0066450921298042   T   T   T
 0.2522866696371572  0.5003251200821679  0.0067963033797192   T   T   T
 0.7454467012217516  0.5006074262529440  0.0065954702914753   T   T   T
 0.0000000000000000  0.3333333333333357  0.8163320000000027   F   F   F
 0.2500000000000000  0.8333333333333357  0.8163320000000027   F   F   F
 0.5000000000000000  0.3333333333333357  0.8163320000000027   F   F   F
 0.7500000000000000  0.8333333333333357  0.8163320000000027   F   F   F
 0.0000000000000000  0.0000000000000000  0.7244999999999990   F   F   F
 0.5000000000000000  0.0000000000000000  0.7244999999999990   F   F   F
 0.2500000000000000  0.5000000000000000  0.7244999999999990   F   F   F
 0.7500000000000000  0.5000000000000000  0.7244999999999990   F   F   F
 0.2458347352603505  0.1670738509154020  0.9126934318491592   T   T   T
 0.4978624893888554  0.6671772881326424  0.9113822216344081   T   T   T
 0.7506250914772959  0.1668390458338209  0.9127171393807146   T   T   T
-0.0022018632874223  0.6710444169275122  0.9132147038599158   T   T   T
 0.0089647956994498  0.0035502701702452  0.0937787359658337   T   T   T
 0.1616246312312440  0.9229941707595878  0.1043977032606401   T   T   T
Pt Surface(111)-4 layers + Alloys + Adso
   1.00000000000000
     5.6235999999999997    0.0000000000000000    0.0000000000000000
     0.0000000000000000    4.8701999999999996    0.0000000000000000
     0.0000000000000000    0.0000000000000000   25.0000000000000000
   Pt   Cd   O    H
   12     4     1     1
Selective dynamics
Direct
 0.0013482052848123  0.0002836391817588  0.0098551136673849   T   T   T
 0.5007530026925681  0.0001507997448465  0.0060037977115384   T   T   T
 0.2534005626284309  0.4999395125425311  0.0066178229538479   T   T   T
 0.7467819866542553  0.5004194292172753  0.0065681721917942   T   T   T
 0.0000000000000000  0.3333333333333357  0.8163320000000027   F   F   F
 0.2500000000000000  0.8333333333333357  0.8163320000000027   F   F   F
 0.5000000000000000  0.3333333333333357  0.8163320000000027   F   F   F
 0.7500000000000000  0.8333333333333357  0.8163320000000027   F   F   F
 0.0000000000000000  0.0000000000000000  0.7244999999999990   F   F   F
 0.5000000000000000  0.0000000000000000  0.7244999999999990   F   F   F
 0.2500000000000000  0.5000000000000000  0.7244999999999990   F   F   F
 0.7500000000000000  0.5000000000000000  0.7244999999999990   F   F   F
 0.2466175225248962  0.1663685528415365  0.9120784708456319   T   T   T
 0.4988654596934304  0.6661487152336459  0.9115403877823453   T   T   T
 0.7515955748079173  0.1666332768891111  0.9121106574398449   T   T   T
-0.0011722160882478  0.6708683191220806  0.9128453497160293   T   T   T
 0.0028748783873474  0.0004299118290742  0.0912454146870269   T   T   T
 0.1565439403397985  0.9261045367099385  0.1026822956604781   T   T   T
Pt Surface(111)-4 layers + Alloys + Adso
   1.00000000000000
     5.6235999999999997    0.0000000000000000    0.0000000000000000
     0.0000000000000000    4.8701999999999996    0.0000000000000000
     0.0000000000000000    0.0000000000000000   25.0000000000000000
   Pt   Ta   O    H
   12     4     1     1
Selective dynamics
Direct
 0.0030894816029559 -0.0030778574956709 -0.0036697533927538   T   T   T
 0.5025487780495810 -0.0007817899631210 -0.0041712908105693   T   T   T
 0.2541572290582177  0.4975185608117417 -0.0040141135366685   T   T   T
 0.7503801878308529  0.4984999274499807 -0.0042119937553935   T   T   T
 0.0000000000000000  0.3333333333333357  0.8163320000000027   F   F   F
 0.2500000000000000  0.8333333333333357  0.8163320000000027   F   F   F
 0.5000000000000000  0.3333333333333357  0.8163320000000027   F   F   F
 0.7500000000000000  0.8333333333333357  0.8163320000000027   F   F   F
 0.0000000000000000  0.0000000000000000  0.7244999999999990   F   F   F
 0.5000000000000000  0.0000000000000000  0.7244999999999990   F   F   F
 0.2500000000000000  0.5000000000000000  0.7244999999999990   F   F   F
 0.7500000000000000  0.5000000000000000  0.7244999999999990   F   F   F
 0.2482356740199400  0.1654782268502578  0.9062529296958161   T   T   T
 0.5009132860004725  0.6636316193668282  0.9064355953423857   T   T   T
 0.7539530657635268  0.1663223227407938  0.9060012723629555   T   T   T
 0.0009758979761967  0.6690099310821259  0.9056338612876728   T   T   T
-0.0277157463451318  0.0225553001207913  0.0790077666183931   T   T   T
 0.1082021984236673  0.9403408004891211  0.0972959274417435   T   T   T
Pt Surface(111)-4 layers + Alloys + Adso
   1.00000000000000
     5.6235999999999997    0.0000000000000000    0.0000000000000000
     0.0000000000000000    4.8701999999999996    0.0000000000000000
     0.0000000000000000    0.0000000000000000   25.0000000000000000
   Pt   W    O    H
   12     4     1     1
Selective dynamics
Direct
 0.0057183651915380 -0.0026305434872422 -0.0073559420735300   T   T   T
 0.5040747636607610 -0.0004662465615774 -0.0076041984848398   T   T   T
 0.2554562579161626  0.4981716356617770 -0.0078058095196364   T   T   T
 0.7503694575741091  0.4991622717654264 -0.0078051980080444   T   T   T
 0.0000000000000000  0.3333333333333357  0.8163320000000027   F   F   F
 0.2500000000000000  0.8333333333333357  0.8163320000000027   F   F   F
 0.5000000000000000  0.3333333333333357  0.8163320000000027   F   F   F
 0.7500000000000000  0.8333333333333357  0.8163320000000027   F   F   F
 0.0000000000000000  0.0000000000000000  0.7244999999999990   F   F   F
 0.5000000000000000  0.0000000000000000  0.7244999999999990   F   F   F
 0.2500000000000000  0.5000000000000000  0.7244999999999990   F   F   F
 0.7500000000000000  0.5000000000000000  0.7244999999999990   F   F   F
 0.2497213457842984  0.1649172862590584  0.9040743505352702   T   T   T
 0.5010576389183773  0.6620150663936586  0.9040032428128031   T   T   T
 0.7532305852087846  0.1659526350164991  0.9039432330599092   T   T   T
 0.0005556462329673  0.6733552114237243  0.9037190323257775   T   T   T
-0.0070589415643964  0.0108311207454477  0.0746996932037241   T   T   T
 0.1396504335215185  0.9345051896818846  0.0894855087837004   T   T   T
Pt Surface(111)-4 layers + Alloys + Adso
   1.00000000000000
     5.6235999999999997    0.0000000000000000    0.0000000000000000
     0.0000000000000000    4.8701999999999996    0.0000000000000000
     0.0000000000000000    0.0000000000000000   25.0000000000000000
   Pt   Re   O    H
   12     4     1     1
Selective dynamics
Direct
 0.0061057759500939 -0.0044437121262700 -0.0076623221792190   T   T   T
 0.5036002869826363  0.0007348298269273 -0.0101335420667442   T   T   T
 0.2552565897859021  0.4979456026368228 -0.0099727122528275   T   T   T
 0.7492580766540861  0.4984542708011284 -0.0099798062584220   T   T   T
 0.0000000000000000  0.3333333333333357  0.8163320000000027   F   F   F
 0.2500000000000000  0.8333333333333357  0.8163320000000027   F   F   F
 0.5000000000000000  0.3333333333333357  0.8163320000000027   F   F   F
 0.7500000000000000  0.8333333333333357  0.8163320000000027   F   F   F
 0.0000000000000000  0.0000000000000000  0.7244999999999990   F   F   F
 0.5000000000000000  0.0000000000000000  0.7244999999999990   F   F   F
 0.2500000000000000  0.5000000000000000  0.7244999999999990   F   F   F
 0.7500000000000000  0.5000000000000000  0.7244999999999990   F   F   F
 0.2458143116017931  0.1610438704124362  0.9024546768389254   T   T   T
 0.4990957114054684  0.6658212111482955  0.9017829171535851   T   T   T
 0.7599380952009661  0.1611125167656346  0.9023369119450945   T   T   T
-0.0014813357386556  0.6775851640390061  0.9024549194283978   T   T   T
-0.0058480116738971  0.0087902453090181  0.0737417916431992   T   T   T
 0.1420489372201968  0.9346327783879862  0.0884387062477600   T   T   T
Pt Surface(111)-4 layers + Alloys + Adso
   1.00000000000000
     5.6235999999999997    0.0000000000000000    0.0000000000000000
     0.0000000000000000    4.8701999999999996    0.0000000000000000
     0.0000000000000000    0.0000000000000000   25.0000000000000000
   Pt   Os   O    H
   12     4     1     1
Selective dynamics
Direct
 0.0053203924720904 -0.0062359860823084 -0.0074418601552837   T   T   T
 0.5027445709105621  0.0046956369398844 -0.0115025035852047   T   T   T
 0.2562991503843036  0.4985826981145264 -0.0115229807168457   T   T   T
 0.7487795708324877  0.4981981136059929 -0.0114458367976445   T   T   T
 0.0000000000000000  0.3333333333333357  0.8163320000000027   F   F   F
 0.2500000000000000  0.8333333333333357  0.8163320000000027   F   F   F
 0.5000000000000000  0.3333333333333357  0.8163320000000027   F   F   F
 0.7500000000000000  0.8333333333333357  0.8163320000000027   F   F   F
 0.0000000000000000  0.0000000000000000  0.7244999999999990   F   F   F
 0.5000000000000000  0.0000000000000000  0.7244999999999990   F   F   F
 0.2500000000000000  0.5000000000000000  0.7244999999999990   F   F   F
 0.7500000000000000  0.5000000000000000  0.7244999999999990   F   F   F
 0.2396388031970731  0.1584208403320985  0.9022392291658617   T   T   T
 0.5011009571023278  0.6713771533780430  0.9011272601610514   T   T   T
 0.7619350901979591  0.1585426290308068  0.9020258216079635   T   T   T
 0.0002834431149035  0.6743248992647706  0.9016449098098591   T   T   T
-0.0073172373488425  0.0066735781045226  0.0730994730699863   T   T   T
 0.1425323608953119  0.9330102655740475  0.0869169222158020   T   T   T
Pt Surface(111)-4 layers + Alloys + Adso
   1.00000000000000
     5.6235999999999997    0.0000000000000000    0.0000000000000000
     0.0000000000000000    4.8701999999999996    0.0000000000000000
     0.0000000000000000    0.0000000000000000   25.0000000000000000
   Pt   Ir   O    H
   12     4     1     1
Selective dynamics
Direct
 0.0067297805491456 -0.0064129435162361 -0.0043224944505316   T   T   T
 0.5042607512599792 -0.0000014740049292 -0.0088191928406970   T   T   T
 0.2561913123223569  0.4961597915797761 -0.0088366083106135   T   T   T
 0.7501401545035377  0.4959438275524173 -0.0088319065212645   T   T   T
 0.0000000000000000  0.3333333333333357  0.8163320000000027   F   F   F
 0.2500000000000000  0.8333333333333357  0.8163320000000027   F   F   F
 0.5000000000000000  0.3333333333333357  0.8163320000000027   F   F   F
 0.7500000000000000  0.8333333333333357  0.8163320000000027   F   F   F
 0.0000000000000000  0.0000000000000000  0.7244999999999990   F   F   F
 0.5000000000000000  0.0000000000000000  0.7244999999999990   F   F   F
 0.2500000000000000  0.5000000000000000  0.7244999999999990   F   F   F
 0.7500000000000000  0.5000000000000000  0.7244999999999990   F   F   F
 0.2467494848924819  0.1636016076410667  0.9037900896844950   T   T   T
 0.5012135268994029  0.6661159258456747  0.9027348873238914   T   T   T
 0.7542579712240288  0.1640958885619209  0.9034438857215203   T   T   T
 0.0007220816722614  0.6714267456992364  0.9041315018856064   T   T   T
-0.0073815704465347  0.0035414144368121  0.0758707848604423   T   T   T
 0.1454281761769504  0.9338329847936052  0.0888799915482787   T   T   T
Pt Surface(111)-4 layers + Alloys + Adso
   1.00000000000000
     5.6235999999999997    0.0000000000000000    0.0000000000000000
     0.0000000000000000    4.8701999999999996    0.0000000000000000
     0.0000000000000000    0.0000000000000000   25.0000000000000000
   Pt   O    H
   16     1     1
Selective dynamics
Direct
 0.0053732816460155 -0.0053023482003720  0.0042660383350895   T   T   T
 0.5031509247272058 -0.0023267514814837 -0.0019727014152610   T   T   T
 0.2555293978843732  0.4963895106681807 -0.0019692514277775   T   T   T
 0.7486452616773046  0.4965472234736725 -0.0020109039107308   T   T   T
 0.0000000000000000  0.3333333333333357  0.8163320000000027   F   F   F
 0.2500000000000000  0.8333333333333357  0.8163320000000027   F   F   F
 0.5000000000000000  0.3333333333333357  0.8163320000000027   F   F   F
 0.7500000000000000  0.8333333333333357  0.8163320000000027   F   F   F
 0.0000000000000000  0.0000000000000000  0.7244999999999990   F   F   F
 0.5000000000000000  0.0000000000000000  0.7244999999999990   F   F   F
 0.2500000000000000  0.5000000000000000  0.7244999999999990   F   F   F
 0.7500000000000000  0.5000000000000000  0.7244999999999990   F   F   F
 0.2482072150341124  0.1648527877133912  0.9076760151025415   T   T   T
 0.5007502248848205  0.6649379337326973  0.9061241263708395   T   T   T
 0.7537392331099391  0.1639682418490437  0.9077775762559486   T   T   T
 0.0003313600629961  0.6702238606590986  0.9084013320362687   T   T   T
-0.0020696259486435  0.0030945730663570  0.0838368977178626   T   T   T
 0.1511376470548614  0.9305655000861816  0.0959763823650626   T   T   T
Pt Surface(111)-4 layers + Alloys + Adso
   1.00000000000000
     5.6235999999999997    0.0000000000000000    0.0000000000000000
     0.0000000000000000    4.8701999999999996    0.0000000000000000
     0.0000000000000000    0.0000000000000000   25.0000000000000000
   Pt   Au   O    H
   12     4     1     1
Selective dynamics
Direct
-0.0014492530074865 -0.0006735940022504  0.0135185902730644   T   T   T
 0.4982621692847519  0.0016639085045262  0.0074818927683958   T   T   T
 0.2518758646813561  0.5008693684727938  0.0079385954496139   T   T   T
 0.7445941601475627  0.5014689785053288  0.0077958183500149   T   T   T
 0.0000000000000000  0.3333333333333357  0.8163320000000027   F   F   F
 0.2500000000000000  0.8333333333333357  0.8163320000000027   F   F   F
 0.5000000000000000  0.3333333333333357  0.8163320000000027   F   F   F
 0.7500000000000000  0.8333333333333357  0.8163320000000027   F   F   F
 0.0000000000000000  0.0000000000000000  0.7244999999999990   F   F   F
 0.5000000000000000  0.0000000000000000  0.7244999999999990   F   F   F
 0.2500000000000000  0.5000000000000000  0.7244999999999990   F   F   F
 0.7500000000000000  0.5000000000000000  0.7244999999999990   F   F   F
```

```
 0.2473511311763316  0.1674694517731503  0.9125968475411461   T   T   T
 0.4986664362891403  0.6664109945717664  0.9114087913652525   T   T   T
 0.7512149872632460  0.1669587374194820  0.9129177308115854   T   T   T
-0.0013523605814501  0.6697933805759029  0.9136872409480128   T   T   T
 0.0055725832262467  0.0029231058727894  0.0927918281285419   T   T   T
 0.1604327491124924  0.9258156276121652  0.1028372517092940   T   T   T
Pt Surface(111)-4 layers + Alloys + Adso
   1.00000000000000
     5.6235999999999997    0.0000000000000000    0.0000000000000000
     0.0000000000000000    4.8701999999999996    0.0000000000000000
     0.0000000000000000    0.0000000000000000   25.0000000000000000
   Pt   Hg   O    H
    12    4    1    1
Selective dynamics
Direct
 0.0067643002148737 -0.0034755596277802  0.0174747122793316   T   T   T
 0.5056634384545849 -0.0053888006752695  0.0130396148404211   T   T   T
 0.2596686723405551  0.4954658662567111  0.0140238275203362   T   T   T
 0.7503548234345236  0.4956488014423713  0.0140758107935940   T   T   T
 0.0000000000000000  0.3333333333333357  0.8163320000000027   F   F   F
 0.2500000000000000  0.8333333333333357  0.8163320000000027   F   F   F
 0.5000000000000000  0.3333333333333357  0.8163320000000027   F   F   F
 0.7500000000000000  0.8333333333333357  0.8163320000000027   F   F   F
 0.0000000000000000  0.0000000000000000  0.7244999999999990   F   F   F
 0.5000000000000000  0.0000000000000000  0.7244999999999990   F   F   F
 0.2500000000000000  0.5000000000000000  0.7244999999999990   F   F   F
 0.7500000000000000  0.5000000000000000  0.7244999999999990   F   F   F
 0.2469436036195735  0.1684373800252174  0.9154892319768156   T   T   T
 0.4990310912799349  0.6676415417659278  0.9138083123825673   T   T   T
 0.7506939208971876  0.1690086710753121  0.9155456102939206   T   T   T
-0.0009808437909814  0.6712802164708709  0.9176995919585376   T   T   T
-0.0031115427528476  0.0057736901318698  0.0976662711535502   T   T   T
 0.1475456156181215  0.9268257964002381  0.1098880180355899   T   T   T
```

331 *CO

```
Surface(331)-4 layers
   1.00000000000000
     8.4352800000000006    0.0000000000000000    0.0000000000000000
    -1.4058800000000000    6.1280900000000003    0.0000000000000000
     0.0000000000000000    0.0000000000000000   25.0000000000000000
   Pt   V    O    C
    27    9    1    1
Selective dynamics
Direct
-0.0004484682560623 -0.0008368148622428 -0.0110502787945383   T   T   T
 0.1047810863631299  0.6390622151220621  0.9456379741130214   T   T   T
 0.8804756061604274  0.2680972635518827  0.9071201136637556   T   T   T
 0.3356325420799692 -0.0022721351793647 -0.0202297541922850   T   T   T
 0.4384709208917983  0.6326425195598492  0.9456508451187745   T   T   T
 0.2096409460506451  0.2660783463456083  0.9072595042131565   T   T   T
 0.6637128614611691 -0.0023563545829877 -0.0202014169185764   T   T   T
 0.7750392404922413  0.6391008264992034  0.9450798385891390   T   T   T
 0.5451469756198047  0.2725489542275850  0.9056146354849026   T   T   T
 0.9649129391594045  0.7894776349564054  0.7810599999999965   F   F   F
 0.0701768413975614  0.4210610483853827  0.7445680000000010   F   F   F
 0.8421020476201875  0.0526265116863485  0.7080800000000025   F   F   F
 0.9473680488809180  0.6842082932855078  0.6715879999999999   F   F   F
 0.2982510144811457  0.7894776349564054  0.7810599999999965   F   F   F
 0.4035149167193097  0.4210610483853827  0.7445680000000010   F   F   F
 0.1754401229419287  0.0526265116863485  0.7080800000000025   F   F   F
 0.2807061242026592  0.6842082932855078  0.6715879999999999   F   F   F
 0.6315772348318589  0.7894776349564054  0.7810599999999965   F   F   F
 0.7368411370700230  0.4210610483853827  0.7445680000000010   F   F   F
 0.5087663432926490  0.0526265116863485  0.7080800000000025   F   F   F
 0.6140323445533795  0.6842082932855078  0.6715879999999999   F   F   F
 0.0526319511190820  0.3157917067144922  0.6350959999999972   F   F   F
 0.7192938757973408  0.3157917067144922  0.6350959999999972   F   F   F
 0.8245598770580713  0.9473734883136515  0.5986079999999987   F   F   F
 0.1578979523798125  0.9473734883136515  0.5986079999999987   F   F   F
 0.3859700264408232  0.3157917067144922  0.6350959999999972   F   F   F
 0.4912241727305329  0.9473734883136515  0.5986079999999987   F   F   F
 0.9841856506190679  0.9039644521750223  0.8844618663760935   T   T   T
 0.0907279448525393  0.5372225902463846  0.8459063927020397   T   T   T
 0.3184416816228922  0.9041736863584190  0.8846192846485408   T   T   T
 0.4219800563240689  0.5414750830275292  0.8454779107511187   T   T   T
 0.6503556269207625  0.9060879786134797  0.8846275719426522   T   T   T
 0.7578921187176004  0.5424967582009722  0.8454414613859546   T   T   T
 0.8573414919100454  0.1402380763897437  0.8069918384818255   T   T   T
 0.1885263730117118  0.1409833559909961  0.8068083284375100   T   T   T
 0.5243693239612591  0.1414603783103399  0.8064888779080597   T   T   T
 0.0005735771162840  0.0042658140159630  0.1115260860146936   T   T   T
 0.0018367963333821  0.0128603717641936  0.0651081212317478   T   T   T
Surface(331)-4 layers
   1.00000000000000
     8.4352800000000006    0.0000000000000000    0.0000000000000000
    -1.4058800000000000    6.1280900000000003    0.0000000000000000
     0.0000000000000000    0.0000000000000000   25.0000000000000000
   Pt   Cr   O    C
    27    9    1    1
Selective dynamics
Direct
 0.0009205803561755  0.0067919566856224  0.9823455400176819   T   T   T
 0.1044008529159204  0.6563792968112273  0.9342374969970242   T   T   T
 0.8785617331720873  0.2821994893069764  0.9061575429113019   T   T   T
 0.3313543033063275  0.9918978793348584  0.9735897257743142   T   T   T
 0.4360716631522038  0.6391412399707556  0.9402917190328809   T   T   T
 0.2113338961676317  0.2672484840908766  0.9051968052425088   T   T   T
 0.6611018765532251  0.9981135144741333  0.9767852892256427   T   T   T
 0.7771795659841857  0.6496979118187712  0.9415941344158061   T   T   T
 0.5491233267122981  0.2783837718592675  0.9082285633122497   T   T   T
 0.9649129391594045  0.7894776349564054  0.7810599999999965   F   F   F
 0.0701768413975614  0.4210610483853827  0.7445680000000010   F   F   F
 0.8421020476201875  0.0526265116863485  0.7080800000000025   F   F   F
 0.9473680488809180  0.6842082932855078  0.6715879999999999   F   F   F
 0.2982510144811457  0.7894776349564054  0.7810599999999965   F   F   F
 0.4035149167193097  0.4210610483853827  0.7445680000000010   F   F   F
 0.1754401229419287  0.0526265116863485  0.7080800000000025   F   F   F
 0.2807061242026592  0.6842082932855078  0.6715879999999999   F   F   F
 0.6315772348318589  0.7894776349564054  0.7810599999999965   F   F   F
 0.7368411370700230  0.4210610483853827  0.7445680000000010   F   F   F
 0.5087663432926490  0.0526265116863485  0.7080800000000025   F   F   F
 0.6140323445533795  0.6842082932855078  0.6715879999999999   F   F   F
 0.0526319511190820  0.3157917067144922  0.6350959999999972   F   F   F
 0.7192938757973408  0.3157917067144922  0.6350959999999972   F   F   F
 0.8245598770580713  0.9473734883136515  0.5986079999999987   F   F   F
 0.1578979523798125  0.9473734883136515  0.5986079999999987   F   F   F
 0.3859700264408232  0.3157917067144922  0.6350959999999972   F   F   F
 0.4912241727305329  0.9473734883136515  0.5986079999999987   F   F   F
 0.9545411349613391  0.9329922945596114  0.8765710577347416   T   T   T
 0.0846638684427257  0.4882442472805365  0.8405911215811063   T   T   T
 0.3524088732891470  0.9416914445309758  0.8745197602147919   T   T   T
 0.4517767904736796  0.5272834527365973  0.8426679312502623   T   T   T
 0.6480354889049715  0.9058415478415381  0.8802684701242650   T   T   T
 0.7285681488024187  0.5381592693869740  0.8438841808965067   T   T   T
 0.8844074921609208  0.1502277892394871  0.8085950129916070   T   T   T
 0.1664825001343194  0.1529032175828096  0.8075383261785727   T   T   T
 0.5128437487335735  0.1354139203285575  0.8098610311870739   T   T   T
 0.0031685021219526  0.0143674866373229  0.1042550387920869   T   T   T
 0.0053990604061733  0.0182365663786837  0.0577724350376728   T   T   T
Surface(331)-4 layers
   1.00000000000000
     8.4352800000000006    0.0000000000000000    0.0000000000000000
    -1.4058800000000000    6.1280900000000003    0.0000000000000000
     0.0000000000000000    0.0000000000000000   25.0000000000000000
   Pt   Mn   O    C
    27    9    1    1
Selective dynamics
Direct
 0.0082854770887128  0.0108096577238656  0.9794240361985511   T   T   T
 0.1176503147974382  0.6693425579245662  0.9301030057603157   T   T   T
 0.8854600257625412  0.2858881051303608  0.9077870891054439   T   T   T
 0.3399168483703239  0.0088728685525228  0.9707108487682654   T   T   T
 0.4429651221779603  0.6660199310060609  0.9278098301821924   T   T   T
 0.2183690623855655  0.2891594427406949  0.9022626039891034   T   T   T
 0.6664344736258335  0.9948960485232479  0.9730314178989140   T   T   T
 0.7875638785583332  0.6532964717438047  0.9391568240117634   T   T   T
 0.5626800430447960  0.2800848169628497  0.9075557211691577   T   T   T
 0.9649129391594045  0.7894776349564054  0.7810599999999965   F   F   F
 0.0701768413975614  0.4210610483853827  0.7445680000000010   F   F   F
 0.8421020476201875  0.0526265116863485  0.7080800000000025   F   F   F
 0.9473680488809180  0.6842082932855078  0.6715879999999999   F   F   F
 0.2982510144811457  0.7894776349564054  0.7810599999999965   F   F   F
 0.4035149167193097  0.4210610483853827  0.7445680000000010   F   F   F
 0.1754401229419287  0.0526265116863485  0.7080800000000025   F   F   F
 0.2807061242026592  0.6842082932855078  0.6715879999999999   F   F   F
 0.6315772348318589  0.7894776349564054  0.7810599999999965   F   F   F
 0.7368411370700230  0.4210610483853827  0.7445680000000010   F   F   F
 0.5087663432926490  0.0526265116863485  0.7080800000000025   F   F   F
 0.6140323445533795  0.6842082932855078  0.6715879999999999   F   F   F
 0.0526319511190820  0.3157917067144922  0.6350959999999972   F   F   F
 0.7192938757973408  0.3157917067144922  0.6350959999999972   F   F   F
 0.8245598770580713  0.9473734883136515  0.5986079999999987   F   F   F
 0.1578979523798125  0.9473734883136515  0.5986079999999987   F   F   F
 0.3859700264408232  0.3157917067144922  0.6350959999999972   F   F   F
 0.4912241727305329  0.9473734883136515  0.5986079999999987   F   F   F
 0.9740504854873752  0.9421243580913998  0.8752528914326165   T   T   T
 0.0715327815111051  0.4893720349574907  0.8388774955436611   T   T   T
 0.3365521481610965  0.9749637685522634  0.8710598758623767   T   T   T
 0.4617792382006508  0.5004880775395855  0.8388802417979980   T   T   T
 0.6994695748215941  0.9243428692475377  0.8750143641541064   T   T   T
 0.7400962677150306  0.5405198838019061  0.8426961959626433   T   T   T
 0.8854474723273590  0.1636753345881740  0.8108933243009001   T   T   T
 0.1661070727139969  0.1411197449037326  0.8087635110514533   T   T   T
 0.5042924673867107  0.1466435454728320  0.8115844978443859   T   T   T
 0.0222730113740697  0.0540694837068535  0.1006130726307345   T   T   T
 0.0191836665302161  0.0411615374242756  0.0542282289286820   T   T   T
Surface(331)-4 layers
   1.00000000000000
     8.4352800000000006    0.0000000000000000    0.0000000000000000
    -1.4058800000000000    6.1280900000000003    0.0000000000000000
     0.0000000000000000    0.0000000000000000   25.0000000000000000
   Pt   Fe   O    C
    27    9    1    1
Selective dynamics
Direct
-0.0003989424468258  0.0103417765924323 -0.0225331470286357   T   T   T
 0.1099655786579246  0.6679274729034237  0.9302927373200390   T   T   T
 0.8837100411185759  0.2989538789611473  0.9050829942754649   T   T   T
 0.3356713382572898  0.0027342433445595 -0.0281905545415469   T   T   T
 0.4448720199846973  0.6652426927213215  0.9293091479495839   T   T   T
 0.2141100456214484  0.2872754544084499  0.9069041325198488   T   T   T
 0.6660619334018435  0.0050609328325197 -0.0285428547668959   T   T   T
 0.7784326244830100  0.6645678092820436  0.9307727095113441   T   T   T
 0.5463250013786374  0.2791255450734081  0.9064554693222245   T   T   T
 0.9649129391594045  0.7894776349564054  0.7810599999999965   F   F   F
 0.0701768413975614  0.4210610483853827  0.7445680000000010   F   F   F
 0.8421020476201875  0.0526265116863485  0.7080800000000025   F   F   F
 0.9473680488809180  0.6842082932855078  0.6715879999999999   F   F   F
 0.2982510144811457  0.7894776349564054  0.7810599999999965   F   F   F
 0.4035149167193097  0.4210610483853827  0.7445680000000010   F   F   F
 0.1754401229419287  0.0526265116863485  0.7080800000000025   F   F   F
 0.2807061242026592  0.6842082932855078  0.6715879999999999   F   F   F
 0.6315772348318589  0.7894776349564054  0.7810599999999965   F   F   F
 0.7368411370700230  0.4210610483853827  0.7445680000000010   F   F   F
 0.5087663432926490  0.0526265116863485  0.7080800000000025   F   F   F
 0.6140323445533795  0.6842082932855078  0.6715879999999999   F   F   F
 0.0526319511190820  0.3157917067144922  0.6350959999999972   F   F   F
 0.7192938757973408  0.3157917067144922  0.6350959999999972   F   F   F
 0.8245598770580713  0.9473734883136515  0.5986079999999987   F   F   F
 0.1578979523798125  0.9473734883136515  0.5986079999999987   F   F   F
 0.3859700264408232  0.3157917067144922  0.6350959999999972   F   F   F
 0.4912241727305329  0.9473734883136515  0.5986079999999987   F   F   F
 0.9889900834007274  0.9644200883110544  0.8740287377265439   T   T   T
 0.0954927714223942  0.4999543586095323  0.8386925346797351   T   T   T
 0.3160087021172659  0.9570470326843096  0.8726980610638455   T   T   T
 0.4233099551811585  0.4913916330864272  0.8389846897224488   T   T   T
 0.6757750749655430  0.9561244956068681  0.8729458972204007   T   T   T
 0.7301760827531385  0.5019603727235297  0.8389044611909631   T   T   T
 0.8672328507622264  0.1604653705825420  0.8090312205072289   T   T   T
 0.1830858292875551  0.1628011359226944  0.8098019880128802   T   T   T
 0.5312703678236225  0.1591777445545768  0.8094139159778789   T   T   T
 0.0029152286613282  0.0157437674425062  0.0990373257752192   T   T   T
 0.0024458730601879  0.0188394479726675  0.0525388483352396   T   T   T
Surface(331)-4 layers
   1.00000000000000
     8.4352800000000006    0.0000000000000000    0.0000000000000000
    -1.4058800000000000    6.1280900000000003    0.0000000000000000
     0.0000000000000000    0.0000000000000000   25.0000000000000000
   Pt   Co   O    C
    27    9    1    1
Selective dynamics
Direct
-0.0001379889594909  0.0034438326625256 -0.0227733814129984   T   T   T
 0.1081982173734037  0.6590891915143686  0.9323024693022117   T   T   T
 0.8824027066035918  0.2891269685438460  0.9058702558002009   T   T   T
 0.3368758648037654 -0.0004540259337618 -0.0295749319548224   T   T   T
 0.4416759329685406  0.6502352218840789  0.9347722449456243   T   T   T
 0.2139490968740475  0.2834206439686295  0.9064117787756524   T   T   T
 0.6635177657265567  0.0013555216310718 -0.0297084597067485   T   T   T
 0.7781390944769296  0.6579055107585106  0.9326313807050922   T   T   T
 0.5470664010357895  0.2880946863670670  0.9055370184797219   T   T   T
 0.9649129391594045  0.7894776349564054  0.7810599999999965   F   F   F
 0.0701768413975614  0.4210610483853827  0.7445680000000010   F   F   F
 0.8421020476201875  0.0526265116863485  0.7080800000000025   F   F   F
 0.9473680488809180  0.6842082932855078  0.6715879999999999   F   F   F
 0.2982510144811457  0.7894776349564054  0.7810599999999965   F   F   F
 0.4035149167193097  0.4210610483853827  0.7445680000000010   F   F   F
 0.1754401229419287  0.0526265116863485  0.7080800000000025   F   F   F
 0.2807061242026592  0.6842082932855078  0.6715879999999999   F   F   F
 0.6315772348318589  0.7894776349564054  0.7810599999999965   F   F   F
 0.7368411370700230  0.4210610483853827  0.7445680000000010   F   F   F
 0.5087663432926490  0.0526265116863485  0.7080800000000025   F   F   F
 0.6140323445533795  0.6842082932855078  0.6715879999999999   F   F   F
 0.0526319511190820  0.3157917067144922  0.6350959999999972   F   F   F
 0.7192938757973408  0.3157917067144922  0.6350959999999972   F   F   F
 0.8245598770580713  0.9473734883136515  0.5986079999999987   F   F   F
 0.1578979523798125  0.9473734883136515  0.5986079999999987   F   F   F
 0.3859700264408232  0.3157917067144922  0.6350959999999972   F   F   F
 0.4912241727305329  0.9473734883136515  0.5986079999999987   F   F   F
 0.9893198025118218  0.9506530899549243  0.8744703188994477   T   T   T
 0.0920695274750983  0.5166748783942267  0.8394162521782905   T   T   T
 0.3261440055455039  0.9401086008698415  0.8721722459754419   T   T   T
 0.4201290806520712  0.5235103963542612  0.8395966849040776   T   T   T
 0.6579118004320844  0.9430776547801232  0.8722386069252053   T   T   T
 0.7478412524080442  0.5194004416933031  0.8392935211344649   T   T   T
 0.8616236985249611  0.1558051105783332  0.8083528184500209   T   T   T
 0.1899508827376190  0.1538974516427000  0.8086008715340459   T   T   T
 0.5272450448895590  0.1557821981440468  0.8079896942671941   T   T   T
 0.0004654880054454  0.0039891283003976  0.0985509529108280   T   T   T
 0.0011074550896374  0.0093469880348146  0.0520704140695097   T   T   T
Surface(331)-4 layers
   1.00000000000000
     8.4352800000000006    0.0000000000000000    0.0000000000000000
    -1.4058800000000000    6.1280900000000003    0.0000000000000000
     0.0000000000000000    0.0000000000000000   25.0000000000000000
   Pt   Ni   O    C
    27    9    1    1
Selective dynamics
Direct
 0.0020597456113714  0.0121361038695132 -0.0228421351298279   T   T   T
 0.1094577255323821  0.6631462290227442  0.9350794343604054   T   T   T
 0.8842338754042965  0.2963048394383325  0.9071134965914804   T   T   T
 0.3384722381039035  0.0080659944924599 -0.0298541280489053   T   T   T
 0.4427853616350498  0.6570309003691372  0.9365662311666622   T   T   T
 0.2151842718082002  0.2940327384398749  0.9070291110728937   T   T   T
 0.6647851327060257  0.0089662600336386 -0.0298591644334586   T   T   T
 0.7793346518868358  0.6625929053906203  0.9349908406095162   T   T   T
 0.5492291550139277  0.2976605885056646  0.9063286894405609   T   T   T
 0.9649129391594045  0.7894776349564054  0.7810599999999965   F   F   F
 0.0701768413975614  0.4210610483853827  0.7445680000000010   F   F   F
 0.8421020476201875  0.0526265116863485  0.7080800000000025   F   F   F
 0.9473680488809180  0.6842082932855078  0.6715879999999999   F   F   F
 0.2982510144811457  0.7894776349564054  0.7810599999999965   F   F   F
 0.4035149167193097  0.4210610483853827  0.7445680000000010   F   F   F
 0.1754401229419287  0.0526265116863485  0.7080800000000025   F   F   F
 0.2807061242026592  0.6842082932855078  0.6715879999999999   F   F   F
 0.6315772348318589  0.7894776349564054  0.7810599999999965   F   F   F
 0.7368411370700230  0.4210610483853827  0.7445680000000010   F   F   F
 0.5087663432926490  0.0526265116863485  0.7080800000000025   F   F   F
 0.6140323445533795  0.6842082932855078  0.6715879999999999   F   F   F
 0.0526319511190820  0.3157917067144922  0.6350959999999972   F   F   F
 0.7192938757973408  0.3157917067144922  0.6350959999999972   F   F   F
 0.8245598770580713  0.9473734883136515  0.5986079999999987   F   F   F
 0.1578979523798125  0.9473734883136515  0.5986079999999987   F   F   F
 0.3859700264408232  0.3157917067144922  0.6350959999999972   F   F   F
 0.4912241727305329  0.9473734883136515  0.5986079999999987   F   F   F
 0.9909487956084606  0.9475887211155104  0.8757501065011735   T   T   T
 0.0900729013128104  0.5332391696812905  0.8406156474019678   T   T   T
 0.3240347621438317  0.9386706074586220  0.8721858167014291   T   T   T
 0.4224281489396414  0.5346714142555770  0.8404451954335368   T   T   T
 0.6569191602430537  0.9396125813038048  0.8723471112796608   T   T   T
 0.7547597850979622  0.5337594857398058  0.8403887133231099   T   T   T
 0.8622429733015078  0.1613738870432007  0.8085067668525485   T   T   T
 0.1915593345972432  0.1606362653202169  0.8084643053528739   T   T   T
 0.5273855499364255  0.1619877667338565  0.8082427598686490   T   T   T
 0.0013250760724020  0.0106226244473018  0.0982480170490388   T   T   T
 0.0026540967985330  0.0171685083986336  0.0518187486858571   T   T   T
Surface(331)-4 layers
   1.00000000000000
     8.4352800000000006    0.0000000000000000    0.0000000000000000
    -1.4058800000000000    6.1280900000000003    0.0000000000000000
     0.0000000000000000    0.0000000000000000   25.0000000000000000
   Pt   Cu   O    C
    27    9    1    1
Selective dynamics
Direct
-0.0005619399015722 -0.0078880671178749 -0.0164456434660262   T   T   T
 0.1043809995398087  0.6347081863883725  0.9441175027884171   T   T   T
 0.8804035927390939  0.2735585096143122  0.9151691468142810   T   T   T
 0.3335392039126719 -0.0172448813445341 -0.0242866339694269   T   T   T
 0.4366840922095552  0.6304784358660249  0.9452558576189537   T   T   T
 0.2102380839682864  0.2733142210055199  0.9145197671845985   T   T   T
 0.6602843847623798 -0.0186679437871203 -0.0244328731773558   T   T   T
 0.7756769324680349  0.6337975740390777  0.9446839699109415   T   T   T
 0.5463665565410962  0.2787023655906107  0.9156051549211098   T   T   T
 0.9649129391594045  0.7894776349564054  0.7810599999999965   F   F   F
 0.0701768413975614  0.4210610483853827  0.7445680000000010   F   F   F
 0.8421020476201875  0.0526265116863485  0.7080800000000025   F   F   F
 0.9473680488809180  0.6842082932855078  0.6715879999999999   F   F   F
 0.2982510144811457  0.7894776349564054  0.7810599999999965   F   F   F
 0.4035149167193097  0.4210610483853827  0.7445680000000010   F   F   F
 0.1754401229419287  0.0526265116863485  0.7080800000000025   F   F   F
 0.2807061242026592  0.6842082932855078  0.6715879999999999   F   F   F
 0.6315772348318589  0.7894776349564054  0.7810599999999965   F   F   F
 0.7368411370700230  0.4210610483853827  0.7445680000000010   F   F   F
 0.5087663432926490  0.0526265116863485  0.7080800000000025   F   F   F
 0.6140323445533795  0.6842082932855078  0.6715879999999999   F   F   F
 0.0526319511190820  0.3157917067144922  0.6350959999999972   F   F   F
 0.7192938757973408  0.3157917067144922  0.6350959999999972   F   F   F
 0.8245598770580713  0.9473734883136515  0.5986079999999987   F   F   F
 0.1578979523798125  0.9473734883136515  0.5986079999999987   F   F   F
 0.3859700264408232  0.3157917067144922  0.6350959999999972   F   F   F
 0.4912241727305329  0.9473734883136515  0.5986079999999987   F   F   F
 0.9849435718286033  0.9120388585051783  0.8799650896362428   T   T   T
 0.0875325411840419  0.5250316236280724  0.8449995739963923   T   T   T
 0.3188413375213153  0.9111245564059335  0.8765080045024171   T   T   T
 0.4214873111105769  0.5260664774283119  0.8452699316860237   T   T   T
 0.6512378813430404  0.9085768975282922  0.8766970807607682   T   T   T
 0.7536483813239399  0.5254657207391024  0.8452336022921263   T   T   T
 0.8604053606113938  0.1574536658992039  0.8127188429532896   T   T   T
 0.1916800347127999  0.1572223917589917  0.8123501555135975   T   T   T
 0.5268996074697936  0.1587305929249168  0.8124012379385132   T   T   T
-0.0008791113176665 -0.0062781093313797  0.1044702416373425   T   T   T
-0.0010284048765887 -0.0082870380626363  0.0580097857263975   T   T   T
Surface(331)-4 layers
   1.00000000000000
     8.4352800000000006    0.0000000000000000    0.0000000000000000
    -1.4058800000000000    6.1280900000000003    0.0000000000000000
     0.0000000000000000    0.0000000000000000   25.0000000000000000
   Pt   Zn   O    C
    27    9    1    1
Selective dynamics
Direct
-0.0003923420912654  0.0010422799244693 -0.0109554765626724   T   T   T
 0.1054346325171141  0.6396119797562008  0.9449591960109047   T   T   T
 0.8803226307269369  0.2722683248869345  0.9129433124758839   T   T   T
 0.3335032827422968 -0.0118362987647253 -0.0193864946657650   T   T   T
 0.4392033192708817  0.6364475195895734  0.9456143017251631   T   T   T
 0.2102626401004508  0.2677446848557253  0.9114497982722326   T   T   T
 0.6621679461065789 -0.0110703457853527 -0.0196225212536630   T   T   T
 0.7744012081890878  0.6385091224979563  0.9447220806051357   T   T   T
 0.5448498619392801  0.2697179568081882  0.9104186442145821   T   T   T
 0.9649129391594045  0.7894776349564054  0.7810599999999965   F   F   F
 0.0701768413975614  0.4210610483853827  0.7445680000000010   F   F   F
 0.8421020476201875  0.0526265116863485  0.7080800000000025   F   F   F
 0.9473680488809180  0.6842082932855078  0.6715879999999999   F   F   F
 0.2982510144811457  0.7894776349564054  0.7810599999999965   F   F   F
 0.4035149167193097  0.4210610483853827  0.7445680000000010   F   F   F
 0.1754401229419287  0.0526265116863485  0.7080800000000025   F   F   F
 0.2807061242026592  0.6842082932855078  0.6715879999999999   F   F   F
```

```
 0.6315772348318589 0.7894776349564054 0.7810599999999965  F  F  F
 0.7368411370700230 0.4210610483853827 0.7445680000000010  F  F  F
 0.5087663432926490 0.0526265116863485 0.7080800000000025  F  F  F
 0.6140323445533795 0.6842082932855078 0.6715879999999999  F  F  F
 0.0526319511190820 0.3157917067144922 0.6350959999999972  F  F  F
 0.7192938757973408 0.3157917067144922 0.6350959999999972  F  F  F
 0.8245598770580713 0.9473734883136515 0.5986079999999987  F  F  F
 0.1578979523798125 0.9473734883136515 0.5986079999999987  F  F  F
 0.3859700264408232 0.3157917067144922 0.6350959999999972  F  F  F
 0.4912241727305329 0.9473734883136515 0.5986079999999987  F  F  F
 0.9843584446612315 0.9085598375613019 0.8834399804485695  T  T  T
 0.0874794036359334 0.5259561975234872 0.8454627704157444  T  T  T
 0.3173991450849735 0.9066731609358231 0.8815586936711047  T  T  T
 0.4213917577134649 0.5252680806086798 0.8453427738850139  T  T  T
 0.6517541241807524 0.9057908529840115 0.8814400858196921  T  T  T
 0.7540966078228518 0.5260398014528057 0.8453013117976224  T  T  T
 0.8593921342310999 0.1526327145953643 0.8087900138205383  T  T  T
 0.1913008815254597 0.1522020607715095 0.8085466453064584  T  T  T
 0.5255015724942582 0.1528924145122230 0.8083225985961153  T  T  T
 0.0009759912703285 0.0061287929139034 0.1097246881275697  T  T  T
 0.0007986305878563 0.0066912016464169 0.0632370573602059  T  T  T
Surface(331)-4 layers
  1.00000000000000
    8.4352800000000006    0.0000000000000000    0.0000000000000000
   -1.4058800000000000    6.1280900000000003    0.0000000000000000
    0.0000000000000000    0.0000000000000000   25.0000000000000000
  Pt   Nb   O    C
  27    9    1    1
Selective dynamics
Direct
 -0.0001242524963630  0.0005572372403911  0.0095209600854859  T  T  T
 0.1054457783015532 0.6417192852235185 0.9653646840869017  T  T  T
 0.8809781714116076 0.2774049071416785 0.9277953189495660  T  T  T
 0.3359333260532498 0.0007958169271201 0.0011658350984332  T  T  T
 0.4394618058407524 0.6373336979622429 0.9658664828281739  T  T  T
 0.2127739641129875 0.2765320079710494 0.9276973035986528  T  T  T
 0.6642915276061966 0.0012389854124488 0.0011545298399381  T  T  T
 0.7754541902223615 0.6417893345976849 0.9650493472689353  T  T  T
 0.5453362578534557 0.2810501234835938 0.9265310272330651  T  T  T
 0.9649129391594045 0.7894776349564054 0.7810599999999965  F  F  F
 0.0701768413975614 0.4210610483853827 0.7445680000000010  F  F  F
 0.8421020476201875 0.0526265116863485 0.7080800000000025  F  F  F
 0.9473680488809180 0.6842082932855078 0.6715879999999999  F  F  F
 0.2982510144811457 0.7894776349564054 0.7810599999999965  F  F  F
 0.4035149167193097 0.4210610483853827 0.7445680000000010  F  F  F
 0.1754401229419287 0.0526265116863485 0.7080800000000025  F  F  F
 0.2807061242026592 0.6842082932855078 0.6715879999999999  F  F  F
 0.6315772348318589 0.7894776349564054 0.7810599999999965  F  F  F
 0.7368411370700230 0.4210610483853827 0.7445680000000010  F  F  F
 0.5087663432926490 0.0526265116863485 0.7080800000000025  F  F  F
 0.6140323445533795 0.6842082932855078 0.6715879999999999  F  F  F
 0.0526319511190820 0.3157917067144922 0.6350959999999972  F  F  F
 0.7192938757973408 0.3157917067144922 0.6350959999999972  F  F  F
 0.8245598770580713 0.9473734883136515 0.5986079999999987  F  F  F
 0.1578979523798125 0.9473734883136515 0.5986079999999987  F  F  F
 0.3859700264408232 0.3157917067144922 0.6350959999999972  F  F  F
 0.4912241727305329 0.9473734883136515 0.5986079999999987  F  F  F
 0.9854514716850243 0.9111599849722493 0.8991262636912057  T  T  T
 0.0883748880932628 0.5278002012755956 0.8553120261622684  T  T  T
 0.3191067855797678 0.9117350483554427 0.8999197794654739  T  T  T
 0.4222121272205698 0.5316541558634679 0.8547273314374587  T  T  T
 0.6523373796718515 0.9128905817358385 0.8998987183175131  T  T  T
 0.7548934816656608 0.5302088700122292 0.8547930320134475  T  T  T
 0.8578522647653328 0.1486483580834309 0.8120587414727352  T  T  T
 0.1913758957916250 0.1486579323224685 0.8120203099063669  T  T  T
 0.5248509285810837 0.1488682927853934 0.8121405040469382  T  T  T
 0.0009856383288884 0.0056611376197496 0.1317101111967726  T  T  T
 0.0028373592821714 0.0173874761005152 0.0853363666773898  T  T  T
Surface(331)-4 layers
  1.00000000000000
    8.4352800000000006    0.0000000000000000    0.0000000000000000
   -1.4058800000000000    6.1280900000000003    0.0000000000000000
    0.0000000000000000    0.0000000000000000   25.0000000000000000
  Pt   Mo   O    C
  27    9    1    1
Selective dynamics
Direct
 -0.0010461854124759 -0.0043611251275572 -0.0026791115137113  T  T  T
 0.1048304883942737 0.6351083647108285 0.9564960082041638  T  T  T
 0.8793519872952431 0.2699926741437159 0.9201298648402753  T  T  T
 0.3349309754821382 -0.0060194261421645 -0.0093128520487442  T  T  T
 0.4375599465116124 0.6302384194750530 0.9565621567317807  T  T  T
 0.2102965648344880 0.2681860151072974 0.9200681583654481  T  T  T
 0.6627800996020508 -0.0061367337595808 -0.0092149008822364  T  T  T
 0.7733576971772911 0.6349914418751517 0.9560713844857321  T  T  T
 0.5447793774512774 0.2717967765387225 0.9193167448521644  T  T  T
 0.9649129391594045 0.7894776349564054 0.7810599999999965  F  F  F
 0.0701768413975614 0.4210610483853827 0.7445680000000010  F  F  F
 0.8421020476201875 0.0526265116863485 0.7080800000000025  F  F  F
 0.9473680488809180 0.6842082932855078 0.6715879999999999  F  F  F
 0.2982510144811457 0.7894776349564054 0.7810599999999965  F  F  F
 0.4035149167193097 0.4210610483853827 0.7445680000000010  F  F  F
 0.1754401229419287 0.0526265116863485 0.7080800000000025  F  F  F
 0.2807061242026592 0.6842082932855078 0.6715879999999999  F  F  F
 0.6315772348318589 0.7894776349564054 0.7810599999999965  F  F  F
 0.7368411370700230 0.4210610483853827 0.7445680000000010  F  F  F
 0.5087663432926490 0.0526265116863485 0.7080800000000025  F  F  F
 0.6140323445533795 0.6842082932855078 0.6715879999999999  F  F  F
 0.0526319511190820 0.3157917067144922 0.6350959999999972  F  F  F
 0.7192938757973408 0.3157917067144922 0.6350959999999972  F  F  F
 0.8245598770580713 0.9473734883136515 0.5986079999999987  F  F  F
 0.1578979523798125 0.9473734883136515 0.5986079999999987  F  F  F
 0.3859700264408232 0.3157917067144922 0.6350959999999972  F  F  F
 0.4912241727305329 0.9473734883136515 0.5986079999999987  F  F  F
 0.9839001487671889 0.9052994274557861 0.8889491413076689  T  T  T
 0.0874778231176926 0.5248447818182503 0.8509048853092441  T  T  T
 0.3182959734739798 0.9061079455713502 0.8898896361653479  T  T  T
 0.4222547348082061 0.5259751126417123 0.8501263515436919  T  T  T
 0.6498729756727624 0.9064868802666345 0.8901137829234802  T  T  T
 0.7532892690909446 0.5267526001068854 0.8503797223414969  T  T  T
 0.8590291270133512 0.1498076225630459 0.8114878156236033  T  T  T
 0.1914423691247030 0.1497089859420289 0.8114070987792346  T  T  T
 0.5248097273808011 0.1495198976786802 0.8114561759189788  T  T  T
 0.0010242101010702 0.0064395618347262 0.1197341384291362  T  T  T
 0.0020328442968786 0.0130923687234773 0.0733118820084871  T  T  T
Surface(331)-4 layers
  1.00000000000000
    8.4352800000000006    0.0000000000000000    0.0000000000000000
   -1.4058800000000000    6.1280900000000003    0.0000000000000000
    0.0000000000000000    0.0000000000000000   25.0000000000000000
  Pt   Tc   O    C
  27    9    1    1
Selective dynamics
Direct
 -0.0024913860349337 -0.0095408546466473 -0.0072062104026542  T  T  T
 0.1042246424592117 0.6308574722739471 0.9541129823891694  T  T  T
 0.8790350007199044 0.2687322801971808 0.9185072138231102  T  T  T
 0.3344084271885479 -0.0146866241345145 -0.0147085720672189  T  T  T
 0.4379279699182855 0.6275952256566585 0.9527843604357211  T  T  T
 0.2097298896007072 0.2678347858367064 0.9186464136465715  T  T  T
 0.6605174980809312 -0.0141017282909014 -0.0145039763686885  T  T  T
 0.7712022874953057 0.6296225908343974 0.9534640921014911  T  T  T
 0.5433881805848857 0.2664986558172480 0.9171876579043117  T  T  T
 0.9649129391594045 0.7894776349564054 0.7810599999999965  F  F  F
 0.0701768413975614 0.4210610483853827 0.7445680000000010  F  F  F
 0.8421020476201875 0.0526265116863485 0.7080800000000025  F  F  F
 0.9473680488809180 0.6842082932855078 0.6715879999999999  F  F  F
 0.2982510144811457 0.7894776349564054 0.7810599999999965  F  F  F
 0.4035149167193097 0.4210610483853827 0.7445680000000010  F  F  F
 0.1754401229419287 0.0526265116863485 0.7080800000000025  F  F  F
 0.2807061242026592 0.6842082932855078 0.6715879999999999  F  F  F
 0.6315772348318589 0.7894776349564054 0.7810599999999965  F  F  F
 0.7368411370700230 0.4210610483853827 0.7445680000000010  F  F  F
 0.5087663432926490 0.0526265116863485 0.7080800000000025  F  F  F
 0.6140323445533795 0.6842082932855078 0.6715879999999999  F  F  F
 0.0526319511190820 0.3157917067144922 0.6350959999999972  F  F  F
 0.7192938757973408 0.3157917067144922 0.6350959999999972  F  F  F
 0.8245598770580713 0.9473734883136515 0.5986079999999987  F  F  F
 0.1578979523798125 0.9473734883136515 0.5986079999999987  F  F  F
 0.3859700264408232 0.3157917067144922 0.6350959999999972  F  F  F
 0.4912241727305329 0.9473734883136515 0.5986079999999987  F  F  F
 0.9807871786057991 0.8977876704376890 0.8860525366802717  T  T  T
 0.0881076927356440 0.5302635662094703 0.8495797814728659  T  T  T
 0.3036585649382013 0.9031638855803661 0.8824138685423168  T  T  T
 0.4194648260280839 0.5165259478838020 0.8476451509008577  T  T  T
 0.6624085422625772 0.9055970334610058 0.8827444368876334  T  T  T
 0.7538178745158766 0.5263130343284024 0.8483796884355547  T  T  T
 0.8512166952151493 0.1522016301928598 0.8147594553946210  T  T  T
 0.2021612614372583 0.1552358881117359 0.8151235876496672  T  T  T
 0.5260045304440638 0.1512410140059697 0.8133402089562023  T  T  T
 0.0019307863344941 0.0107412937900750 0.1147507449895476  T  T  T
 0.0023123919266765 0.0136574043174560 0.0682856455817588  T  T  T
Surface(331)-4 layers
  1.00000000000000
    8.4352800000000006    0.0000000000000000    0.0000000000000000
   -1.4058800000000000    6.1280900000000003    0.0000000000000000
    0.0000000000000000    0.0000000000000000   25.0000000000000000
  Pt   Ru   O    C
  27    9    1    1
Selective dynamics
Direct
 -0.0021677493887199 -0.0111125099117227 -0.0093215587321583  T  T  T
 0.1047060028911238 0.6324486427623306 0.9526332916486643  T  T  T
 0.8789987806168984 0.2711225742180488 0.9190456775739411  T  T  T
 0.3342344829065994 -0.0136145627432909 -0.0157398637877630  T  T  T
 0.4375172700950253 0.6282401959987118 0.9531981343662379  T  T  T
 0.2117250937841356 0.2690584435513486 0.9189686226621574  T  T  T
 0.6612188059695109 -0.0124906298832096 -0.0157367826978998  T  T  T
 0.7730861756668941 0.6320008653667074 0.9525144432346847  T  T  T
 0.5447111300364839 0.2724975803293501 0.9183621399106203  T  T  T
 0.9649129391594045 0.7894776349564054 0.7810599999999965  F  F  F
 0.0701768413975614 0.4210610483853827 0.7445680000000010  F  F  F
 0.8421020476201875 0.0526265116863485 0.7080800000000025  F  F  F
 0.9473680488809180 0.6842082932855078 0.6715879999999999  F  F  F
 0.2982510144811457 0.7894776349564054 0.7810599999999965  F  F  F
 0.4035149167193097 0.4210610483853827 0.7445680000000010  F  F  F
 0.1754401229419287 0.0526265116863485 0.7080800000000025  F  F  F
 0.2807061242026592 0.6842082932855078 0.6715879999999999  F  F  F
 0.6315772348318589 0.7894776349564054 0.7810599999999965  F  F  F
 0.7368411370700230 0.4210610483853827 0.7445680000000010  F  F  F
 0.5087663432926490 0.0526265116863485 0.7080800000000025  F  F  F
 0.6140323445533795 0.6842082932855078 0.6715879999999999  F  F  F
 0.0526319511190820 0.3157917067144922 0.6350959999999972  F  F  F
 0.7192938757973408 0.3157917067144922 0.6350959999999972  F  F  F
 0.8245598770580713 0.9473734883136515 0.5986079999999987  F  F  F
 0.1578979523798125 0.9473734883136515 0.5986079999999987  F  F  F
 0.3859700264408232 0.3157917067144922 0.6350959999999972  F  F  F
 0.4912241727305329 0.9473734883136515 0.5986079999999987  F  F  F
 0.9846935307112770 0.9106638726136704 0.8836476764157420  T  T  T
 0.0885299647348453 0.5236582930395225 0.8479164284293131  T  T  T
 0.3173593039440881 0.9045346376668473 0.8812298656620762  T  T  T
 0.4211498446513812 0.5250875096848102 0.8479145156367777  T  T  T
 0.6514447732155245 0.9049433794382429 0.8813708933422816  T  T  T
 0.7526618254663040 0.5246842798093041 0.8478536144671283  T  T  T
 0.8600716064916852 0.1552471932291110 0.8158465871513965  T  T  T
 0.1911468210792066 0.1544221610391397 0.8156635157766382  T  T  T
 0.5265263303147636 0.1549430843921952 0.8152113224470596  T  T  T
 0.0010192119199815 0.0069486860126557 0.1120530637505555  T  T  T
 0.0011023240991939 0.0084040614228310 0.0655802438728024  T  T  T
Surface(331)-4 layers
  1.00000000000000
    8.4352800000000006    0.0000000000000000    0.0000000000000000
   -1.4058800000000000    6.1280900000000003    0.0000000000000000
    0.0000000000000000    0.0000000000000000   25.0000000000000000
  Pt   Rh   O    C
  27    9    1    1
Selective dynamics
Direct
 -0.0022969125577157 -0.0126194067210502 -0.0079813192556495  T  T  T
 0.1053197192032169 0.6319034106616864 0.9542756881984296  T  T  T
 0.8796746373700763 0.2733893745662397 0.9233081221393951  T  T  T
 0.3346520384540395 -0.0153792822300337 -0.0145773846744311  T  T  T
 0.4375240014194758 0.6278559996483460 0.9551036529799136  T  T  T
 0.2116890703680467 0.2709067478087193 0.9229360548072846  T  T  T
 0.6603632315652843 -0.0143864791635060 -0.0145513949918731  T  T  T
 0.7725153943237248 0.6316596532404800 0.9541256996099641  T  T  T
 0.5450583342817690 0.2743590474913677 0.9222553995063308  T  T  T
 0.9649129391594045 0.7894776349564054 0.7810599999999965  F  F  F
 0.0701768413975614 0.4210610483853827 0.7445680000000010  F  F  F
 0.8421020476201875 0.0526265116863485 0.7080800000000025  F  F  F
 0.9473680488809180 0.6842082932855078 0.6715879999999999  F  F  F
 0.2982510144811457 0.7894776349564054 0.7810599999999965  F  F  F
 0.4035149167193097 0.4210610483853827 0.7445680000000010  F  F  F
 0.1754401229419287 0.0526265116863485 0.7080800000000025  F  F  F
 0.2807061242026592 0.6842082932855078 0.6715879999999999  F  F  F
 0.6315772348318589 0.7894776349564054 0.7810599999999965  F  F  F
 0.7368411370700230 0.4210610483853827 0.7445680000000010  F  F  F
 0.5087663432926490 0.0526265116863485 0.7080800000000025  F  F  F
 0.6140323445533795 0.6842082932855078 0.6715879999999999  F  F  F
 0.0526319511190820 0.3157917067144922 0.6350959999999972  F  F  F
 0.7192938757973408 0.3157917067144922 0.6350959999999972  F  F  F
 0.8245598770580713 0.9473734883136515 0.5986079999999987  F  F  F
 0.1578979523798125 0.9473734883136515 0.5986079999999987  F  F  F
 0.3859700264408232 0.3157917067144922 0.6350959999999972  F  F  F
 0.4912241727305329 0.9473734883136515 0.5986079999999987  F  F  F
 0.9846185923341793 0.9101136773295085 0.8859559663100882  T  T  T
 0.0869567516760420 0.5238806099637996 0.8492374366296405  T  T  T
 0.3176890907344593 0.9030963768986712 0.8819638875002795  T  T  T
 0.4207195927854754 0.5234009360606069 0.8490450600221152  T  T  T
 0.6506836246961081 0.9046191460650921 0.8821059519928818  T  T  T
 0.7538014540352281 0.5244932880395772 0.8490024082670365  T  T  T
 0.8609301542744380 0.1584366892181974 0.8152382465314535  T  T  T
 0.1914723950511624 0.1570278187749669 0.8151501327060366  T  T  T
 0.5264768445267159 0.1589586429130007 0.8147547411701532  T  T  T
 0.0010658632859990 0.0069437185657588 0.1129838347014343  T  T  T
 0.0007599461495389 0.0056259411477128 0.0665235383955507  T  T  T
Surface(331)-4 layers
  1.00000000000000
    8.4352800000000006    0.0000000000000000    0.0000000000000000
   -1.4058800000000000    6.1280900000000003    0.0000000000000000
    0.0000000000000000    0.0000000000000000   25.0000000000000000
  Pt   Pd   O    C
  27    9    1    1
Selective dynamics
Direct
 -0.0027774278924390 -0.0160324494842200 -0.0026918835099655  T  T  T
 0.1052013025877913 0.6264948307239574 0.9625516968872577  T  T  T
 0.8796950498518069 0.2749925833649203 0.9307741160758434  T  T  T
 0.3343281222586669 -0.0190386280011960 -0.0091162540271702  T  T  T
 0.4370125195284834 0.6244132957164366 0.9622085652882149  T  T  T
 0.2114178964113775 0.2732678090575752 0.9300340756140177  T  T  T
 0.6589349014384990 -0.0186166458417512 -0.0090701242964630  T  T  T
 0.7698534938541363 0.6257119586738238 0.9626407209240095  T  T  T
 0.5454068753305996 0.2753780566822072 0.9297419452791721  T  T  T
 0.9649129391594045 0.7894776349564054 0.7810599999999965  F  F  F
 0.0701768413975614 0.4210610483853827 0.7445680000000010  F  F  F
 0.8421020476201875 0.0526265116863485 0.7080800000000025  F  F  F
 0.9473680488809180 0.6842082932855078 0.6715879999999999  F  F  F
 0.2982510144811457 0.7894776349564054 0.7810599999999965  F  F  F
 0.4035149167193097 0.4210610483853827 0.7445680000000010  F  F  F
 0.1754401229419287 0.0526265116863485 0.7080800000000025  F  F  F
 0.2807061242026592 0.6842082932855078 0.6715879999999999  F  F  F
 0.6315772348318589 0.7894776349564054 0.7810599999999965  F  F  F
 0.7368411370700230 0.4210610483853827 0.7445680000000010  F  F  F
 0.5087663432926490 0.0526265116863485 0.7080800000000025  F  F  F
 0.6140323445533795 0.6842082932855078 0.6715879999999999  F  F  F
 0.0526319511190820 0.3157917067144922 0.6350959999999972  F  F  F
 0.7192938757973408 0.3157917067144922 0.6350959999999972  F  F  F
 0.8245598770580713 0.9473734883136515 0.5986079999999987  F  F  F
 0.1578979523798125 0.9473734883136515 0.5986079999999987  F  F  F
 0.3859700264408232 0.3157917067144922 0.6350959999999972  F  F  F
 0.4912241727305329 0.9473734883136515 0.5986079999999987  F  F  F
 0.9842238167964630 0.9075839303637762 0.8918917211928237  T  T  T
 0.0870191055026124 0.5284691001065521 0.8537869130305289  T  T  T
 0.3174607395362983 0.9026767325663313 0.8861323105922317  T  T  T
 0.4208388181166238 0.5252701581292878 0.8531255846078096  T  T  T
 0.6505280749604148 0.9036534505637911 0.8862527305323220  T  T  T
 0.7556999807242063 0.5287655117135042 0.8535110951298674  T  T  T
 0.8616056160864328 0.1598342656155901 0.8168959193987265  T  T  T
 0.1911212429076811 0.1588578848258693 0.8166784980532058  T  T  T
 0.5271650540271398 0.1621381232908725 0.8159231428693101  T  T  T
 0.0002499929030250 0.0019894651969608 0.1180143183988049  T  T  T
 -0.0006275794715363 -0.0027682045702936  0.0715719623342852  T  T  T
Surface(331)-4 layers
  1.00000000000000
    8.4352800000000006    0.0000000000000000    0.0000000000000000
   -1.4058800000000000    6.1280900000000003    0.0000000000000000
    0.0000000000000000    0.0000000000000000   25.0000000000000000
  Pt   Ag   O    C
  27    9    1    1
Selective dynamics
Direct
 0.0025430854898781 0.0068150690459965 0.0113952143723907  T  T  T
 0.1080483860205068 0.6470134426602877 0.9729641767265718  T  T  T
 0.8836774686241760 0.2910135893752829 0.9472615523899383  T  T  T
 0.3367745234844401 -0.0031297661332219 0.0027899215208698  T  T  T
 0.4384597954701307 0.6441341773833191 0.9733806120571524  T  T  T
 0.2125797461302139 0.2913417790660792 0.9464878573850043  T  T  T
 0.6619453810067182 -0.0055265248312617 0.0029355333869881  T  T  T
 0.7772627686621361 0.6469351009053756 0.9738535726935595  T  T  T
 0.5505944209382195 0.2980768771349451 0.9486425785147100  T  T  T
 0.9649129391594045 0.7894776349564054 0.7810599999999965  F  F  F
 0.0701768413975614 0.4210610483853827 0.7445680000000010  F  F  F
 0.8421020476201875 0.0526265116863485 0.7080800000000025  F  F  F
 0.9473680488809180 0.6842082932855078 0.6715879999999999  F  F  F
 0.2982510144811457 0.7894776349564054 0.7810599999999965  F  F  F
 0.4035149167193097 0.4210610483853827 0.7445680000000010  F  F  F
 0.1754401229419287 0.0526265116863485 0.7080800000000025  F  F  F
 0.2807061242026592 0.6842082932855078 0.6715879999999999  F  F  F
 0.6315772348318589 0.7894776349564054 0.7810599999999965  F  F  F
 0.7368411370700230 0.4210610483853827 0.7445680000000010  F  F  F
 0.5087663432926490 0.0526265116863485 0.7080800000000025  F  F  F
 0.6140323445533795 0.6842082932855078 0.6715879999999999  F  F  F
 0.0526319511190820 0.3157917067144922 0.6350959999999972  F  F  F
 0.7192938757973408 0.3157917067144922 0.6350959999999972  F  F  F
 0.8245598770580713 0.9473734883136515 0.5986079999999987  F  F  F
 0.1578979523798125 0.9473734883136515 0.5986079999999987  F  F  F
 0.3859700264408232 0.3157917067144922 0.6350959999999972  F  F  F
 0.4912241727305329 0.9473734883136515 0.5986079999999987  F  F  F
 0.9871516380130770 0.9225995984414536 0.8981806652515800  T  T  T
 0.0883416630679534 0.5343096810878976 0.8608469422746209  T  T  T
 0.3201651722026447 0.9173604817197576 0.8939933265541171  T  T  T
 0.4218445144928718 0.5301314325395471 0.8607516146720403  T  T  T
 0.6522790422065998 0.9147339519102664 0.8943271565451660  T  T  T
 0.7563925875758819 0.5337653477582628 0.8612075690220415  T  T  T
 0.8613334051823848 0.1650685405919741 0.8206330281289511  T  T  T
 0.1931351934216501 0.1643049410064644 0.8201973034942590  T  T  T
 0.5282471085091662 0.1656816194010139 0.8197827617365453  T  T  T
 -0.0155785333284068 -0.0832425029352472  0.1300354215683539  T  T  T
 -0.0099454935643368 -0.0553164707814187  0.0841215605540290  T  T  T
Surface(331)-4 layers
  1.00000000000000
    8.4352800000000006    0.0000000000000000    0.0000000000000000
   -1.4058800000000000    6.1280900000000003    0.0000000000000000
    0.0000000000000000    0.0000000000000000   25.0000000000000000
  Pt   Cd   O    C
  27    9    1    1
Selective dynamics
Direct
 0.0474034235424253 0.2836962246242234 0.0400031911153440  T  T  T
 0.1547730082201148 0.9290490759416707 0.9947516068025265  T  T  T
 0.9292185922324316 0.5679800507812768 0.9742327249186914  T  T  T
 0.3822123577271428 0.2739304736021274 0.0322414698449888  T  T  T
 0.4870292660731616 0.9270253028457623 0.9963925576945499  T  T  T
 0.2590571210035043 0.5677074791285914 0.9729692850496523  T  T  T
 0.7079590377466235 0.2732338967539470 0.0323433950643747  T  T  T
 0.8208221844257872 0.9279785831652383 0.9944597642768808  T  T  T
 0.5936252314544677 0.5699869231080276 0.9714181894363643  T  T  T
 0.9649129391594045 0.7894776349564054 0.7810599999999965  F  F  F
 0.0701768413975614 0.4210610483853827 0.7445680000000010  F  F  F
 0.8421020476201875 0.0526265116863485 0.7080800000000025  F  F  F
 0.9473680488809180 0.6842082932855078 0.6715879999999999  F  F  F
 0.2982510144811457 0.7894776349564054 0.7810599999999965  F  F  F
 0.4035149167193097 0.4210610483853827 0.7445680000000010  F  F  F
 0.1754401229419287 0.0526265116863485 0.7080800000000025  F  F  F
 0.2807061242026592 0.6842082932855078 0.6715879999999999  F  F  F
 0.6315772348318589 0.7894776349564054 0.7810599999999965  F  F  F
 0.7368411370700230 0.4210610483853827 0.7445680000000010  F  F  F
 0.5087663432926490 0.0526265116863485 0.7080800000000025  F  F  F
 0.6140323445533795 0.6842082932855078 0.6715879999999999  F  F  F
 0.0526319511190820 0.3157917067144922 0.6350959999999972  F  F  F
 0.7192938757973408 0.3157917067144922 0.6350959999999972  F  F  F
 0.8245598770580713 0.9473734883136515 0.5986079999999987  F  F  F
 0.1578979523798125 0.9473734883136515 0.5986079999999987  F  F  F
 0.3859700264408232 0.3157917067144922 0.6350959999999972  F  F  F
 0.4912241727305329 0.9473734883136515 0.5986079999999987  F  F  F
 0.0343173813832348 0.2107501250587787 0.9238873647981858  T  T  T
 0.1175237188034709 0.7074894574526973 0.8782545179076450  T  T  T
 0.3661699766117602 0.1988211347954018 0.9224278871292099  T  T  T
 0.4458060546210361 0.6751878260443093 0.8746043129279719  T  T  T
```

```
0.7000139345729021 0.2041508278911834 0.9223054636209124 T T T
0.7850229797652925 0.7136752559794508 0.8785778892619212 T T T
0.8627819651280315 0.1758767652969879 0.8201908476386033 T T T
0.1948490145873032 0.1731825168279411 0.8199635992909234 T T T
0.5285421283352005 0.1702857322764819 0.8198536374763977 T T T
0.0129401774293711 0.0705442576371453 0.1487710444460139 T T T
0.0268365130377585 0.1530646133987944 0.1070337166158891 T T T
Surface(331)-4 layers
1.00000000000000
8.4352800000000006 0.0000000000000000 0.0000000000000000
-1.4058800000000000 6.1280900000000003 0.0000000000000000
0.0000000000000000 0.0000000000000000 25.0000000000000000
Pt Ta O C
27 9 1 1
Selective dynamics
Direct
0.0031825422946009 0.0195538706802287 0.0101211924151048 T T T
0.1089336491213467 0.6642123011334268 0.9642953842679284 T T T
0.8841175024044015 0.2956131683562317 0.9282392274204121 T T T
0.3387168123563288 0.0204015239997077 0.0009729514111149 T T T
0.4433184286599682 0.6599074183217728 0.9647891744506747 T T T
0.2155135027114269 0.2952607746949996 0.9283731785307977 T T T
0.6682681494712590 0.0213864750372759 0.0010499624404166 T T T
0.7792579827643947 0.6642980259691722 0.9639055638004121 T T T
0.5485581883446787 0.2996168822971619 0.9269294878160269 T T T
0.9649129391594045 0.7894776349564054 0.7810599999999965 F F F
0.0701768413975614 0.4210610483853827 0.7445680000000010 F F F
0.8421020476201875 0.0526265116863485 0.7080800000000025 F F F
0.9473680488809180 0.6842082932855078 0.6715879999999999 F F F
0.2982510144811457 0.7894776349564054 0.7810599999999965 F F F
0.4035149167193097 0.4210610483853827 0.7445680000000010 F F F
0.1754401229419287 0.0526265116863485 0.7080800000000025 F F F
0.2807061242026592 0.6842082932855078 0.6715879999999999 F F F
0.6315772348318589 0.7894776349564054 0.7810599999999965 F F F
0.7368411370700230 0.4210610483853827 0.7445680000000010 F F F
0.5087663432926490 0.0526265116863485 0.7080800000000025 F F F
0.6140323445533795 0.6842082932855078 0.6715879999999999 F F F
0.0526319511190820 0.3157917067144922 0.6350959999999972 F F F
0.7192938757973408 0.3157917067144922 0.6350959999999972 F F F
0.8245598770580713 0.9473734883136515 0.5986079999999987 F F F
0.1578979523798125 0.9473734883136515 0.5986079999999987 F F F
0.3859700264408232 0.3157917067144922 0.6350959999999972 F F F
0.4912241727305329 0.9473734883136515 0.5986079999999987 F F F
0.9890952816007071 0.9330838016018379 0.8988571819574996 T T T
0.0895298205664356 0.5350334716116804 0.8549789519652727 T T T
0.3223006394953287 0.9329457116091692 0.8997197446513904 T T T
0.4230278640290137 0.5385303932771678 0.8544304717471815 T T T
0.6558427702790195 0.9339616485105586 0.8997696247353325 T T T
0.7562316124983153 0.5374246343310483 0.8544649243377891 T T T
0.8579641478579385 0.1491664755204524 0.8114679728563289 T T T
0.1915833147888576 0.1490406986055892 0.8114570611782372 T T T
0.5249411076147472 0.1491238561037447 0.8116609963451158 T T T
0.0030200956503647 0.0193256378708036 0.1325918589925879 T T T
0.0050367308350083 0.0314784194735252 0.0862222477604037 T T T
Surface(331)-4 layers
1.00000000000000
8.4352800000000006 0.0000000000000000 0.0000000000000000
-1.4058800000000000 6.1280900000000003 0.0000000000000000
0.0000000000000000 0.0000000000000000 25.0000000000000000
Pt W O C
27 9 1 1
Selective dynamics
Direct
-0.0005325667858394 -0.0016445695482534 -0.0010930139257725 T T T
0.1053086571740530 0.6385956856677546 0.9572591902511290 T T T
0.8798992760755022 0.2719777532512835 0.9209287770716593 T T T
0.3352859270850462 -0.0023485485756213 -0.0082759577324663 T T T
0.4383144510380367 0.6337802430196608 0.9571834252491332 T T T
0.2105426267805678 0.2704412751431869 0.9208949494853258 T T T
0.6638484801835602 -0.0026813989836796 -0.0081583420867792 T T T
0.7740253790554437 0.6383715881405987 0.9569193134369751 T T T
0.5453725028333903 0.2741542292208594 0.9200035962489067 T T T
0.9649129391594045 0.7894776349564054 0.7810599999999965 F F F
0.0701768413975614 0.4210610483853827 0.7445680000000010 F F F
0.8421020476201875 0.0526265116863485 0.7080800000000025 F F F
0.9473680488809180 0.6842082932855078 0.6715879999999999 F F F
0.2982510144811457 0.7894776349564054 0.7810599999999965 F F F
0.4035149167193097 0.4210610483853827 0.7445680000000010 F F F
0.1754401229419287 0.0526265116863485 0.7080800000000025 F F F
0.2807061242026592 0.6842082932855078 0.6715879999999999 F F F
0.6315772348318589 0.7894776349564054 0.7810599999999965 F F F
0.7368411370700230 0.4210610483853827 0.7445680000000010 F F F
0.5087663432926490 0.0526265116863485 0.7080800000000025 F F F
0.6140323445533795 0.6842082932855078 0.6715879999999999 F F F
0.0526319511190820 0.3157917067144922 0.6350959999999972 F F F
0.7192938757973408 0.3157917067144922 0.6350959999999972 F F F
0.8245598770580713 0.9473734883136515 0.5986079999999987 F F F
0.1578979523798125 0.9473734883136515 0.5986079999999987 F F F
0.3859700264408232 0.3157917067144922 0.6350959999999972 F F F
0.4912241727305329 0.9473734883136515 0.5986079999999987 F F F
0.9843163781208691 0.9077854608721305 0.8899505194897256 T T T
0.0878024011365455 0.5258877433771693 0.8514445324403991 T T T
0.3181498616906896 0.9080756437371308 0.8908238052073387 T T T
0.4217797734100570 0.5267343244295467 0.8506729995163914 T T T
0.6512161906082335 0.9086820738289575 0.8910394537350126 T T T
0.7542300213134030 0.5273586011258281 0.8509070493361529 T T T
0.8585484341717766 0.1498983432916986 0.8111807065743346 T T T
0.1915708519928954 0.1498205805297776 0.8111543799913110 T T T
0.5248207061901181 0.1499110224048103 0.8112527086572821 T T T
0.0007607741966405 0.0046042227877709 0.1215322191326122 T T T
0.0021289624717057 0.0131976135507963 0.0751404359599290 T T T
Surface(331)-4 layers
1.00000000000000
8.4352800000000006 0.0000000000000000 0.0000000000000000
-1.4058800000000000 6.1280900000000003 0.0000000000000000
0.0000000000000000 0.0000000000000000 25.0000000000000000
Pt Re O C
27 9 1 1
Selective dynamics
Direct
-0.0024668002925838 -0.0099848349321187 -0.0065381485963999 T T T
0.1038077061389238 0.6293067237319706 0.9551462403382974 T T T
0.8782749183419605 0.2661264956696632 0.9190978613593320 T T T
0.3343555076228944 -0.0140426002810832 -0.0137219276601555 T T T
0.4379952162187145 0.6267747266229218 0.9538484169550083 T T T
0.2099272998198480 0.2653223825226582 0.9192165215315803 T T T
0.6610013124204583 -0.0127740397731143 -0.0133767083543060 T T T
0.7712506806758491 0.6281808091198841 0.9547498197290226 T T T
0.5431829260113239 0.2647009122207270 0.9178171859451447 T T T
0.9649129391594045 0.7894776349564054 0.7810599999999965 F F F
0.0701768413975614 0.4210610483853827 0.7445680000000010 F F F
0.8421020476201875 0.0526265116863485 0.7080800000000025 F F F
0.9473680488809180 0.6842082932855078 0.6715879999999999 F F F
0.2982510144811457 0.7894776349564054 0.7810599999999965 F F F
0.4035149167193097 0.4210610483853827 0.7445680000000010 F F F
0.1754401229419287 0.0526265116863485 0.7080800000000025 F F F
0.2807061242026592 0.6842082932855078 0.6715879999999999 F F F
0.6315772348318589 0.7894776349564054 0.7810599999999965 F F F
0.7368411370700230 0.4210610483853827 0.7445680000000010 F F F
0.5087663432926490 0.0526265116863485 0.7080800000000025 F F F
0.6140323445533795 0.6842082932855078 0.6715879999999999 F F F
0.0526319511190820 0.3157917067144922 0.6350959999999972 F F F
0.7192938757973408 0.3157917067144922 0.6350959999999972 F F F
0.8245598770580713 0.9473734883136515 0.5986079999999987 F F F
0.1578979523798125 0.9473734883136515 0.5986079999999987 F F F
0.3859700264408232 0.3157917067144922 0.6350959999999972 F F F
0.4912241727305329 0.9473734883136515 0.5986079999999987 F F F
0.9805031927028034 0.8964941107611449 0.8865542727510206 T T T
0.0872696285816143 0.5268920614228275 0.8498735723805312 T T T
0.3034071132062302 0.8951581658911206 0.8836711052785156 T T T
0.4201454079596089 0.5199961025049312 0.8482613858466193 T T T
0.6605898139291480 0.8983247871844161 0.8842433663034033 T T T
0.7534532291667340 0.5258047477231552 0.8488078340087890 T T T
0.8520906141343629 0.1559994113606233 0.8146057762662697 T T T
0.2025864489262121 0.1569664075866400 0.8150000382729184 T T T
0.5261387839468663 0.1521916096493467 0.8128621250503358 T T T
0.0017434984036758 0.0102829866781084 0.1156037771669278 T T T
0.0022677510197251 0.0137999817354480 0.0691463920187831 T T T
Surface(331)-4 layers
1.00000000000000
8.4352800000000006 0.0000000000000000 0.0000000000000000
-1.4058800000000000 6.1280900000000003 0.0000000000000000
0.0000000000000000 0.0000000000000000 25.0000000000000000
Pt Os O C
27 9 1 1
Selective dynamics
Direct
-0.0019317773766819 -0.0106536254582567 -0.0085000066893664 T T T
0.1048345134212128 0.6329299601406412 0.9540421532708917 T T T
0.8788827119473667 0.2701664531919841 0.9198957602868432 T T T
0.3345559329242466 -0.0123286279053966 -0.0145913292391566 T T T
0.4375968667656056 0.6283022180399994 0.9546374910407043 T T T
0.2116115727020779 0.2683284199565074 0.9198612639667794 T T T
0.6614467213274950 -0.0119755644862558 -0.0145934856827830 T T T
0.7733013415393223 0.6326464300265680 0.9538719069565976 T T T
0.5447664258242886 0.2718778822573202 0.9193203542704698 T T T
0.9649129391594045 0.7894776349564054 0.7810599999999965 F F F
0.0701768413975614 0.4210610483853827 0.7445680000000010 F F F
0.8421020476201875 0.0526265116863485 0.7080800000000025 F F F
0.9473680488809180 0.6842082932855078 0.6715879999999999 F F F
0.2982510144811457 0.7894776349564054 0.7810599999999965 F F F
0.4035149167193097 0.4210610483853827 0.7445680000000010 F F F
0.1754401229419287 0.0526265116863485 0.7080800000000025 F F F
0.2807061242026592 0.6842082932855078 0.6715879999999999 F F F
0.6315772348318589 0.7894776349564054 0.7810599999999965 F F F
0.7368411370700230 0.4210610483853827 0.7445680000000010 F F F
0.5087663432926490 0.0526265116863485 0.7080800000000025 F F F
0.6140323445533795 0.6842082932855078 0.6715879999999999 F F F
0.0526319511190820 0.3157917067144922 0.6350959999999972 F F F
0.7192938757973408 0.3157917067144922 0.6350959999999972 F F F
0.8245598770580713 0.9473734883136515 0.5986079999999987 F F F
0.1578979523798125 0.9473734883136515 0.5986079999999987 F F F
0.3859700264408232 0.3157917067144922 0.6350959999999972 F F F
0.4912241727305329 0.9473734883136515 0.5986079999999987 F F F
0.9842052683524958 0.9068211121413878 0.8835981578871471 T T T
0.0883787775428287 0.5244887386188679 0.8486600359797737 T T T
0.3169272559337054 0.9017971964291760 0.8818010086416032 T T T
0.4212590084399412 0.5255546707762954 0.8483803701868949 T T T
0.6507568533805144 0.9024559305997709 0.8818983322220640 T T T
0.7529117470853441 0.5251907004905489 0.8484599230315016 T T T
0.8598738919793674 0.1571757481506726 0.8168958711082916 T T T
0.1918341596806361 0.1567059303476052 0.8167915212223482 T T T
0.5266108564585679 0.1569666325536224 0.8164260649596669 T T T
0.0012991549109080 0.0089085523238261 0.1130663500545088 T T T
0.0016286974426319 0.0111094475629267 0.0665983187449168 T T T
Surface(331)-4 layers
1.00000000000000
8.4352800000000006 0.0000000000000000 0.0000000000000000
-1.4058800000000000 6.1280900000000003 0.0000000000000000
0.0000000000000000 0.0000000000000000 25.0000000000000000
Pt Ir O C
27 9 1 1
Selective dynamics
Direct
-0.0018477935492995 -0.0105786752824383 -0.0068019102947024 T T T
0.1057095540645498 0.6340591018075957 0.9555897833812227 T T T
0.8795706467222936 0.2726850318616987 0.9237646205367080 T T T
0.3356537416117570 -0.0114996773203117 -0.0131305858204668 T T T
0.4378609133690429 0.6295665164500222 0.9563389230337390 T T T
0.2117966911128175 0.2702865659119722 0.9235684816291315 T T T
0.6612767421451720 -0.0108992801382226 -0.0131054634794067 T T T
0.7733529188681139 0.6337051246480742 0.9554158635273035 T T T
0.5451415262875523 0.2738868667581917 0.9226266438027785 T T T
0.9649129391594045 0.7894776349564054 0.7810599999999965 F F F
0.0701768413975614 0.4210610483853827 0.7445680000000010 F F F
0.8421020476201875 0.0526265116863485 0.7080800000000025 F F F
0.9473680488809180 0.6842082932855078 0.6715879999999999 F F F
0.2982510144811457 0.7894776349564054 0.7810599999999965 F F F
0.4035149167193097 0.4210610483853827 0.7445680000000010 F F F
0.1754401229419287 0.0526265116863485 0.7080800000000025 F F F
0.2807061242026592 0.6842082932855078 0.6715879999999999 F F F
0.6315772348318589 0.7894776349564054 0.7810599999999965 F F F
0.7368411370700230 0.4210610483853827 0.7445680000000010 F F F
0.5087663432926490 0.0526265116863485 0.7080800000000025 F F F
0.6140323445533795 0.6842082932855078 0.6715879999999999 F F F
0.0526319511190820 0.3157917067144922 0.6350959999999972 F F F
0.7192938757973408 0.3157917067144922 0.6350959999999972 F F F
0.8245598770580713 0.9473734883136515 0.5986079999999987 F F F
0.1578979523798125 0.9473734883136515 0.5986079999999987 F F F
0.3859700264408232 0.3157917067144922 0.6350959999999972 F F F
0.4912241727305329 0.9473734883136515 0.5986079999999987 F F F
0.9842124648761373 0.9064958511232517 0.8860160933941286 T T T
0.0869731036530040 0.5240241633443732 0.8497343157270185 T T T
0.3170286068016137 0.8995390236253912 0.8826838032030189 T T T
0.4206718781320683 0.5238039096891894 0.8495484673755846 T T T
0.6499328447403989 0.9006085969558277 0.8828286252640030 T T T
0.7540808220239726 0.5245925873437506 0.8494780736688842 T T T
0.8610754970066617 0.1597789469135739 0.8164259237874572 T T T
0.1920494123584989 0.1588185471281571 0.8163446198057176 T T T
0.5266446594069168 0.1600268351050157 0.8159090326595467 T T T
0.0013802504649360 0.0086481942472439 0.1142016778526689 T T T
0.0013846064658772 0.0090083700904496 0.0677279469826862 T T T
Surface(331)-4 layers
1.00000000000000
8.4352800000000006 0.0000000000000000 0.0000000000000000
-1.4058800000000000 6.1280900000000003 0.0000000000000000
0.0000000000000000 0.0000000000000000 25.0000000000000000
Pt O C
36 1 1
Selective dynamics
Direct
-0.0032782121539319 -0.0168043253970884 -0.0017585165620868 T T T
0.1048294698051121 0.6246142086476171 0.9639513417037749 T T T
0.8788169348267376 0.2720985897551871 0.9316272996925580 T T T
0.3342562422301213 -0.0185400677325883 -0.0078908584770840 T T T
0.4364133902107435 0.6222192736611356 0.9638003569235911 T T T
0.2110945523230699 0.2703521878078830 0.9307440624900239 T T T
0.6586169906460869 -0.0186224429893350 -0.0077950034197832 T T T
0.7693724778256595 0.6239085140245179 0.9636462362639121 T T T
0.5445154943798051 0.2728653144570691 0.9299017219194585 T T T
0.9649129391594045 0.7894776349564054 0.7810599999999965 F F F
0.0701768413975614 0.4210610483853827 0.7445680000000010 F F F
0.8421020476201875 0.0526265116863485 0.7080800000000025 F F F
0.9473680488809180 0.6842082932855078 0.6715879999999999 F F F
0.2982510144811457 0.7894776349564054 0.7810599999999965 F F F
0.4035149167193097 0.4210610483853827 0.7445680000000010 F F F
0.1754401229419287 0.0526265116863485 0.7080800000000025 F F F
0.2807061242026592 0.6842082932855078 0.6715879999999999 F F F
0.6315772348318589 0.7894776349564054 0.7810599999999965 F F F
0.7368411370700230 0.4210610483853827 0.7445680000000010 F F F
0.5087663432926490 0.0526265116863485 0.7080800000000025 F F F
0.6140323445533795 0.6842082932855078 0.6715879999999999 F F F
0.0526319511190820 0.3157917067144922 0.6350959999999972 F F F
0.7192938757973408 0.3157917067144922 0.6350959999999972 F F F
0.8245598770580713 0.9473734883136515 0.5986079999999987 F F F
0.1578979523798125 0.9473734883136515 0.5986079999999987 F F F
0.3859700264408232 0.3157917067144922 0.6350959999999972 F F F
0.4912241727305329 0.9473734883136515 0.5986079999999987 F F F
0.9832660936749583 0.9036596424407863 0.8921542808081924 T T T
0.0866398878394771 0.5281217624428914 0.8544658824938668 T T T
0.3168785349721765 0.8993805116180981 0.8867192731622441 T T T
0.4206102623476646 0.5245579901857241 0.8538126690994906 T T T
0.6497175350827644 0.9010196220169661 0.8868385994586204 T T T
0.7557331991173406 0.5284144046945549 0.8540105138659138 T T T
0.8616963573230676 0.1616453497802552 0.8173061391061398 T T T
0.1912534716247917 0.1604412296321091 0.8171373730401724 T T T
0.5272015785975179 0.1639327491507730 0.8163319607384215 T T T
0.0010879389629982 0.0071687443538208 0.1187423785849286 T T T
0.0000077008925982 0.0021503821822815 0.0723297486815686 T T T
Surface(331)-4 layers
1.00000000000000
8.4352800000000006 0.0000000000000000 0.0000000000000000
-1.4058800000000000 6.1280900000000003 0.0000000000000000
0.0000000000000000 0.0000000000000000 25.0000000000000000
Pt Au O C
27 9 1 1
Selective dynamics
Direct
0.0026125901953104 0.0098689679786675 0.0137789712010518 T T T
0.1093844361521744 0.6495348010109444 0.9770542153064581 T T T
0.8838947654645308 0.2982944497043197 0.9488170580642766 T T T
0.3370971646945528 0.0020356763889344 0.0067087086357566 T T T
0.4405904801475835 0.6488966618685688 0.9773232036895217 T T T
0.2153567031121548 0.2974510589927278 0.9478901154121632 T T T
0.6639142120275461 0.0013178471057144 0.0070236521400801 T T T
0.7755377283371462 0.6486064230950417 0.9780524527552975 T T T
0.5505342904754141 0.3002955749522958 0.9498618971466466 T T T
0.9649129391594045 0.7894776349564054 0.7810599999999965 F F F
0.0701768413975614 0.4210610483853827 0.7445680000000010 F F F
0.8421020476201875 0.0526265116863485 0.7080800000000025 F F F
0.9473680488809180 0.6842082932855078 0.6715879999999999 F F F
0.2982510144811457 0.7894776349564054 0.7810599999999965 F F F
0.4035149167193097 0.4210610483853827 0.7445680000000010 F F F
0.1754401229419287 0.0526265116863485 0.7080800000000025 F F F
0.2807061242026592 0.6842082932855078 0.6715879999999999 F F F
0.6315772348318589 0.7894776349564054 0.7810599999999965 F F F
0.7368411370700230 0.4210610483853827 0.7445680000000010 F F F
0.5087663432926490 0.0526265116863485 0.7080800000000025 F F F
0.6140323445533795 0.6842082932855078 0.6715879999999999 F F F
0.0526319511190820 0.3157917067144922 0.6350959999999972 F F F
0.7192938757973408 0.3157917067144922 0.6350959999999972 F F F
0.8245598770580713 0.9473734883136515 0.5986079999999987 F F F
0.1578979523798125 0.9473734883136515 0.5986079999999987 F F F
0.3859700264408232 0.3157917067144922 0.6350959999999972 F F F
0.4912241727305329 0.9473734883136515 0.5986079999999987 F F F
0.9878990965604042 0.9291916210936410 0.9028546441352766 T T T
0.0888586651995564 0.5404793107382900 0.8624651286361057 T T T
0.3212436042252025 0.9243732433219302 0.8970194369775022 T T T
0.4232084251061474 0.5371332290497507 0.8622447176160803 T T T
0.6534851641396145 0.9240440825733391 0.8974457698722847 T T T
0.7578717475490470 0.5407400472969739 0.8627540976782606 T T T
0.8619025522008497 0.1660661755185122 0.8209327179931749 T T T
0.1930182039702532 0.1658325474718349 0.8206853193747322 T T T
0.5286086251636040 0.1685342598126592 0.8199366640622985 T T T
-0.0043505911582991 -0.0223066334696988 0.1340102188436749 T T T
-0.0025402666383725 -0.0139845483226143 0.0876260418329654 T T T
Surface(331)-4 layers
1.00000000000000
8.4352800000000006 0.0000000000000000 0.0000000000000000
-1.4058800000000000 6.1280900000000003 0.0000000000000000
0.0000000000000000 0.0000000000000000 25.0000000000000000
Pt Hg O C
27 9 1 1
Selective dynamics
Direct
0.0458618509959426 0.2735453207808687 0.0468355331882490 T T T
0.1537213463220615 0.9157379631753459 0.0050124632160696 T T T
0.9279546255809091 0.5589490061868094 0.9808590356114180 T T T
0.3819967326263565 0.2673794436186812 0.0383664124720920 T T T
0.4841180099446880 0.9129139835085937 0.0068027748137255 T T T
0.2564926719138119 0.5588193625788480 0.9801105624976212 T T T
0.7056235049764640 0.2655244206446856 0.0383473743952751 T T T
0.8182126799948072 0.9137491182743369 0.0059620216254393 T T T
0.5938819312518360 0.5632772331981246 0.9777124482547098 T T T
0.9649129391594045 0.7894776349564054 0.7810599999999965 F F F
0.0701768413975614 0.4210610483853827 0.7445680000000010 F F F
0.8421020476201875 0.0526265116863485 0.7080800000000025 F F F
0.9473680488809180 0.6842082932855078 0.6715879999999999 F F F
0.2982510144811457 0.7894776349564054 0.7810599999999965 F F F
0.4035149167193097 0.4210610483853827 0.7445680000000010 F F F
0.1754401229419287 0.0526265116863485 0.7080800000000025 F F F
0.2807061242026592 0.6842082932855078 0.6715879999999999 F F F
0.6315772348318589 0.7894776349564054 0.7810599999999965 F F F
0.7368411370700230 0.4210610483853827 0.7445680000000010 F F F
0.5087663432926490 0.0526265116863485 0.7080800000000025 F F F
0.6140323445533795 0.6842082932855078 0.6715879999999999 F F F
0.0526319511190820 0.3157917067144922 0.6350959999999972 F F F
0.7192938757973408 0.3157917067144922 0.6350959999999972 F F F
0.8245598770580713 0.9473734883136515 0.5986079999999987 F F F
0.1578979523798125 0.9473734883136515 0.5986079999999987 F F F
0.3859700264408232 0.3157917067144922 0.6350959999999972 F F F
0.4912241727305329 0.9473734883136515 0.5986079999999987 F F F
0.0310000584244799 0.1931310145532588 0.9295387305946099 T T T
0.1149686639974763 0.6914664833250909 0.8803207472610647 T T T
0.3637424902069988 0.1852968609324974 0.9265958677507922 T T T
0.4461674035203228 0.6779842030984164 0.8782591885558440 T T T
0.6958806075951646 0.1776685285150386 0.9268572864027353 T T T
0.7808363296187001 0.6828672217817950 0.8790207442693020 T T T
0.8635534663547905 0.1779915653410620 0.8234560103711955 T T T
0.1954202733508058 0.1776935660990245 0.8231764109369376 T T T
0.5292787836312935 0.1754440087236324 0.8226642839222602 T T T
1.0065151906727416 1.0349239541782214 0.1523437637606297 T T T
0.0218283719246011 0.1275369331059627 0.1119171107581533 T T T
```

331 *OH

```
Surface(331)-4 layers
1.00000000000000
8.4352800000000006 0.0000000000000000 0.0000000000000000
-1.4058800000000000 6.1280900000000003 0.0000000000000000
0.0000000000000000 0.0000000000000000 25.0000000000000000
```

Pt V O H
27 9 1 1
Selective dynamics
Direct
0.0003943994618421 0.0031096818099245 0.9846546971855397 T T T
0.1059186023472254 0.6413609516237023 0.9452200008872186 T T T
0.8804742173529494 0.2708118494917708 0.9064906265675858 T T T
0.3360607959328662 0.0003282061428858 0.9796127684626262 T T T
0.4388716856098350 0.6338388493123990 0.9454792219018542 T T T
0.2087439506139102 0.2691664503208608 0.9067027347388863 T T T
0.6644695989126317 0.0006837086802860 0.9796257492846673 T T T
0.7747408342361942 0.6411086463083462 0.9451147693027501 T T T
0.5467020040900000 0.2740790123390762 0.9057003545527301 T T T
0.9649129391594045 0.7894776349564054 0.7810599999999965 F F F
0.0701768413975614 0.4210610483853827 0.7445680000000010 F F F
0.8421020476201875 0.0526265116863485 0.7080800000000025 F F F
0.9473680488809180 0.6842082932855078 0.6715879999999999 F F F
0.2982510144811457 0.7894776349564054 0.7810599999999965 F F F
0.4035149167193097 0.4210610483853827 0.7445680000000010 F F F
0.1754401229419287 0.0526265116863485 0.7080800000000025 F F F
0.2807061242026592 0.6842082932855078 0.6715879999999999 F F F
0.6315772348318589 0.7894776349564054 0.7810599999999965 F F F
0.7368411370700230 0.4210610483853827 0.7445680000000010 F F F
0.5087663432926490 0.0526265116863485 0.7080800000000025 F F F
0.6140323445533795 0.6842082932855078 0.6715879999999999 F F F
0.0526319511190820 0.3157917067144922 0.6350959999999972 F F F
0.7192938757973408 0.3157917067144922 0.6350959999999972 F F F
0.8245598770580713 0.9473734883136515 0.5986079999999987 F F F
0.1578979523798125 0.9473734883136515 0.5986079999999987 F F F
0.3859700264408232 0.3157917067144922 0.6350959999999972 F F F
0.4912241727305329 0.9473734883136515 0.5986079999999987 F F F
0.9846591499876748 0.9094255906251455 0.8850492498410073 T T T
0.0917569473501132 0.5408259463554139 0.8457108004891006 T T T
0.3204975408994160 0.9070598800217771 0.8847461517732527 T T T
0.4215967163104545 0.5408635714543569 0.8455650858841380 T T T
0.6490313360631097 0.9068496207026222 0.8848009980431703 T T T
0.7577824787054729 0.5420949920891666 0.8454590467194681 T T T
0.8573245192063879 0.1403236264563218 0.8066980867049642 T T T
0.1894911017112182 0.1413257926159298 0.8066497033845786 T T T
0.5236854578341728 0.1414965855559949 0.8064040808934356 T T T
0.0010322389800822 0.0136242165977804 0.0664531913042308 T T T
0.0235896901957020 0.1636235037745777 0.0796319536426544 T T T
Surface(331)-4 layers
1.00000000000000
8.4352800000000006 0.0000000000000000 0.0000000000000000
-1.4058800000000000 6.1280900000000003 0.0000000000000000
0.0000000000000000 0.0000000000000000 25.0000000000000000
Pt Cr O H
27 9 1 1
Selective dynamics
Direct
0.0052209956254445 0.0267966597757050 -0.0200472472308144 T T T
0.1109010810453101 0.6816153749227660 0.9336387050032715 T T T
0.8831883225457579 0.2922629554869603 0.9096496072567517 T T T
0.3390086663000133 0.0214898385683010 -0.0272100675391341 T T T
0.4436007674211564 0.6781149505929104 0.9307299672677929 T T T
0.2108581846937680 0.2993186597107029 0.9068940155826820 T T T
0.6633208604519591 0.0164213315751475 -0.0260835550806212 T T T
0.7819166096248769 0.6822558527215253 0.9335353917808318 T T T
0.5541066669905992 0.3112523186347405 0.9033751658409372 T T T
0.9649129391594045 0.7894776349564054 0.7810599999999965 F F F
0.0701768413975614 0.4210610483853827 0.7445680000000010 F F F
0.8421020476201875 0.0526265116863485 0.7080800000000025 F F F
0.9473680488809180 0.6842082932855078 0.6715879999999999 F F F
0.2982510144811457 0.7894776349564054 0.7810599999999965 F F F
0.4035149167193097 0.4210610483853827 0.7445680000000010 F F F
0.1754401229419287 0.0526265116863485 0.7080800000000025 F F F
0.2807061242026592 0.6842082932855078 0.6715879999999999 F F F
0.6315772348318589 0.7894776349564054 0.7810599999999965 F F F
0.7368411370700230 0.4210610483853827 0.7445680000000010 F F F
0.5087663432926490 0.0526265116863485 0.7080800000000025 F F F
0.6140323445533795 0.6842082932855078 0.6715879999999999 F F F
0.0526319511190820 0.3157917067144922 0.6350959999999972 F F F
0.7192938757973408 0.3157917067144922 0.6350959999999972 F F F
0.8245598770580713 0.9473734883136515 0.5986079999999987 F F F
0.1578979523798125 0.9473734883136515 0.5986079999999987 F F F
0.3859700264408232 0.3157917067144922 0.6350959999999972 F F F
0.4912241727305329 0.9473734883136515 0.5986079999999987 F F F
0.9943234858136260 0.9725304658901932 0.8754234899883759 T T T
0.0679107024939034 0.5189986445647122 0.8410323077770068 T T T
0.3313684996694679 0.9827354012621334 0.8737679524612790 T T T
0.4022120095152900 0.5090682629774839 0.8386882990982425 T T T
0.6621853401134522 0.9777165743326359 0.8742769353717476 T T T
0.7815652433283238 0.5203497068776802 0.8402540384348647 T T T
0.8570578403459634 0.1400891540123491 0.8113352918660527 T T T
0.1884034857314374 0.1323400825581156 0.8105434525242272 T T T
0.5214122870834141 0.1269866035041982 0.8100104583200204 T T T
-0.0024779465758465 -0.0149719202579755 0.0599592758918144 T T T
0.0181601182300013 0.1286441206207297 0.0769878921059065 T T T
Surface(331)-4 layers
1.00000000000000
8.4352800000000006 0.0000000000000000 0.0000000000000000
-1.4058800000000000 6.1280900000000003 0.0000000000000000
0.0000000000000000 0.0000000000000000 25.0000000000000000
Pt Mn O H
27 9 1 1
Selective dynamics
Direct
-0.0014228205350591 0.0123841725692001 -0.0222815670057280 T T T
0.1096723390901094 0.6760656125980937 0.9296378855704357 T T T
0.8816885724433371 0.2917840661344362 0.9077510525440589 T T T
0.3369976874392221 0.0057446462620077 -0.0280879336078464 T T T
0.4445556099107352 0.6685854528096726 0.9295361664050890 T T T
0.2080417844792456 0.2823601206210330 0.9087066691004133 T T T
0.6640452835096355 0.0105495791101057 -0.0273796485638070 T T T
0.7789030955231033 0.6724517429749194 0.9298938773235345 T T T
0.5442241670085856 0.2964724411595238 0.9030613277844206 T T T
0.9649129391594045 0.7894776349564054 0.7810599999999965 F F F
0.0701768413975614 0.4210610483853827 0.7445680000000010 F F F
0.8421020476201875 0.0526265116863485 0.7080800000000025 F F F
0.9473680488809180 0.6842082932855078 0.6715879999999999 F F F
0.2982510144811457 0.7894776349564054 0.7810599999999965 F F F
0.4035149167193097 0.4210610483853827 0.7445680000000010 F F F
0.1754401229419287 0.0526265116863485 0.7080800000000025 F F F
0.2807061242026592 0.6842082932855078 0.6715879999999999 F F F
0.6315772348318589 0.7894776349564054 0.7810599999999965 F F F
0.7368411370700230 0.4210610483853827 0.7445680000000010 F F F
0.5087663432926490 0.0526265116863485 0.7080800000000025 F F F
0.6140323445533795 0.6842082932855078 0.6715879999999999 F F F
0.0526319511190820 0.3157917067144922 0.6350959999999972 F F F
0.7192938757973408 0.3157917067144922 0.6350959999999972 F F F
0.8245598770580713 0.9473734883136515 0.5986079999999987 F F F
0.1578979523798125 0.9473734883136515 0.5986079999999987 F F F
0.3859700264408232 0.3157917067144922 0.6350959999999972 F F F
0.4912241727305329 0.9473734883136515 0.5986079999999987 F F F
0.9813542243296584 0.9679556500902398 0.8746987125927683 T T T
0.0900695573797710 0.4965288514361736 0.8396198088166303 T T T
0.3231381209788994 0.9706684649204350 0.8722329174290501 T T T
0.3867688885299828 0.5000678683451354 0.8389199537708596 T T T
0.6663624360076082 0.9682731400375332 0.8741813731313633 T T T
0.7578162150081744 0.4930469822762689 0.8389355891328373 T T T
0.8645558072898754 0.1767073839019548 0.8108694455974603 T T T
0.2065415247895544 0.1614100226694920 0.8123742368929995 T T T
0.5574939063405711 0.1574172964423812 0.8096862659438476 T T T
-0.0012880912974208 -0.0125117699116867 0.0578996820447045 T T T
0.0195574695240890 0.1328465851693937 0.0739483419189638 T T T
Surface(331)-4 layers
1.00000000000000
8.4352800000000006 0.0000000000000000 0.0000000000000000
-1.4058800000000000 6.1280900000000003 0.0000000000000000
0.0000000000000000 0.0000000000000000 25.0000000000000000
Pt Fe O H
27 9 1 1
Selective dynamics
Direct
-0.0001184153012925 0.0051983558096587 -0.0241408327135755 T T T
0.1088486194723932 0.6646687953720792 0.9289072493255809 T T T
0.8811045915306178 0.2829277049502968 0.9069152035140237 T T T
0.3366440923404533 -0.0020700577288985 -0.0283742210531778 T T T
0.4416160576953246 0.6553969447113851 0.9310575288825385 T T T
0.2118558116852424 0.2742043993835114 0.9073209181251104 T T T
0.6637601212604713 0.0002671055739962 -0.0284630135134787 T T T
0.7797421589366543 0.6634252604057835 0.9300202824419997 T T T
0.5482921728077444 0.2919069171931816 0.9053199158781420 T T T
0.9649129391594045 0.7894776349564054 0.7810599999999965 F F F
0.0701768413975614 0.4210610483853827 0.7445680000000010 F F F
0.8421020476201875 0.0526265116863485 0.7080800000000025 F F F
0.9473680488809180 0.6842082932855078 0.6715879999999999 F F F
0.2982510144811457 0.7894776349564054 0.7810599999999965 F F F
0.4035149167193097 0.4210610483853827 0.7445680000000010 F F F
0.1754401229419287 0.0526265116863485 0.7080800000000025 F F F
0.2807061242026592 0.6842082932855078 0.6715879999999999 F F F
0.6315772348318589 0.7894776349564054 0.7810599999999965 F F F
0.7368411370700230 0.4210610483853827 0.7445680000000010 F F F
0.5087663432926490 0.0526265116863485 0.7080800000000025 F F F
0.6140323445533795 0.6842082932855078 0.6715879999999999 F F F
0.0526319511190820 0.3157917067144922 0.6350959999999972 F F F
0.7192938757973408 0.3157917067144922 0.6350959999999972 F F F
0.8245598770580713 0.9473734883136515 0.5986079999999987 F F F
0.1578979523798125 0.9473734883136515 0.5986079999999987 F F F
0.3859700264408232 0.3157917067144922 0.6350959999999972 F F F
0.4912241727305329 0.9473734883136515 0.5986079999999987 F F F
0.9876978081171666 0.9594082059441773 0.8738507878376892 T T T
0.0883031498582956 0.4881040507130244 0.8392668062497487 T T T
0.3346908116027465 0.9552746523466554 0.8729356611596395 T T T
0.4013763472749252 0.4920851719701513 0.8390771781189740 T T T
0.6574879586903918 0.9566933486566003 0.8732259237395968 T T T
0.7550710268338793 0.4922942961662409 0.8389621576485743 T T T
0.8609417920151448 0.1601246218285159 0.8093127737851760 T T T
0.1852359497771428 0.1576447332617014 0.8096174556580613 T T T
0.5341514926067251 0.1604951291603181 0.8088510231802596 T T T
0.0018674879549214 -0.0188779956787407 0.0558631495838539 T T T
0.0168869080003004 0.1265190812480429 0.0716570411288296 T T T
Surface(331)-4 layers
1.00000000000000
8.4352800000000006 0.0000000000000000 0.0000000000000000
-1.4058800000000000 6.1280900000000003 0.0000000000000000
0.0000000000000000 0.0000000000000000 25.0000000000000000
Pt Co O H
27 9 1 1
Selective dynamics
Direct
0.0009604604240778 0.0081082450062971 -0.0259874755121005 T T T
0.1088192956808445 0.6631430987485192 0.9309886451760812 T T T
0.8819254200757116 0.2876363998429695 0.9063336201024866 T T T
0.3373141191332336 0.0023834189888003 -0.0298058502902021 T T T
0.4419564549487686 0.6546166838783654 0.9334217323270373 T T T
0.2138053120117942 0.2855031788634636 0.9068866477968428 T T T
0.6637424413239992 0.0039952066507845 -0.0298399845772776 T T T
0.7796113546813715 0.6636380006801214 0.9311451444987530 T T T
0.5493494770524882 0.2953638822446550 0.9051518174785088 T T T
0.9649129391594045 0.7894776349564054 0.7810599999999965 F F F
0.0701768413975614 0.4210610483853827 0.7445680000000010 F F F
0.8421020476201875 0.0526265116863485 0.7080800000000025 F F F
0.9473680488809180 0.6842082932855078 0.6715879999999999 F F F
0.2982510144811457 0.7894776349564054 0.7810599999999965 F F F
0.4035149167193097 0.4210610483853827 0.7445680000000010 F F F
0.1754401229419287 0.0526265116863485 0.7080800000000025 F F F
0.2807061242026592 0.6842082932855078 0.6715879999999999 F F F
0.6315772348318589 0.7894776349564054 0.7810599999999965 F F F
0.7368411370700230 0.4210610483853827 0.7445680000000010 F F F
0.5087663432926490 0.0526265116863485 0.7080800000000025 F F F
0.6140323445533795 0.6842082932855078 0.6715879999999999 F F F
0.0526319511190820 0.3157917067144922 0.6350959999999972 F F F
0.7192938757973408 0.3157917067144922 0.6350959999999972 F F F
0.8245598770580713 0.9473734883136515 0.5986079999999987 F F F
0.1578979523798125 0.9473734883136515 0.5986079999999987 F F F
0.3859700264408232 0.3157917067144922 0.6350959999999972 F F F
0.4912241727305329 0.9473734883136515 0.5986079999999987 F F F
0.9916100193645826 0.9572008286530026 0.8730165650747819 T T T
0.0884808964888133 0.5125801771786792 0.8391035912777663 T T T
0.3296272834479946 0.9487785117378366 0.8720182352228618 T T T
0.4114136727993963 0.5172632287884942 0.8393554049445890 T T T
0.6550619063388254 0.9478781583397128 0.8721710628557512 T T T
0.7575099957544811 0.5134593268882122 0.8389732202859106 T T T
0.8596080563964503 0.1567975352030945 0.8081255053977063 T T T
0.1914335109195077 0.1566548093036451 0.8083233148795088 T T T
0.5283718700862156 0.1586666974608795 0.8075957002985213 T T T
-0.0009260786532305 -0.0203647590040193 0.0536882152779951 T T T
0.0184105237149008 0.1266607958543297 0.0687798207388318 T T T
Surface(331)-4 layers
1.00000000000000
8.4352800000000006 0.0000000000000000 0.0000000000000000
-1.4058800000000000 6.1280900000000003 0.0000000000000000
0.0000000000000000 0.0000000000000000 25.0000000000000000
Pt Ni O H
27 9 1 1
Selective dynamics
Direct
0.0018231234352599 0.0036648245816727 0.9761831568540492 T T T
0.1137124530514345 0.6617551606139728 0.9355663772491918 T T T
0.8868926513994144 0.2938019609057327 0.9071774392008916 T T T
0.3431783790189398 0.0055522633947638 0.9700385727255746 T T T
0.4455965528467299 0.6544099220303934 0.9369440873835795 T T T
0.2175648763349201 0.2921472098125145 0.9074697596788879 T T T
0.6682206334816611 0.0053706518169588 0.9705024816424707 T T T
0.7843970777436018 0.6628661118450216 0.9358887226645791 T T T
0.5541681424300582 0.2992107495073461 0.9071768598656667 T T T
0.9649129391594045 0.7894776349564054 0.7810599999999965 F F F
0.0701768413975614 0.4210610483853827 0.7445680000000010 F F F
0.8421020476201875 0.0526265116863485 0.7080800000000025 F F F
0.9473680488809180 0.6842082932855078 0.6715879999999999 F F F
0.2982510144811457 0.7894776349564054 0.7810599999999965 F F F
0.4035149167193097 0.4210610483853827 0.7445680000000010 F F F
0.1754401229419287 0.0526265116863485 0.7080800000000025 F F F
0.2807061242026592 0.6842082932855078 0.6715879999999999 F F F
0.6315772348318589 0.7894776349564054 0.7810599999999965 F F F
0.7368411370700230 0.4210610483853827 0.7445680000000010 F F F
0.5087663432926490 0.0526265116863485 0.7080800000000025 F F F
0.6140323445533795 0.6842082932855078 0.6715879999999999 F F F
0.0526319511190820 0.3157917067144922 0.6350959999999972 F F F
0.7192938757973408 0.3157917067144922 0.6350959999999972 F F F
0.8245598770580713 0.9473734883136515 0.5986079999999987 F F F
0.1578979523798125 0.9473734883136515 0.5986079999999987 F F F
0.3859700264408232 0.3157917067144922 0.6350959999999972 F F F
0.4912241727305329 0.9473734883136515 0.5986079999999987 F F F
0.9941414431656530 0.9450226780214843 0.8754559980690828 T T T
0.0910313055894631 0.5326077401403903 0.8406433576121226 T T T
0.3273869833624458 0.9391225152029457 0.8724186767965265 T T T
0.4217592873790652 0.5324867943360840 0.8408030021942396 T T T
0.6580957428984605 0.9388646296184083 0.8725437298816423 T T T
0.7583892351711956 0.5327857416340944 0.8408866882779733 T T T
0.8622517343059639 0.1606126917612856 0.8083837374873032 T T T
0.1936034543999002 0.1608977782995480 0.8083512713922792 T T T
0.5288964953267598 0.1634426386601351 0.8085184332435781 T T T
0.0433621274718001 0.1048659446750580 0.0508764487743648 T T T
0.9493889017970111 0.1531733138107413 0.0651953239443953 T T T
Surface(331)-4 layers
1.00000000000000
8.4352800000000006 0.0000000000000000 0.0000000000000000
-1.4058800000000000 6.1280900000000003 0.0000000000000000
0.0000000000000000 0.0000000000000000 25.0000000000000000
Pt Cu O H
27 9 1 1
Selective dynamics
Direct
0.0038626601676110 -0.0182791678472280 -0.0176920894452949 T T T
0.1108304871198005 0.6406086820597965 0.9434395174614313 T T T
0.8866734884211418 0.2753349823074577 0.9158529534388405 T T T
0.3409273333671181 -0.0150544935480997 -0.0244715389659799 T T T
0.4434578251710707 0.6332121972605110 0.9453453868210248 T T T
0.2167413771636857 0.2776003417344309 0.9155725644955901 T T T
0.6670059379134167 -0.0181511614660629 -0.0241826578124769 T T T
0.7821576456488368 0.6386596225038214 0.9438541592986671 T T T
0.5533676787121009 0.2831924629227330 0.9167863323435952 T T T
0.9649129391594045 0.7894776349564054 0.7810599999999965 F F F
0.0701768413975614 0.4210610483853827 0.7445680000000010 F F F
0.8421020476201875 0.0526265116863485 0.7080800000000025 F F F
0.9473680488809180 0.6842082932855078 0.6715879999999999 F F F
0.2982510144811457 0.7894776349564054 0.7810599999999965 F F F
0.4035149167193097 0.4210610483853827 0.7445680000000010 F F F
0.1754401229419287 0.0526265116863485 0.7080800000000025 F F F
0.2807061242026592 0.6842082932855078 0.6715879999999999 F F F
0.6315772348318589 0.7894776349564054 0.7810599999999965 F F F
0.7368411370700230 0.4210610483853827 0.7445680000000010 F F F
0.5087663432926490 0.0526265116863485 0.7080800000000025 F F F
0.6140323445533795 0.6842082932855078 0.6715879999999999 F F F
0.0526319511190820 0.3157917067144922 0.6350959999999972 F F F
0.7192938757973408 0.3157917067144922 0.6350959999999972 F F F
0.8245598770580713 0.9473734883136515 0.5986079999999987 F F F
0.1578979523798125 0.9473734883136515 0.5986079999999987 F F F
0.3859700264408232 0.3157917067144922 0.6350959999999972 F F F
0.4912241727305329 0.9473734883136515 0.5986079999999987 F F F
0.9907707534077347 0.9152102986213031 0.8794192235605116 T T T
0.0898931166168713 0.5253169857073996 0.8448658344162298 T T T
0.3250934298478307 0.9128293593555619 0.8764063803468815 T T T
0.4228574236540665 0.5259822444562223 0.8455832895041382 T T T
0.6549211986043366 0.9118584869027445 0.8765165598468851 T T T
0.7570219860164289 0.5239428408320016 0.8450649789416992 T T T
0.8619815610329357 0.1581440632276815 0.8126027551692131 T T T
0.1943606956266834 0.1583115255935712 0.8125018192471265 T T T
0.5284984248389413 0.1605876176618193 0.8127958783425909 T T T
0.0293347262485490 0.0999140283239460 0.0553696445944431 T T T
-0.0780979585638554 0.1019608103092710 0.0698163961670685 T T T
Surface(331)-4 layers
1.00000000000000
8.4352800000000006 0.0000000000000000 0.0000000000000000
-1.4058800000000000 6.1280900000000003 0.0000000000000000
0.0000000000000000 0.0000000000000000 25.0000000000000000
Pt Zn O H
27 9 1 1
Selective dynamics
Direct
0.0031171239229418 0.0171668013285053 -0.0144298863869837 T T T
0.1060046609805220 0.6442684267768166 0.9453847149304200 T T T
0.8802062669557813 0.2730018995337192 0.9134130285237959 T T T
0.3346595154509029 -0.0029835819825053 -0.0202411057080267 T T T
0.4397024862379688 0.6411835945404427 0.9455867243115809 T T T
0.2103594572003787 0.2739529396327935 0.9126009725619768 T T T
0.6619335485074396 -0.0053172345602307 -0.0201107095438059 T T T
0.7750706775883272 0.6430206797137451 0.9447591554551019 T T T
0.5470794498024245 0.2777003909799263 0.9097359567331398 T T T
0.9649129391594045 0.7894776349564054 0.7810599999999965 F F F
0.0701768413975614 0.4210610483853827 0.7445680000000010 F F F
0.8421020476201875 0.0526265116863485 0.7080800000000025 F F F
0.9473680488809180 0.6842082932855078 0.6715879999999999 F F F
0.2982510144811457 0.7894776349564054 0.7810599999999965 F F F
0.4035149167193097 0.4210610483853827 0.7445680000000010 F F F
0.1754401229419287 0.0526265116863485 0.7080800000000025 F F F
0.2807061242026592 0.6842082932855078 0.6715879999999999 F F F
0.6315772348318589 0.7894776349564054 0.7810599999999965 F F F
0.7368411370700230 0.4210610483853827 0.7445680000000010 F F F
0.5087663432926490 0.0526265116863485 0.7080800000000025 F F F
0.6140323445533795 0.6842082932855078 0.6715879999999999 F F F
0.0526319511190820 0.3157917067144922 0.6350959999999972 F F F
0.7192938757973408 0.3157917067144922 0.6350959999999972 F F F
0.8245598770580713 0.9473734883136515 0.5986079999999987 F F F
0.1578979523798125 0.9473734883136515 0.5986079999999987 F F F
0.3859700264408232 0.3157917067144922 0.6350959999999972 F F F
0.4912241727305329 0.9473734883136515 0.5986079999999987 F F F
0.9853686486236358 0.9111985501983297 0.8830581078126903 T T T
0.0875584831698328 0.5274947558565637 0.8455611603771561 T T T
0.3186142413104531 0.9109209673860476 0.8812281901778813 T T T
0.4208246076188374 0.5272690827275920 0.8451520159749163 T T T
0.6514359279913906 0.9090703228462886 0.8812525159282413 T T T
0.7558244881348583 0.5280444545345577 0.8450812397615759 T T T
0.8592522698297528 0.1534209406943729 0.8087268061202727 T T T
0.1918172262653032 0.1534415141409201 0.8086385839758621 T T T
0.5255880537876919 0.1534099809053628 0.8081428551464919 T T T
-0.0196943144408448 -0.0765585990979582 0.0614663257525219 T T T
0.0165796498161187 0.0588038117098834 0.0818723426338870 T T T
Surface(331)-4 layers
1.00000000000000
8.4352800000000006 0.0000000000000000 0.0000000000000000
-1.4058800000000000 6.1280900000000003 0.0000000000000000
0.0000000000000000 0.0000000000000000 25.0000000000000000
Pt Nb O H
27 9 1 1
Selective dynamics
Direct
0.0085584987272411 0.0457599060470144 0.0047238793405399 T T T
0.1080000845684363 0.6534459823647258 0.9662924103539304 T T T
0.8823643455209675 0.2871197605092435 0.9283856939649590 T T T
0.3403089385345217 0.0156439700118640 0.0018551306644381 T T T
0.4412286459887387 0.6496590875182724 0.9660309898696950 T T T
0.2133307629762639 0.2875878482499074 0.9291778801477756 T T T
0.6662378350809292 0.0144900466317302 0.0014474272863680 T T T
0.7770227017121381 0.6526645971959900 0.9657781493396078 T T T
0.5483948974994480 0.2928560149365338 0.9262497730350200 T T T
0.9649129391594045 0.7894776349564054 0.7810599999999965 F F F

```
  0.0701768413975614  0.4210610483853827  0.7445680000000010   F   F   F
  0.8421020476201875  0.0526265116863485  0.7080800000000025   F   F   F
  0.9473680488809180  0.6842082932855078  0.6715879999999999   F   F   F
  0.2982510144811457  0.7894776349564054  0.7810599999999965   F   F   F
  0.4035149167193097  0.4210610483853827  0.7445680000000010   F   F   F
  0.1754401229419287  0.0526265116863485  0.7080800000000025   F   F   F
  0.2807061242026592  0.6842082932855078  0.6715879999999999   F   F   F
  0.6315772348318589  0.7894776349564054  0.7810599999999965   F   F   F
  0.7368411370700230  0.4210610483853827  0.7445680000000010   F   F   F
  0.5087663432926490  0.0526265116863485  0.7080800000000025   F   F   F
  0.6140323445533795  0.6842082932855078  0.6715879999999999   F   F   F
  0.0526319511190820  0.3157917067144922  0.6350959999999972   F   F   F
  0.7192938757973408  0.3157917067144922  0.6350959999999972   F   F   F
  0.8245598770580713  0.9473734883136515  0.5986079999999987   F   F   F
  0.1578979523798125  0.9473734883136515  0.5986079999999987   F   F   F
  0.3859700264408232  0.3157917067144922  0.6350959999999972   F   F   F
  0.4912241727305329  0.9473734883136515  0.5986079999999987   F   F   F
  0.9865532427155952  0.9200333605048575  0.9013785140034011   T   T   T
  0.0900591323344772  0.5356779695549481  0.8561307362631618   T   T   T
  0.3214436226317383  0.9253650027765807  0.9008789995579500   T   T   T
  0.4243081765757977  0.5399646935935277  0.8547396117057215   T   T   T
  0.6536786100015139  0.9226648605279661  0.9003395250576626   T   T   T
  0.7559034966050339  0.5402685422034078  0.8549431815232205   T   T   T
  0.8588756855244022  0.1501755912189805  0.8125054624163927   T   T   T
  0.1909104309772984  0.1494526236184056  0.8125764971581526   T   T   T
  0.5250015014936158  0.1497074937026237  0.8123368695250686   T   T   T
 -0.0232611029597917 -0.1148518838937240  0.0746582105362337   T   T   T
 -0.0054744549941025  0.0001104206594766  0.1019021709183425   T   T   T
Surface(331)-4 layers
  1.00000000000000
    8.4352800000000006    0.0000000000000000    0.0000000000000000
   -1.4058800000000000    6.1280900000000003    0.0000000000000000
    0.0000000000000000    0.0000000000000000   25.0000000000000000
  Pt   Mo   O    H
   27     9     1     1
Selective dynamics
Direct
  0.0022683907204371  0.0107897366080037 -0.0067146331745168   T   T   T
  0.1058464281248079  0.6358689650069794  0.9565935980003689   T   T   T
  0.8803896943176633  0.2713372751271881  0.9201443792073352   T   T   T
  0.3374533984652396 -0.0025546381175345 -0.0093956521572755   T   T   T
  0.4381848445039441  0.6312786608487854  0.9566016250941407   T   T   T
  0.2093526139957355  0.2688974408302944  0.9202172789557488   T   T   T
  0.6640772473942791 -0.0025581302207867 -0.0091875744667754   T   T   T
  0.7731951354733486  0.6344812962696581  0.9565859079279011   T   T   T
  0.5454079321669159  0.2736213683548815  0.9190235184217440   T   T   T
  0.9649129391594045  0.7894776349564054  0.7810599999999965   F   F   F
  0.0701768413975614  0.4210610483853827  0.7445680000000010   F   F   F
  0.8421020476201875  0.0526265116863485  0.7080800000000025   F   F   F
  0.9473680488809180  0.6842082932855078  0.6715879999999999   F   F   F
  0.2982510144811457  0.7894776349564054  0.7810599999999965   F   F   F
  0.4035149167193097  0.4210610483853827  0.7445680000000010   F   F   F
  0.1754401229419287  0.0526265116863485  0.7080800000000025   F   F   F
  0.2807061242026592  0.6842082932855078  0.6715879999999999   F   F   F
  0.6315772348318589  0.7894776349564054  0.7810599999999965   F   F   F
  0.7368411370700230  0.4210610483853827  0.7445680000000010   F   F   F
  0.5087663432926490  0.0526265116863485  0.7080800000000025   F   F   F
  0.6140323445533795  0.6842082932855078  0.6715879999999999   F   F   F
  0.0526319511190820  0.3157917067144922  0.6350959999999972   F   F   F
  0.7192938757973408  0.3157917067144922  0.6350959999999972   F   F   F
  0.8245598770580713  0.9473734883136515  0.5986079999999987   F   F   F
  0.1578979523798125  0.9473734883136515  0.5986079999999987   F   F   F
  0.3859700264408232  0.3157917067144922  0.6350959999999972   F   F   F
  0.4912241727305329  0.9473734883136515  0.5986079999999987   F   F   F
  0.9822159357445066  0.9058131142450492  0.8908048047979130   T   T   T
  0.0885017358694850  0.5252583785731848  0.8509188541314676   T   T   T
  0.3198204974942986  0.9082375243580875  0.8901028131307138   T   T   T
  0.4237866675227107  0.5280047991122401  0.8499613524536005   T   T   T
  0.6506352245209107  0.9058124568779982  0.8903039562339823   T   T   T
  0.7521093468635962  0.5285878148814261  0.8502615890480589   T   T   T
  0.8605916385593428  0.1501035806225021  0.8116233153175151   T   T   T
  0.1906509961026598  0.1500807511405733  0.8114769326566348   T   T   T
  0.5251305026312362  0.1497690357351881  0.8114397047503652   T   T   T
 -0.0120082032016863 -0.1213789525156079  0.0666284004179438   T   T   T
 -0.1250717652940896 -0.1509394742731427  0.0786878182962959   T   T   T
Surface(331)-4 layers
  1.00000000000000
    8.4352800000000006    0.0000000000000000    0.0000000000000000
   -1.4058800000000000    6.1280900000000003    0.0000000000000000
    0.0000000000000000    0.0000000000000000   25.0000000000000000
  Pt   Tc   O    H
   27     9     1     1
Selective dynamics
Direct
 -0.0021136463415270 -0.0016573465683829 -0.0100897339390989   T   T   T
  0.1043400764601875  0.6310721315124828  0.9540615317301177   T   T   T
  0.8793208902252245  0.2694971195575168  0.9180262594060241   T   T   T
  0.3338681442973792 -0.0134902746464587 -0.0144833164934924   T   T   T
  0.4382240212037606  0.6286465490631562  0.9528183659059496   T   T   T
  0.2101020171042325  0.2694890571085968  0.9183585348306960   T   T   T
  0.6619150953050106 -0.0129480573025903 -0.0145417220641838   T   T   T
  0.7722998334430677  0.6304587068472560  0.9534187282029360   T   T   T
  0.5442643379111498  0.2689003170677005  0.9174156166676063   T   T   T
  0.9649129391594045  0.7894776349564054  0.7810599999999965   F   F   F
  0.0701768413975614  0.4210610483853827  0.7445680000000010   F   F   F
  0.8421020476201875  0.0526265116863485  0.7080800000000025   F   F   F
  0.9473680488809180  0.6842082932855078  0.6715879999999999   F   F   F
  0.2982510144811457  0.7894776349564054  0.7810599999999965   F   F   F
  0.4035149167193097  0.4210610483853827  0.7445680000000010   F   F   F
  0.1754401229419287  0.0526265116863485  0.7080800000000025   F   F   F
  0.2807061242026592  0.6842082932855078  0.6715879999999999   F   F   F
  0.6315772348318589  0.7894776349564054  0.7810599999999965   F   F   F
  0.7368411370700230  0.4210610483853827  0.7445680000000010   F   F   F
  0.5087663432926490  0.0526265116863485  0.7080800000000025   F   F   F
  0.6140323445533795  0.6842082932855078  0.6715879999999999   F   F   F
  0.0526319511190820  0.3157917067144922  0.6350959999999972   F   F   F
  0.7192938757973408  0.3157917067144922  0.6350959999999972   F   F   F
  0.8245598770580713  0.9473734883136515  0.5986079999999987   F   F   F
  0.1578979523798125  0.9473734883136515  0.5986079999999987   F   F   F
  0.3859700264408232  0.3157917067144922  0.6350959999999972   F   F   F
  0.4912241727305329  0.9473734883136515  0.5986079999999987   F   F   F
  0.9835010456394897  0.9003893472788159  0.8870577333668690   T   T   T
  0.0876495880534671  0.5310279321811878  0.8492433620084180   T   T   T
  0.3051674734343385  0.9058131914399036  0.8827697045680664   T   T   T
  0.4181949709114700  0.5161076266679759  0.8479421867550481   T   T   T
  0.6616764684714364  0.9061742486866501  0.8829848366122929   T   T   T
  0.7552041424496441  0.5231692876311724  0.8483859007209986   T   T   T
  0.8506884595936101  0.1534580007573821  0.8145728132500144   T   T   T
  0.2024474872326423  0.1555529002084164  0.8149054834535345   T   T   T
  0.5263154425438697  0.1518071817930773  0.8133624749201375   T   T   T
 -0.0011408884891713 -0.0129543379469692  0.0706083119751956   T   T   T
  0.0225967370760259  0.1354330816066740  0.0848736591899173   T   T   T
Surface(331)-4 layers
  1.00000000000000
    8.4352800000000006    0.0000000000000000    0.0000000000000000
   -1.4058800000000000    6.1280900000000003    0.0000000000000000
    0.0000000000000000    0.0000000000000000   25.0000000000000000
  Pt   Ru   O    H
   27     9     1     1
Selective dynamics
Direct
 -0.0011583118885054 -0.0071656686944212 -0.0118335714476924   T   T   T
  0.1046221089715371  0.6333300635620899  0.9521575563761669   T   T   T
  0.8784010582708507  0.2708057210520555  0.9185623177894042   T   T   T
  0.3329668259530346 -0.0129016007135326 -0.0155873465340215   T   T   T
  0.4380379305325028  0.6296108444488100  0.9532725292093696   T   T   T
  0.2119107373423632  0.2705424619321544  0.9187525570719405   T   T   T
  0.6626306233162373 -0.0122185051779134 -0.0155725880160015   T   T   T
  0.7737004720586721  0.6337378729615172  0.9523489412814597   T   T   T
  0.5456082465517142  0.2739425453345719  0.9184755598691634   T   T   T
  0.9649129391594045  0.7894776349564054  0.7810599999999965   F   F   F
  0.0701768413975614  0.4210610483853827  0.7445680000000010   F   F   F
  0.8421020476201875  0.0526265116863485  0.7080800000000025   F   F   F
  0.9473680488809180  0.6842082932855078  0.6715879999999999   F   F   F
  0.2982510144811457  0.7894776349564054  0.7810599999999965   F   F   F
  0.4035149167193097  0.4210610483853827  0.7445680000000010   F   F   F
  0.1754401229419287  0.0526265116863485  0.7080800000000025   F   F   F
  0.2807061242026592  0.6842082932855078  0.6715879999999999   F   F   F
  0.6315772348318589  0.7894776349564054  0.7810599999999965   F   F   F
  0.7368411370700230  0.4210610483853827  0.7445680000000010   F   F   F
  0.5087663432926490  0.0526265116863485  0.7080800000000025   F   F   F
  0.6140323445533795  0.6842082932855078  0.6715879999999999   F   F   F
  0.0526319511190820  0.3157917067144922  0.6350959999999972   F   F   F
  0.7192938757973408  0.3157917067144922  0.6350959999999972   F   F   F
  0.8245598770580713  0.9473734883136515  0.5986079999999987   F   F   F
  0.1578979523798125  0.9473734883136515  0.5986079999999987   F   F   F
  0.3859700264408232  0.3157917067144922  0.6350959999999972   F   F   F
  0.4912241727305329  0.9473734883136515  0.5986079999999987   F   F   F
  0.9854059013210181  0.9131007369598764  0.8835973949611472   T   T   T
  0.0877294966965106  0.5232398928459184  0.8476048007095277   T   T   T
  0.3181488443172269  0.9078712775091675  0.8815323473936285   T   T   T
  0.4204002341604507  0.5245571455564852  0.8481261080158706   T   T   T
  0.6509235442739092  0.9060343450505052  0.8814830746754412   T   T   T
  0.7537665702802586  0.5234140401949546  0.8479518583416539   T   T   T
  0.8596823539903921  0.1545493410543922  0.8153873674179635   T   T   T
  0.1915315844973932  0.1550434796407008  0.8153710280316253   T   T   T
  0.5265876602262316  0.1551279821542189  0.8151917551472583   T   T   T
 -0.0008886741928875 -0.0113608788161736  0.0686591704083280   T   T   T
  0.0215796273347416  0.1386027221728761  0.0818780226696735   T   T   T
Surface(331)-4 layers
  1.00000000000000
    8.4352800000000006    0.0000000000000000    0.0000000000000000
   -1.4058800000000000    6.1280900000000003    0.0000000000000000
    0.0000000000000000    0.0000000000000000   25.0000000000000000
  Pt   Rh   O    H
   27     9     1     1
Selective dynamics
Direct
 -0.0049947286747431 -0.0157385947455554 -0.0097568457287354   T   T   T
  0.1077721482570977  0.6333522131234897  0.9540301277025542   T   T   T
  0.8802213757775980  0.2750781087972939  0.9229768418674583   T   T   T
  0.3358172877743760 -0.0129533664759411 -0.0148023327260228   T   T   T
  0.4381489471599125  0.6294956925008429  0.9552277519571855   T   T   T
  0.2125855564932611  0.2727578599432606  0.9227285991051596   T   T   T
  0.6617781253878899 -0.0127539515089537 -0.0143260273522317   T   T   T
  0.7767907013173938  0.6376676885033118  0.9542803063856969   T   T   T
  0.5486216795063341  0.2802248841261895  0.9230818688238576   T   T   T
  0.9649129391594045  0.7894776349564054  0.7810599999999965   F   F   F
  0.0701768413975614  0.4210610483853827  0.7445680000000010   F   F   F
  0.8421020476201875  0.0526265116863485  0.7080800000000025   F   F   F
  0.9473680488809180  0.6842082932855078  0.6715879999999999   F   F   F
  0.2982510144811457  0.7894776349564054  0.7810599999999965   F   F   F
  0.4035149167193097  0.4210610483853827  0.7445680000000010   F   F   F
  0.1754401229419287  0.0526265116863485  0.7080800000000025   F   F   F
  0.2807061242026592  0.6842082932855078  0.6715879999999999   F   F   F
  0.6315772348318589  0.7894776349564054  0.7810599999999965   F   F   F
  0.7368411370700230  0.4210610483853827  0.7445680000000010   F   F   F
  0.5087663432926490  0.0526265116863485  0.7080800000000025   F   F   F
  0.6140323445533795  0.6842082932855078  0.6715879999999999   F   F   F
  0.0526319511190820  0.3157917067144922  0.6350959999999972   F   F   F
  0.7192938757973408  0.3157917067144922  0.6350959999999972   F   F   F
  0.8245598770580713  0.9473734883136515  0.5986079999999987   F   F   F
  0.1578979523798125  0.9473734883136515  0.5986079999999987   F   F   F
  0.3859700264408232  0.3157917067144922  0.6350959999999972   F   F   F
  0.4912241727305329  0.9473734883136515  0.5986079999999987   F   F   F
  0.9861965237716069  0.9108473486677207  0.8850216749074082   T   T   T
  0.0870755847267170  0.5240204813509630  0.8488246770141863   T   T   T
  0.3190393608769506  0.9060264789290745  0.8821352550927835   T   T   T
  0.4203627725885235  0.5238116283027925  0.8492630642338507   T   T   T
  0.6511435515671379  0.9061410751618703  0.8821797923583670   T   T   T
  0.7548857874463755  0.5247018805263783  0.8490671654737191   T   T   T
  0.8607764309553597  0.1583988560554264  0.8149499149761735   T   T   T
  0.1919101998677269  0.1574225083290314  0.8148433569607815   T   T   T
  0.5268111599994565  0.1596300511262678  0.8150459073379370   T   T   T
  0.0531083460863455  0.0890874643559145  0.0639593460294247   T   T   T
 -0.0129271240216295  0.1914117487301281  0.0747099460627375   T   T   T
Surface(331)-4 layers
  1.00000000000000
    8.4352800000000006    0.0000000000000000    0.0000000000000000
   -1.4058800000000000    6.1280900000000003    0.0000000000000000
    0.0000000000000000    0.0000000000000000   25.0000000000000000
  Pt   Pd   O    H
   27     9     1     1
Selective dynamics
Direct
 -0.0008709368226719 -0.0172758265779015 -0.0049095052472116   T   T   T
  0.1100222820720764  0.6303743335117230  0.9619833164338127   T   T   T
  0.8837205105648787  0.2775350118268366  0.9303937782200156   T   T   T
  0.3398700885146501 -0.0171115870474630 -0.0097373756551747   T   T   T
  0.4410572468030236  0.6264576941493784  0.9620512213581879   T   T   T
  0.2150519953913416  0.2760520853551018  0.9301390561920749   T   T   T
  0.6639997609483014 -0.0165173063868837 -0.0091550485594884   T   T   T
  0.7776840282591554  0.6323971778895482  0.9623593655785151   T   T   T
  0.5520477571946230  0.2812413005028696  0.9306141725685122   T   T   T
  0.9649129391594045  0.7894776349564054  0.7810599999999965   F   F   F
  0.0701768413975614  0.4210610483853827  0.7445680000000010   F   F   F
  0.8421020476201875  0.0526265116863485  0.7080800000000025   F   F   F
  0.9473680488809180  0.6842082932855078  0.6715879999999999   F   F   F
  0.2982510144811457  0.7894776349564054  0.7810599999999965   F   F   F
  0.4035149167193097  0.4210610483853827  0.7445680000000010   F   F   F
  0.1754401229419287  0.0526265116863485  0.7080800000000025   F   F   F
  0.2807061242026592  0.6842082932855078  0.6715879999999999   F   F   F
  0.6315772348318589  0.7894776349564054  0.7810599999999965   F   F   F
  0.7368411370700230  0.4210610483853827  0.7445680000000010   F   F   F
  0.5087663432926490  0.0526265116863485  0.7080800000000025   F   F   F
  0.6140323445533795  0.6842082932855078  0.6715879999999999   F   F   F
  0.0526319511190820  0.3157917067144922  0.6350959999999972   F   F   F
  0.7192938757973408  0.3157917067144922  0.6350959999999972   F   F   F
  0.8245598770580713  0.9473734883136515  0.5986079999999987   F   F   F
  0.1578979523798125  0.9473734883136515  0.5986079999999987   F   F   F
  0.3859700264408232  0.3157917067144922  0.6350959999999972   F   F   F
  0.4912241727305329  0.9473734883136515  0.5986079999999987   F   F   F
  0.9878626227792136  0.9080450195692380  0.8901554813452699   T   T   T
  0.0894669759564766  0.5288614112801338  0.8533516534102720   T   T   T
  0.3208581280328403  0.9056794159633261  0.8859358465405127   T   T   T
  0.4226474701655680  0.5257987733105771  0.8533655193890662   T   T   T
  0.6531672178401151  0.9050816176898621  0.8860118610710935   T   T   T
  0.7577312296085127  0.5283016790739266  0.8535588392854284   T   T   T
  0.8623989017634871  0.1606165207584462  0.8163524186585469   T   T   T
  0.1934670163371061  0.1607064291855102  0.8162389212766471   T   T   T
  0.5285538596971306  0.1629167561139837  0.8161114025451938   T   T   T
  0.0405635422123882  0.1062666633014870  0.0673898804368982   T   T   T
 -0.0453222210323822  0.1820763097602917  0.0765139544768634   T   T   T
Surface(331)-4 layers
  1.00000000000000
    8.4352800000000006    0.0000000000000000    0.0000000000000000
   -1.4058800000000000    6.1280900000000003    0.0000000000000000
    0.0000000000000000    0.0000000000000000   25.0000000000000000
  Pt   Ag   O    H
   27     9     1     1
Selective dynamics
Direct
  0.0056893447224105 -0.0099300275088610  0.0100244753309248   T   T   T
  0.1131176298530998  0.6471831208086533  0.9721770732648222   T   T   T
  0.8887898558874919  0.2893890145079958  0.9489314388376796   T   T   T
  0.3420878555699943 -0.0082850722313071  0.0029933766141575   T   T   T
  0.4448169750709596  0.6429278033748188  0.9727270558088016   T   T   T
  0.2186422824253406  0.2905116810785963  0.9478575703892622   T   T   T
  0.6680052560892312 -0.0115856762780325  0.0035830546707061   T   T   T
  0.7833058524779047  0.6464980587424686  0.9726867259389319   T   T   T
  0.5561929196212961  0.2958894808385877  0.9500109885361384   T   T   T
  0.9649129391594045  0.7894776349564054  0.7810599999999965   F   F   F
  0.0701768413975614  0.4210610483853827  0.7445680000000010   F   F   F
  0.8421020476201875  0.0526265116863485  0.7080800000000025   F   F   F
  0.9473680488809180  0.6842082932855078  0.6715879999999999   F   F   F
  0.2982510144811457  0.7894776349564054  0.7810599999999965   F   F   F
  0.4035149167193097  0.4210610483853827  0.7445680000000010   F   F   F
  0.1754401229419287  0.0526265116863485  0.7080800000000025   F   F   F
  0.2807061242026592  0.6842082932855078  0.6715879999999999   F   F   F
  0.6315772348318589  0.7894776349564054  0.7810599999999965   F   F   F
  0.7368411370700230  0.4210610483853827  0.7445680000000010   F   F   F
  0.5087663432926490  0.0526265116863485  0.7080800000000025   F   F   F
  0.6140323445533795  0.6842082932855078  0.6715879999999999   F   F   F
  0.0526319511190820  0.3157917067144922  0.6350959999999972   F   F   F
  0.7192938757973408  0.3157917067144922  0.6350959999999972   F   F   F
  0.8245598770580713  0.9473734883136515  0.5986079999999987   F   F   F
  0.1578979523798125  0.9473734883136515  0.5986079999999987   F   F   F
  0.3859700264408232  0.3157917067144922  0.6350959999999972   F   F   F
  0.4912241727305329  0.9473734883136515  0.5986079999999987   F   F   F
  0.9902465262171379  0.9220612813435350  0.8976745258364659   T   T   T
  0.0903801835157115  0.5327312391549569  0.8608044238592175   T   T   T
  0.3234175194149863  0.9169514287451915  0.8939696769132696   T   T   T
  0.4238392969105676  0.5294018371408734  0.8612000957619613   T   T   T
  0.6554742918079168  0.9165102463236426  0.8940605390409931   T   T   T
  0.7585381749999384  0.5312564836393576  0.8613439736026767   T   T   T
  0.8630399562702147  0.1647236919666585  0.8202509484637424   T   T   T
  0.1946929982426265  0.1644363842574582  0.8201325663645229   T   T   T
  0.5293343106495160  0.1655991298367663  0.8196383538281847   T   T   T
  0.0298929882469347  0.0894483961137968  0.0844329821572201   T   T   T
 -0.0766865699649770  0.1023798242968600  0.0978061416309497   T   T   T
Surface(331)-4 layers
  1.00000000000000
    8.4352800000000006    0.0000000000000000    0.0000000000000000
   -1.4058800000000000    6.1280900000000003    0.0000000000000000
    0.0000000000000000    0.0000000000000000   25.0000000000000000
  Pt   Cd   O    H
   27     9     1     1
Selective dynamics
Direct
  0.0111432279334180  0.0578983874054218  0.0199766809000443   T   T   T
  0.1151392897051351  0.6921686619828622  0.9796248261339615   T   T   T
  0.8897162852153372  0.3322098661003356  0.9524546257720580   T   T   T
  0.3435988550432555  0.0461786672165285  0.0135610795384947   T   T   T
  0.4481352606464302  0.6913060317804715  0.9793231582944292   T   T   T
  0.2206046957943083  0.3334735587878832  0.9515152812867180   T   T   T
  0.6699066482354381  0.0442191172014592  0.0139122069970120   T   T   T
  0.7824073038235497  0.6921948786179100  0.9790546198376980   T   T   T
  0.5569622980964883  0.3374606225693567  0.9479491136514993   T   T   T
  0.9649129391594045  0.7894776349564054  0.7810599999999965   F   F   F
  0.0701768413975614  0.4210610483853827  0.7445680000000010   F   F   F
  0.8421020476201875  0.0526265116863485  0.7080800000000025   F   F   F
  0.9473680488809180  0.6842082932855078  0.6715879999999999   F   F   F
  0.2982510144811457  0.7894776349564054  0.7810599999999965   F   F   F
  0.4035149167193097  0.4210610483853827  0.7445680000000010   F   F   F
  0.1754401229419287  0.0526265116863485  0.7080800000000025   F   F   F
  0.2807061242026592  0.6842082932855078  0.6715879999999999   F   F   F
  0.6315772348318589  0.7894776349564054  0.7810599999999965   F   F   F
  0.7368411370700230  0.4210610483853827  0.7445680000000010   F   F   F
  0.5087663432926490  0.0526265116863485  0.7080800000000025   F   F   F
  0.6140323445533795  0.6842082932855078  0.6715879999999999   F   F   F
  0.0526319511190820  0.3157917067144922  0.6350959999999972   F   F   F
  0.7192938757973408  0.3157917067144922  0.6350959999999972   F   F   F
  0.8245598770580713  0.9473734883136515  0.5986079999999987   F   F   F
  0.1578979523798125  0.9473734883136515  0.5986079999999987   F   F   F
  0.3859700264408232  0.3157917067144922  0.6350959999999972   F   F   F
  0.4912241727305329  0.9473734883136515  0.5986079999999987   F   F   F
  0.9944528354383640  0.9666769008047259  0.9085900045179758   T   T   T
  0.0917604258806557  0.5528194407817236  0.8639174369969274   T   T   T
  0.3267250847473913  0.9620225341776603  0.9058899423979221   T   T   T
  0.4249742210553030  0.5522242548611641  0.8626653033729716   T   T   T
  0.6607188297448247  0.9619497410531853  0.9060372959310995   T   T   T
  0.7603928079310809  0.5548114894437248  0.8631639135273714   T   T   T
  0.8602063651429531  0.1590647043042996  0.8171090367577408   T   T   T
  0.1926038768537957  0.1591039440913023  0.8171510070615464   T   T   T
  0.5269775416405502  0.1607526533463988  0.8174623273665299   T   T   T
 -0.0183612834848302 -0.0685456052478066  0.0930196418974392   T   T   T
  0.0137259062379683  0.0582002230434226  0.1166514752321428   T   T   T
Surface(331)-4 layers
  1.00000000000000
    8.4352800000000006    0.0000000000000000    0.0000000000000000
   -1.4058800000000000    6.1280900000000003    0.0000000000000000
    0.0000000000000000    0.0000000000000000   25.0000000000000000
  Pt   Ta   O    H
   27     9     1     1
Selective dynamics
Direct
  0.0084455810991339  0.0473711134510954  0.0041781836948657   T   T   T
  0.1098036803128911  0.6638879296003013  0.9647893027501668   T   T   T
  0.8847388028691580  0.2952439293517565  0.9278575904829748   T   T   T
  0.3417464704668655  0.0243936961633743  0.0009373236620320   T   T   T
  0.4429901320707504  0.6598630595425595  0.9643716741638553   T   T   T
  0.2137551403610405  0.2957396660914435  0.9285635617644120   T   T   T
  0.6679599481730170  0.0233168735695530  0.0006126868881071   T   T   T
  0.7783917325363284  0.6626724199315998  0.9642238613701944   T   T   T
  0.5496119469488988  0.3004569636426423  0.9256333032302315   T   T   T
  0.9649129391594045  0.7894776349564054  0.7810599999999965   F   F   F
  0.0701768413975614  0.4210610483853827  0.7445680000000010   F   F   F
  0.8421020476201875  0.0526265116863485  0.7080800000000025   F   F   F
  0.9473680488809180  0.6842082932855078  0.6715879999999999   F   F   F
  0.2982510144811457  0.7894776349564054  0.7810599999999965   F   F   F
  0.4035149167193097  0.4210610483853827  0.7445680000000010   F   F   F
  0.1754401229419287  0.0526265116863485  0.7080800000000025   F   F   F
  0.2807061242026592  0.6842082932855078  0.6715879999999999   F   F   F
  0.6315772348318589  0.7894776349564054  0.7810599999999965   F   F   F
  0.7368411370700230  0.4210610483853827  0.7445680000000010   F   F   F
  0.5087663432926490  0.0526265116863485  0.7080800000000025   F   F   F
  0.6140323445533795  0.6842082932855078  0.6715879999999999   F   F   F
  0.0526319511190820  0.3157917067144922  0.6350959999999972   F   F   F
  0.7192938757973408  0.3157917067144922  0.6350959999999972   F   F   F
  0.8245598770580713  0.9473734883136515  0.5986079999999987   F   F   F
```

0.1578979523798125 0.9473734883136515 0.5986079999999987 F F F
0.3859700264408232 0.3157917067144922 0.6350959999999972 F F F
0.4912241727305329 0.9473734883136515 0.5986079999999987 F F F
0.9883945173059416 0.9300264788738442 0.9006606727900317 T T T
0.0903843663636481 0.5370407791879227 0.8554498193301012 T T T
0.3228629254650054 0.9356281121248307 0.9000008507423000 T T T
0.4238438569047518 0.5403948678092265 0.8539675075769660 T T T
0.6555924739554302 0.9329967259859212 0.8997005879584726 T T T
0.7567992397487049 0.5422182267995286 0.8543384552326264 T T T
0.8587913358043080 0.1492525779500996 0.8118417248683585 T T T
0.1908247519585144 0.1482172689785977 0.8117695676729177 T T T
0.5250404370052636 0.1485683496454671 0.8115669431804737 T T T
-0.0268625242057281 -0.1303442960763473 0.0721517686322448 T T T
-0.0074376923673616 -0.0234867999882367 0.1013365848043417 T T T
Surface(331)-4 layers
1.00000000000000
8.4352800000000006 0.0000000000000000 0.0000000000000000
-1.4058800000000000 6.1280900000000003 0.0000000000000000
0.0000000000000000 0.0000000000000000 25.0000000000000000
Pt W O H
27 9 1 1
Selective dynamics
Direct
0.0008867477272455 0.0128569682623578 -0.0057166295028513 T T T
0.1056503646522852 0.6380753027711576 0.9573204057345901 T T T
0.8807257040191632 0.2725464604012063 0.9205361385472254 T T T
0.3362331312089722 0.0005654957346161 -0.0083691848635582 T T T
0.4387639479311211 0.6347278541262986 0.9571705169575786 T T T
0.2099882842875324 0.2714771675943513 0.9207278562156509 T T T
0.6635406320905896 0.0002999127621451 -0.0083665762101965 T T T
0.7735491520019258 0.6376753753742230 0.9568414257777413 T T T
0.5454748457032353 0.2749260538033940 0.9196423722016203 T T T
0.9649129391594045 0.7894776349564054 0.7810599999999965 F F F
0.0701768413975614 0.4210610483853827 0.7445680000000010 F F F
0.8421020476201875 0.0526265116863485 0.7080800000000025 F F F
0.9473680488809180 0.6842082932855078 0.6715879999999999 F F F
0.2982510144811457 0.7894776349564054 0.7810599999999965 F F F
0.4035149167193097 0.4210610483853827 0.7445680000000010 F F F
0.1754401229419287 0.0526265116863485 0.7080800000000025 F F F
0.2807061242026592 0.6842082932855078 0.6715879999999999 F F F
0.6315772348318589 0.7894776349564054 0.7810599999999965 F F F
0.7368411370700230 0.4210610483853827 0.7445680000000010 F F F
0.5087663432926490 0.0526265116863485 0.7080800000000025 F F F
0.6140323445533795 0.6842082932855078 0.6715879999999999 F F F
0.0526319511190820 0.3157917067144922 0.6350959999999972 F F F
0.7192938757973408 0.3157917067144922 0.6350959999999972 F F F
0.8245598770580713 0.9473734883136515 0.5986079999999987 F F F
0.1578979523798125 0.9473734883136515 0.5986079999999987 F F F
0.3859700264408232 0.3157917067144922 0.6350959999999972 F F F
0.4912241727305329 0.9473734883136515 0.5986079999999987 F F F
0.9847363368092700 0.9083670808348638 0.8918252269079570 T T T
0.0884768074594060 0.5260790016003756 0.8512774845706387 T T T
0.3189822931265535 0.9096164270749654 0.8911354396124467 T T T
0.4231331152647617 0.5286778068370102 0.8503984076636099 T T T
0.6510903472533938 0.9083082621169699 0.8910053315841868 T T T
0.7528777228387524 0.5289468906990643 0.8505734618707719 T T T
0.8593272370936147 0.1501369159398954 0.8113463067778773 T T T
0.1906116889243210 0.1498727854397562 0.8112277772808741 T T T
0.5249684883358847 0.1502675843713234 0.8111686639257796 T T T
-0.0141308021164680 -0.1318900643629592 0.0663690560502814 T T T
-0.1280367797618227 -0.1712344754090095 0.0775539051475838 T T T
Surface(331)-4 layers
1.00000000000000
8.4352800000000006 0.0000000000000000 0.0000000000000000
-1.4058800000000000 6.1280900000000003 0.0000000000000000
0.0000000000000000 0.0000000000000000 25.0000000000000000
Pt Re O H
27 9 1 1
Selective dynamics
Direct
-0.0021571939906291 -0.0002204438152674 -0.0090055934342963 T T T
0.1032059375677051 0.6295280775398434 0.9552150729358639 T T T
0.8783158427138824 0.2670736806105155 0.9183801485509309 T T T
0.3334694977081111 -0.0136519759794964 -0.0136315223005532 T T T
0.4382774080259240 0.6275451766455887 0.9538005495747528 T T T
0.2099544259952134 0.2671818370211740 0.9188658166332817 T T T
0.6619804091364523 -0.0123353821227759 -0.0135955052809571 T T T
0.7721879106926403 0.6289831647377888 0.9547621213071900 T T T
0.5439169921318683 0.2662382225307603 0.9178510950367093 T T T
0.9649129391594045 0.7894776349564054 0.7810599999999965 F F F
0.0701768413975614 0.4210610483853827 0.7445680000000010 F F F
0.8421020476201875 0.0526265116863485 0.7080800000000025 F F F
0.9473680488809180 0.6842082932855078 0.6715879999999999 F F F
0.2982510144811457 0.7894776349564054 0.7810599999999965 F F F
0.4035149167193097 0.4210610483853827 0.7445680000000010 F F F
0.1754401229419287 0.0526265116863485 0.7080800000000025 F F F
0.2807061242026592 0.6842082932855078 0.6715879999999999 F F F
0.6315772348318589 0.7894776349564054 0.7810599999999965 F F F
0.7368411370700230 0.4210610483853827 0.7445680000000010 F F F
0.5087663432926490 0.0526265116863485 0.7080800000000025 F F F
0.6140323445533795 0.6842082932855078 0.6715879999999999 F F F
0.0526319511190820 0.3157917067144922 0.6350959999999972 F F F
0.7192938757973408 0.3157917067144922 0.6350959999999972 F F F
0.8245598770580713 0.9473734883136515 0.5986079999999987 F F F
0.1578979523798125 0.9473734883136515 0.5986079999999987 F F F
0.3859700264408232 0.3157917067144922 0.6350959999999972 F F F
0.4912241727305329 0.9473734883136515 0.5986079999999987 F F F
0.9825844340835187 0.8997714334838850 0.8881021226678343 T T T
0.0874368299342892 0.5295723980067444 0.8495638469200685 T T T
0.3033107691467923 0.8977046122174372 0.8839004329086615 T T T
0.4187524628861398 0.5195793317943561 0.8485206080333715 T T T
0.6600499289212143 0.8979047583978745 0.8842100665998734 T T T
0.7546553825883975 0.5243861156768024 0.8488376247539490 T T T
0.8507875566747392 0.1551781514958508 0.8142500012470006 T T T
0.2032422219845936 0.1571360749688805 0.8148662321403972 T T T
0.5273253750804595 0.1524897623922111 0.8127359875981122 T T T
0.0009200117811972 -0.0114840344552234 0.0720767790734912 T T T
0.0216800308966673 0.1361838265034450 0.0867817833639377 T T T
Surface(331)-4 layers
1.00000000000000
8.4352800000000006 0.0000000000000000 0.0000000000000000
-1.4058800000000000 6.1280900000000003 0.0000000000000000
0.0000000000000000 0.0000000000000000 25.0000000000000000
Pt Os O H
27 9 1 1
Selective dynamics
Direct
-0.0068776550661704 -0.0117519902685643 -0.0100884486361197 T T T
0.1044773802148000 0.6357153478219018 0.9538544231836312 T T T
0.8776326092900195 0.2724701437643601 0.9195020754264405 T T T
0.3332943261268562 -0.0108192230555882 -0.0148710875338660 T T T
0.4366558048774680 0.6288811849840595 0.9547384631052478 T T T
0.2115942600150408 0.2704061060689289 0.9195203332348955 T T T
0.6598048834698411 -0.0114146897713663 -0.0144832140994196 T T T
0.7749191435158184 0.6374162476665214 0.9539002566534130 T T T
0.5455594640716209 0.2746320771476160 0.9195783305925551 T T T
0.9649129391594045 0.7894776349564054 0.7810599999999965 F F F
0.0701768413975614 0.4210610483853827 0.7445680000000010 F F F
0.8421020476201875 0.0526265116863485 0.7080800000000025 F F F
0.9473680488809180 0.6842082932855078 0.6715879999999999 F F F
0.2982510144811457 0.7894776349564054 0.7810599999999965 F F F
0.4035149167193097 0.4210610483853827 0.7445680000000010 F F F
0.1754401229419287 0.0526265116863485 0.7080800000000025 F F F
0.2807061242026592 0.6842082932855078 0.6715879999999999 F F F
0.6315772348318589 0.7894776349564054 0.7810599999999965 F F F
0.7368411370700230 0.4210610483853827 0.7445680000000010 F F F
0.5087663432926490 0.0526265116863485 0.7080800000000025 F F F
0.6140323445533795 0.6842082932855078 0.6715879999999999 F F F
0.0526319511190820 0.3157917067144922 0.6350959999999972 F F F
0.7192938757973408 0.3157917067144922 0.6350959999999972 F F F
0.8245598770580713 0.9473734883136515 0.5986079999999987 F F F
0.1578979523798125 0.9473734883136515 0.5986079999999987 F F F
0.3859700264408232 0.3157917067144922 0.6350959999999972 F F F
0.4912241727305329 0.9473734883136515 0.5986079999999987 F F F
0.9838525040511222 0.9050705057767549 0.8831865024865801 T T T
0.0878933737684924 0.5248581147399988 0.8483102719105914 T T T
0.3173592836514438 0.9018941340653798 0.8818712943805418 T T T
0.4203054456811344 0.5249779761692754 0.8485310745240019 T T T
0.6495675968058342 0.9035243245628735 0.8819020949632479 T T T
0.7531243953034543 0.5239301216512320 0.8484360760292524 T T T
0.8599795269487903 0.1571638245232443 0.8166097479506293 T T T
0.1918832103635897 0.1561936130439301 0.8166122814227936 T T T
0.5261787395610463 0.1580227290924342 0.8164945118313512 T T T
0.0442219763423070 0.1751347629105460 0.0544543650866496 T T T
-0.0453751977397111 0.2458287028765166 0.0620465603977435 T T T
Surface(331)-4 layers
1.00000000000000
8.4352800000000006 0.0000000000000000 0.0000000000000000
-1.4058800000000000 6.1280900000000003 0.0000000000000000
0.0000000000000000 0.0000000000000000 25.0000000000000000
Pt Ir O H
27 9 1 1
Selective dynamics
Direct
0.9952407793597744 0.9904841096006139 0.9914803173942230 T T T
0.1071404373985001 0.6359204163031078 0.9553181312779356 T T T
0.8792098966662676 0.2752414787500074 0.9234188693464799 T T T
0.3352191782400861 0.9912567467157747 0.9866052318951111 T T T
0.4378414377644346 0.6322787591402340 0.9567196463648971 T T T
0.2120383591615044 0.2726256118319607 0.9232320936106806 T T T
0.6616423526383168 0.9912895578410724 0.9869618249626551 T T T
0.7768884533588578 0.6408640397796891 0.9556600048434145 T T T
0.5480214958038674 0.2798279451074626 0.9235419655437170 T T T
0.9649129391594045 0.7894776349564054 0.7810599999999965 F F F
0.0701768413975614 0.4210610483853827 0.7445680000000010 F F F
0.8421020476201875 0.0526265116863485 0.7080800000000025 F F F
0.9473680488809180 0.6842082932855078 0.6715879999999999 F F F
0.2982510144811457 0.7894776349564054 0.7810599999999965 F F F
0.4035149167193097 0.4210610483853827 0.7445680000000010 F F F
0.1754401229419287 0.0526265116863485 0.7080800000000025 F F F
0.2807061242026592 0.6842082932855078 0.6715879999999999 F F F
0.6315772348318589 0.7894776349564054 0.7810599999999965 F F F
0.7368411370700230 0.4210610483853827 0.7445680000000010 F F F
0.5087663432926490 0.0526265116863485 0.7080800000000025 F F F
0.6140323445533795 0.6842082932855078 0.6715879999999999 F F F
0.0526319511190820 0.3157917067144922 0.6350959999999972 F F F
0.7192938757973408 0.3157917067144922 0.6350959999999972 F F F
0.8245598770580713 0.9473734883136515 0.5986079999999987 F F F
0.1578979523798125 0.9473734883136515 0.5986079999999987 F F F
0.3859700264408232 0.3157917067144922 0.6350959999999972 F F F
0.4912241727305329 0.9473734883136515 0.5986079999999987 F F F
0.9849020361517755 0.9065657535581496 0.8854621391414371 T T T
0.0871205712848864 0.5243245320690100 0.8493298443358570 T T T
0.3182215040511202 0.9017334191693160 0.8829322800749365 T T T
0.4203832558479174 0.5242568373120217 0.8497138581130919 T T T
0.6501868388039524 0.9023865344978707 0.8828109835371649 T T T
0.7549717563746753 0.5252883027753854 0.8494952156621068 T T T
0.8613499382308712 0.1603354968757492 0.8162033406948026 T T T
0.1923905541288607 0.1589335101276333 0.8160995287749468 T T T
0.5270263917821002 0.1610513522764160 0.8162901252776661 T T T
0.0572585205829970 0.0988887752509877 0.0648455151312398 T T T
0.0010483355978952 0.2136968883587684 0.0743351534871095 T T T
Surface(331)-4 layers
1.00000000000000
8.4352800000000006 0.0000000000000000 0.0000000000000000
-1.4058800000000000 6.1280900000000003 0.0000000000000000
0.0000000000000000 0.0000000000000000 25.0000000000000000
Pt O H
36 1 1
Selective dynamics
Direct
-0.0083270134163418 -0.0285747154818363 -0.0030901297145144 T T T
0.1033033568240248 0.6185447843013211 0.9637199405511864 T T T
0.8770320059792920 0.2658564803698958 0.9314714876284237 T T T
0.3323086813242611 -0.0260213516146176 -0.0079352782618639 T T T
0.4336737166053541 0.6155668072626108 0.9643016582703250 T T T
0.2078553882930649 0.2632293470760070 0.9310065699966650 T T T
0.6572114374606759 -0.0257256460258944 -0.0075942336655106 T T T
0.7709741748646695 0.6222253531738677 0.9638562828196613 T T T
0.5451428405260217 0.2701431875053888 0.9313014882486135 T T T
0.9649129391594045 0.7894776349564054 0.7810599999999965 F F F
0.0701768413975614 0.4210610483853827 0.7445680000000010 F F F
0.8421020476201875 0.0526265116863485 0.7080800000000025 F F F
0.9473680488809180 0.6842082932855078 0.6715879999999999 F F F
0.2982510144811457 0.7894776349564054 0.7810599999999965 F F F
0.4035149167193097 0.4210610483853827 0.7445680000000010 F F F
0.1754401229419287 0.0526265116863485 0.7080800000000025 F F F
0.2807061242026592 0.6842082932855078 0.6715879999999999 F F F
0.6315772348318589 0.7894776349564054 0.7810599999999965 F F F
0.7368411370700230 0.4210610483853827 0.7445680000000010 F F F
0.5087663432926490 0.0526265116863485 0.7080800000000025 F F F
0.6140323445533795 0.6842082932855078 0.6715879999999999 F F F
0.0526319511190820 0.3157917067144922 0.6350959999999972 F F F
0.7192938757973408 0.3157917067144922 0.6350959999999972 F F F
0.8245598770580713 0.9473734883136515 0.5986079999999987 F F F
0.1578979523798125 0.9473734883136515 0.5986079999999987 F F F
0.3859700264408232 0.3157917067144922 0.6350959999999972 F F F
0.4912241727305329 0.9473734883136515 0.5986079999999987 F F F
0.9825830414764519 0.8977478719457189 0.8910336463532003 T T T
0.0859715017236966 0.5234020137251965 0.8541207599903934 T T T
0.3165036281773293 0.8969235625726741 0.8869388457595323 T T T
0.4194010567344506 0.5210807226206299 0.8544999890107173 T T T
0.6485438252314957 0.8975460018827380 0.8867999105672300 T T T
0.7549277088717770 0.5235978596164166 0.8542355027310385 T T T
0.8608842666665738 0.1596893121494670 0.8170643249469494 T T T
0.1916226468777316 0.1595636516660807 0.8170464021725408 T T T
0.5270669355831741 0.1624907480059378 0.8166519984325431 T T T
0.0469660530046699 0.0847412957463029 0.0698280867100191 T T T
0.0021303386260620 0.2142350028227642 0.0748993423150033 T T T
Surface(331)-4 layers
1.00000000000000
8.4352800000000006 0.0000000000000000 0.0000000000000000
-1.4058800000000000 6.1280900000000003 0.0000000000000000
0.0000000000000000 0.0000000000000000 25.0000000000000000
Pt Au O H
27 9 1 1
Selective dynamics
Direct
0.0077681545236847 0.0076047879905397 0.0119204426074328 T T T
0.1171452793392344 0.6598352968085039 0.9764195397295152 T T T
0.8911213985035594 0.3054752993546326 0.9495597198258577 T T T
0.3458945480208752 0.0086392738143095 0.0066146485658571 T T T
0.4484476068238257 0.6568511335021530 0.9767465772709868 T T T
0.2225294541734844 0.3049253682427121 0.9491023243482569 T T T
0.6716687490767789 0.0068377030722338 0.0070507791025265 T T T
0.7843566243266984 0.6591225839544784 0.9769624695232565 T T T
0.5586594231913167 0.3089732864873259 0.9508294049500698 T T T
0.9649129391594045 0.7894776349564054 0.7810599999999965 F F F
0.0701768413975614 0.4210610483853827 0.7445680000000010 F F F
0.8421020476201875 0.0526265116863485 0.7080800000000025 F F F
0.9473680488809180 0.6842082932855078 0.6715879999999999 F F F
0.2982510144811457 0.7894776349564054 0.7810599999999965 F F F
0.4035149167193097 0.4210610483853827 0.7445680000000010 F F F
0.1754401229419287 0.0526265116863485 0.7080800000000025 F F F
0.2807061242026592 0.6842082932855078 0.6715879999999999 F F F
0.6315772348318589 0.7894776349564054 0.7810599999999965 F F F
0.7368411370700230 0.4210610483853827 0.7445680000000010 F F F
0.5087663432926490 0.0526265116863485 0.7080800000000025 F F F
0.6140323445533795 0.6842082932855078 0.6715879999999999 F F F
0.0526319511190820 0.3157917067144922 0.6350959999999972 F F F
0.7192938757973408 0.3157917067144922 0.6350959999999972 F F F
0.8245598770580713 0.9473734883136515 0.5986079999999987 F F F
0.1578979523798125 0.9473734883136515 0.5986079999999987 F F F
0.3859700264408232 0.3157917067144922 0.6350959999999972 F F F
0.4912241727305329 0.9473734883136515 0.5986079999999987 F F F
0.9924904660206428 0.9358965255598457 0.9016128803711607 T T T
0.0919988399203906 0.5426875957642173 0.8623795960611039 T T T
0.3252846090342418 0.9300216300667292 0.8969548709935302 T T T
0.4254436980303085 0.5387256951731245 0.8626771143065490 T T T
0.6576007113480365 0.9289715048233099 0.8970263307680434 T T T
0.7603085576844343 0.5411899687292580 0.8630457393972072 T T T
0.8636854994710687 0.1675877034382610 0.8202211990148998 T T T
0.1948598644404819 0.1671136141150344 0.8201679312191853 T T T
0.5298264555525151 0.1691495980635470 0.8196230810688373 T T T
0.0333588224961706 0.1119863043115698 0.0857575415041386 T T T
-0.0710328717535355 0.1388880157361871 0.0977400262531155 T T T
Surface(331)-4 layers
1.00000000000000
8.4352800000000006 0.0000000000000000 0.0000000000000000
-1.4058800000000000 6.1280900000000003 0.0000000000000000
0.0000000000000000 0.0000000000000000 25.0000000000000000
Pt Hg O H
27 9 1 1
Selective dynamics
Direct
0.0290569824087048 0.1776630612157732 0.0508262360448882 T T T
0.1407978714049462 0.8594749399713439 -0.0105000399329355 T T T
0.9180511014074926 0.4986318466788339 0.0054109461275153 T T T
0.3631722883037374 0.1677567828554217 0.0420369389516001 T T T
0.4750748713312963 0.8565808923925560 -0.0104101617856356 T T T
0.2464069491592611 0.4968602567275043 0.0039931146213710 T T T
0.6917356844211321 0.1668202735413312 0.0422893246739565 T T T
0.8115550402712480 0.8595740537839124 -0.0109862907608429 T T T
0.5831698355978937 0.5027209460613130 0.0047225295218888 T T T
0.9649129391594045 0.7894776349564054 0.7810599999999965 F F F
0.0701768413975614 0.4210610483853827 0.7445680000000010 F F F
0.8421020476201875 0.0526265116863485 0.7080800000000025 F F F
0.9473680488809180 0.6842082932855078 0.6715879999999999 F F F
0.2982510144811457 0.7894776349564054 0.7810599999999965 F F F
0.4035149167193097 0.4210610483853827 0.7445680000000010 F F F
0.1754401229419287 0.0526265116863485 0.7080800000000025 F F F
0.2807061242026592 0.6842082932855078 0.6715879999999999 F F F
0.6315772348318589 0.7894776349564054 0.7810599999999965 F F F
0.7368411370700230 0.4210610483853827 0.7445680000000010 F F F
0.5087663432926490 0.0526265116863485 0.7080800000000025 F F F
0.6140323445533795 0.6842082932855078 0.6715879999999999 F F F
0.0526319511190820 0.3157917067144922 0.6350959999999972 F F F
0.7192938757973408 0.3157917067144922 0.6350959999999972 F F F
0.8245598770580713 0.9473734883136515 0.5986079999999987 F F F
0.1578979523798125 0.9473734883136515 0.5986079999999987 F F F
0.3859700264408232 0.3157917067144922 0.6350959999999972 F F F
0.4912241727305329 0.9473734883136515 0.5986079999999987 F F F
0.0345260673165130 0.2099307996626483 0.9313085455537701 T T T
0.1177552174230029 0.7036944616987990 0.8809130338082739 T T T
0.3687890936132913 0.2112985815463226 0.9276915788311012 T T T
0.4502750331732840 0.6990938942209044 0.8806014592107209 T T T
0.7036976776976070 0.2250107191789120 0.9278390228721473 T T T
0.7862294478738673 0.7163189747718811 0.8806464701974213 T T T
0.8656481049353513 0.1888625400365742 0.8244712046768108 T T T
0.1968443193125623 0.1830851735604797 0.8241584457278242 T T T
0.5301348358878661 0.1781721871528291 0.8232027908176950 T T T
-0.0150213032265099 -0.0326600086410965 0.1112633914903552 T T T
0.0122630035989600 0.0557314145232483 0.1436490669784004 T T T

References

[1] G. Kresse, J. Furthmüller, *Phys. Rev. B* **1996**, *54*, 11169.

[2] G. Kresse, D. Joubert, *Phys. Rev. B* **1999**, *59*, 1758.

[3] J. P. Perdew, K. Burke, M. Ernzerhof, *Phys. Rev. Lett.* **1996**, *77*, 3865.

[4] H. J. Monkhorst, J. D. Pack, *Phys. Rev. B* **1976**, *13*, 5188.

[5] F. Calle-Vallejo, M. D. Pohl, A. S. Bandarenka, *ACS Catal.* **2017**, *7*, 4355.

[6] F. Calle-Vallejo, M. T. M. Koper, *Angew. Chem., Int. Ed.* **2013**, *52*, 7282.

[7] J. K. Nørskov, J. Rossmeisl, A. Logadottir, L. Lindqvist, J. R. Kitchin, T. Bligaard, H. Jónsson, *J. Phys. Chem. B* **2004**, *108*, 17886.

[8] F. Calle-Vallejo, J. I. Martínez, J. M. García-Lastra, J. Rossmeisl, M. T. M. Koper, *Phys. Rev. Lett.* **2012**, *108*, 116103.
